# Nanopore analysis of *cis*-diols in fruits

**DOI:** 10.1038/s41467-024-46303-x

**Published:** 2024-03-05

**Authors:** Pingping Fan, Zhenyuan Cao, Shanyu Zhang, Yuqin Wang, Yunqi Xiao, Wendong Jia, Panke Zhang, Shuo Huang

**Affiliations:** 1 State Key Laboratory of Analytical Chemistry for Life Sciences, School of Chemistry and Chemical Engineering, Nanjing University, 210023 Nanjing, China; 2 Chemistry and Biomedicine Innovation Center (ChemBIC), Nanjing University, 210023 Nanjing, China; 3 State Key Laboratory of Pollution Control and Resource Reuse, School of the Environment, Nanjing University, 210023 Nanjing, China; 4 Institute for the Environment and Health, Nanjing University Suzhou Campus, 215163 Suzhou, China

**Keywords:** Nanopores, Bioanalytical chemistry

## Abstract

Natural fruits contain a large variety of *cis*-diols. However, due to the lack of a high-resolution sensor that can simultaneously identify all *cis*-diols without a need of complex sample pretreatment, direct and rapid analysis of fruits in a hand-held device has never been previously reported. Nanopore, a versatile single molecule sensor, can be specially engineered to perform this task. A hetero-octameric *Mycobacterium smegmatis* porin A (MspA) nanopore modified with a sole phenylboronic acid (PBA) adapter is prepared. This engineered MspA accurately recognizes 1,2-diphenols, alditols, α-hydroxy acids and saccharides in prune, grape, lemon, different varieties of kiwifruits and commercial juice products. Assisted with a custom machine learning program, an accuracy of 99.3% is reported and the sample pretreatment is significantly simplified. Enantiomers such as DL-malic acids can also be directly identified, enabling sensing of synthetic food additives. Though demonstrated with fruits, these results suggest wide applications of nanopore in food and drug administration uses.

## Abstract (web summary)

Fruits contain a large variety of *cis*-diols but their rapid and direct analysis without complex sample pretreatment was not achieved. Here, the authors report a *Mycobacterium smegmatis* porin A nanopore modified with a phenylboronic acid adapter for recognition of 1,2-diphenols, alditols, α-hydroxy acids and saccharides in fruits.

## Introduction

Natural edible substances, including fruits, tea, honey, vegetables and many others contain rich *cis*-diols, such as 1,2-diphenols, alditols, α-hydroxy acids and saccharides^1–4^. A thorough understanding of these compounds in food is essential for nutritional or healthcare purposes^5–7^. Rapid and accurate analysis of food is also used for safety and quality control during food manufacture^8,9^. However, these *cis*-diols from natural sources are invariably a mixture of compounds, which poses a technical challenge for simultaneous analyte identification using a single sensor and under the same condition^10^. Conventionally, high-performance liquid chromatography (HPLC) has been widely applied to the analysis of *cis*-diols in fruits^10^, tea^11,12^ honey^13^ or vegetable^14^, but the chromatographic separation of such mixtures is complex and time consuming and requires equipment that is far from portable. In HPLC analysis, simultaneous detection of a variety of different analytes using the same chromatographic condition is also challenging^15–17^. Other reported analytical methods applicable to *cis*-diols include spectrophotometry^18^, liquid chromatography tandem mass spectrometry (LC-MS/MS)^19,20^ and gas chromatography (GC)^21,22^. The resolution of spectrophotometry is however insufficient for accurate characterization of a variety of *cis*-diols. GC requires complex and poorly reproducible derivatization operations and LC-MS/MS methods require complex sample separation prior to the measurement.

**Fig. 1:**
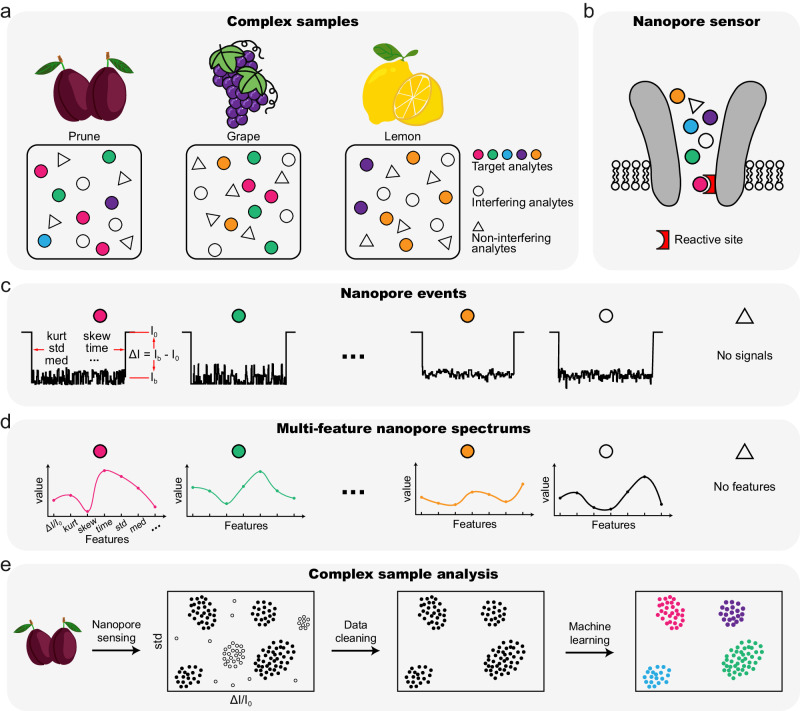
Nanopore analysis of complex samples. **a** The cartoon diagram of complex samples (top) and their chemical components (bottom). The chemical components in complex samples include different combinations of target analytes (colored circles), interfering analytes (uncolored circles) and non-interfering analytes (triangles). **b** The reactive nanopore sensor. A reactive site was introduced to the pore constriction by pore engineering. Both target and interfering analytes report detectable nanopore events. Whereas, the non-interfering analytes cannot be detected. **c** Nanopore events. Acknowledging the high resolution of nanopore, different analytes report unique event characteristics so that target and interfering events can be directly discriminated. Core parameters in the description of event features include open pore current (*I*_*0*_), residual current (*I*_*b*_), standard deviation (std), kurtosis (kurt), skewness (skew), dwell time (time), median (med) as marked on the event. The blockage amplitude (*ΔI*) was defined as *ΔI* = *I*_*b*_ *– I*_*0*_. The blockage ratio was defined as *ΔI/I*_*0*_. **d** Multi-feature nanopore spectrum plotted from the corresponding events of analytes in Fig. 1c. All event features can be extracted and the corresponding multi-feature nanopore spectrums were plotted and used for event identification. **e** The schematics of sample analysis. As an example, natural fruit juice can be directly added to the sensor generating different types of target (solid point) and interfering events (hollow point). A machine learning algorithm serves to automatically identify target and interfering events.

Nanopore, which was originally developed for single molecule sequencing of nucleic acids^23^, is a highly versatile sensor^24–33^. When properly engineered to contain a specific reactive adapter in its lumen, a nanopore immediately becomes reactive and is sensitive to a set of corresponding small molecule analytes^34–36^, which can react reversibly with the adapter to produce successive events. By producing highly characteristic event features for different small molecules, a nanopore can discriminate between different analytes that may be only slightly different in structure^32,37,38^. This sensing capacity is thus suitable for direct and simultaneous analysis of a complex mixture of molecules (Fig. 1).

To use a specific reactive nanopore sensor, any sample can be decomposed into different categories of molecules, including target analytes, interfering analytes and non-interfering analytes (Fig. 1a). Target analytes are molecules of interest that can react with the nanopore to produce corresponding events (Fig. 1b). Simultaneous presence of multiple types of target analytes can be technically tolerated, as long as these target analytes can be fully distinguished by their nanopore event characteristics (Fig. 1c)^32,37,38^. A multi-feature nanopore spectrum, which simultaneously considers multiple event features in a single plot, can be generated and used as a unique signature for a specific type of analyte (Fig. 1d). Meanwhile, the interfering analytes are molecules that are of no interest but can however react with the pore and can also produce nanopore events. However, as long as these events can be fully distinguished from that of all target analytes, their interference in the measurement becomes negligible and these events can subsequently be removed computationally (Fig. 1e). Non-interfering analytes are all molecules that fail to react with the nanopore and thus they don’t produce any events. Even when present in a large quantity, the non-interfering analytes will never appear as nanopore events and their impact can be ignored.

In this work, an MspA nanopore modified with a single phenylboronic acid is prepared. During single channel recording, it serves to reversibly react with *cis*-diols such as 1,2-diphenols, alditols, α-hydroxy acids and saccharides so that highly characteristic nanopore events are produced. Assisted by custom machine learning algorithms, a variety of *cis*-diols in natural fruits can be accurately and simultaneously identified. This sensing capacity also allows for high-resolution discrimination of enantiomeric DL-malic acid, demonstrating immediate applications in the detection of food additives in commercial fruit products. This technique could also be integrated into a miniaturized device, demonstrating its potential for portable sensing.

## Results

### Nanopore signatures of model *cis*-diols

**Fig. 2:**
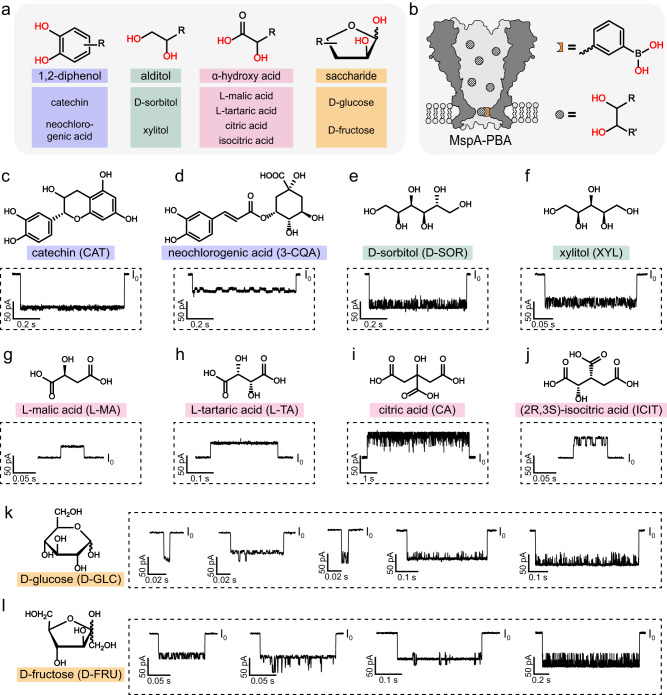
Sensing of target *cis*-diols. **a** Target *cis*-diols investigated in this manuscript. Four kinds of *cis*-diols, including 1,2-diphenols (purple), alditols (green), α-hydroxy acids (pink) and saccharides (orange), all contain *cis*-dihydroxyl groups, as red-marked (top). For each kind of *cis*-diols, a few representative analytes were selected as target *cis*-diols (bottom). **b** The phenylboronic acid (PBA) modified nanopore sensor (Methods). *Cis*-diols reversibly react with PBA (Supplementary Fig. 1), generating characteristic events when sensed by MspA-PBA. **c–l** The chemical structures and corresponding nanopore events of target *cis*-diols, catechin (CAT) (**c**), neochlorogenic acid (3-CQA) (**d**), D-sorbitol (D-SOR) (**e**), xylitol (XYL) (**f**), L-malic acid (L-MA) (**g**), L-tartaric acid (L-TA) (**h**), citric acid (CA) (**i**), (2*R*,3)-isocitric acid (ICIT) (**j**), D-glucose (D-GLC) (**k**) and D-fructose (D-FRU) (**l**). The abbreviations of these *cis*-diols were marked in the corresponding bracket. The open pore currents were marked by *I*_*0*_. All nanopore measurements were performed in a buffer of 1.5 M KCl, 100 mM MOPS, pH 7.0 and a continually applied bias of +160 mV. Each type of *cis*-diol was added as a sole analyte to both *cis* and *trans* chambers (Methods). In this paper, alditols, 1,2-diphenols and α-hydroxy acids all showed only one type of event while saccharides showed several types of events.

Fruits, which contain a mixture of *cis*-diols such as 1,2-diphenols, alditols, α-hydroxy acids and saccharides^39–41^, were used as an example for this demonstration. These *cis*-diols react reversibly with phenylboronic acid (PBA) in an aqueous environment. Ten types of *cis*-diols containing compounds including catechin (CAT), neochlorogenic acid (3-CQA), D-sorbitol (D-SOR), xylitol (XYL), L-malic acid (L-MA), L-tartaric acid (L-TA), citric acid (CA), (2*R*,3*S*)-isocitric acid (ICIT), D-glucose (D-GLC) and D-fructose (D-FRU) were selected as model analytes (Fig. 2a). They represent 1,2-diphenols, alditols, α-hydroxy acids and saccharides that are widely encountered in fruits^39–41^. MspA-PBA, a hetero-octameric MspA nanopore modified with a sole phenylboronic acid (PBA) reactive adapter at site 90 (Fig. 2b), was prepared as previously reported^36,42–44^ (Methods). The PBA at the pore constriction reacts reversibly with *cis*-diols to form a boronate ester^45^, and specifically binds molecules containing *cis*-diols in a heterogenous mixture (Supplementary Fig. 1). At room temperature in an aqueous measurement environment, the capture and release of a *cis*-diol analyte by MspA-PBA can last for more than a few milliseconds, which is ideal for data acquisition by single channel recording. The rate of the reaction can also be finely tuned by changes in the environmental pH and temperature^45–47^. The MspA nanopore, which has a conical lumen geometry that focuses the flow of ionic current to the pore constriction, also provides a high resolution of sensing in the discrimination of structurally similar compounds which has been well demonstrated in nanopore sensing of isomers of saccharides^36^, alditols^43^ and ribonucleotides^42,44^. This high resolution thus enables direct recognition of target molecules from interfering analytes and accordingly, the need for sample separation is minimized.

Unless stated otherwise, all nanopore measurements were carried out in a custom nanopore device which has two chambers separated by a Teflon film containing an aperture of ~100 µm diameter. By convention, the chamber that is electrically grounded is defined as *cis* and its opposing chamber is defined as *trans*. After spontaneously forming a lipid bilayer on the aperture and with MspA-PBA inserted in this bilayer, all nanopore measurements were performed in a buffer of 1.5 M KCl, 100 mM MOPS, pH 7.0 and a continually applied +160 mV bias (Methods). A higher salt concentration and a higher applied bias generally produce nanopore events of a larger amplitude so that a better discrimination resolution is achieved (Supplementary Figs 2, 3). However, a too high applied bias may also generate a high risk of bilayer rupture. A high salt concentration may also lead to introduction of noises. Thus, a 1.5 M KCl buffer and a + 160 mV bias were found to be optimum.

To record the event characteristics of model compounds, each type of *cis*-diol was added as a sole analyte to both *cis* and *trans* chambers with a defined final concentration. All ten model analytes reported fully distinguishable nanopore events (Fig. 2c–l). These events are highly consistent when the same analyte was tested in measurements with different pores. However, their event characteristics are visually different when different compounds were tested. The consistency of event features for each analyte type was also verified by three independent measurements (*N* = 3) performed for each condition (Supplementary Figs 4–13). This further confirms that the high resolution of MspA can well discriminate between different analytes. The nanopore signatures of catechin (CAT), neochlorogenic acid (3-CQA), L-malic acid (L-MA), L-tartaric acid (L-TA), citric acid (CA) and (2*R*,3*S*)-isocitric acid (ICIT) have not been previously reported.

To quantitatively describe sensing events, event parameters including open pore current (*I*_*0*_), residual current (*I*_*b*_), blockage amplitude (*ΔI*), standard deviation (std), kurtosis (kurt), skewness (skew), dwell time (time) and median (med) were used and are defined in Fig. 1c. Results of the concentration dependence assay acquired from all ten model *cis*-diols were summarized in Supplementary Figs 14–23 and Supplementary Table 1. Based on the mechanism of nanopore sensing by single molecule reaction, simultaneous capturing of two *cis*-diols by an MspA-PBA is impossible. Thus, the event features of a specific analyte are independent of the analyte concentration. However, their rate of event appearance is highly concentration dependent and can be used for analyte quantification. The blockage amplitude is defined as *ΔI* = *I*_*b*_ − *I*_*0*_. 1,2-diphenols, alditols and saccharides all appear as negative-going events (*ΔI* < 0, Supplementary Fig. 24) but all α-hydroxy acids report positive-going events (*ΔI* > 0, Supplementary Fig. 24). The phenomenon of positive-going events had been previously observed^46^. The MspA nanopore is extremely sensitive to the presence of additive charges at its pore constriction^48,49^. For instance, the WT MspA, which has negatively charged aspartic acid at its pore constriction, reports a much higher channel conductance than its mutant having asparagine introduced by mutagenesis. The negative charge of α-hydroxy acids should contribute to the increase of the ionic current, but the size of the small molecule analytes fails to provide any significant contribution to the blockage of the ionic current. Thus, binding of α-hydroxy acids generally reports positive-going events. On the other hand, although 1,2-diphenols, alditols and α-hydroxy acids all report a single type of nanopore event, the saccharides instead reported several event types (Fig. 2k, l). This may result from interconversion between their pyranose and furanose forms in an aqueous solution^36,47^, as was observed in our previous study^36^.

Although the event characteristics of different analytes are visually distinct from one another in a 2D event scatter plot of *ΔI/I*_*0*_ and std, their event distributions still slightly overlap (Supplementary Fig. 25). However, when more event features were simultaneously considered, the accuracy of event identification was immediately improved. This is more clearly demonstrated in the multi-feature nanopore spectrums, in which the average means and standard deviations of five event features, including *ΔI/I*_*0*_, std, kurt, skew and time are shown in the same plot (Supplementary Fig. 26, Supplementary Table 2). If necessary, more event features can also be included to further improve the event discrimination.

### Event identification by machine learning

**Fig. 3:**
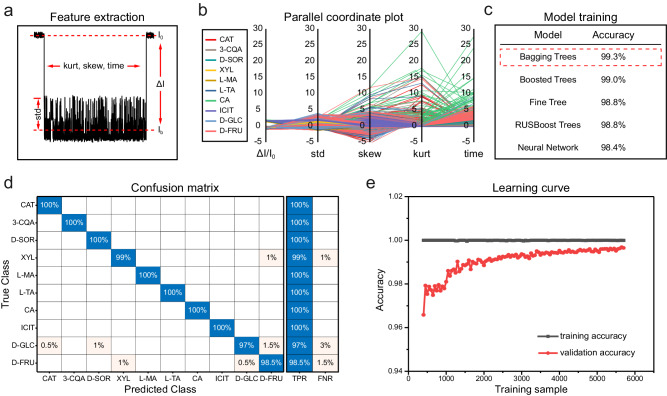
Event identification by machine learning. **a** The definition of event features. Five event features including *ΔI/I*_*0*_, std, kurt, skew and time were extracted for model training and validation. **b** The parallel coordinate plot of five features for ten types of *cis*-diols. All features played a role in the discrimination of different *cis*-diols. The nanopore events of each type of analyte are marked with corresponding color lines, including red (CAT), brown (3-CQA), dark green (D-SOR), yellow (XYL), claybank (L-MA), dark blue (L-TA), green (CA), purple (ICIT), blue (D-GLC) and pink (D-FRU). **c** The validation accuracies of different models. The validation accuracies were evaluated by 10-fold cross-validation. After training with different models however with the same training set, the model of Bagging Trees showed the highest accuracy of 99.3% and it was selected for all further operations. **d** The confusion matrix plot of the testing set generated by the trained Bagging Trees model. The true positive rate (TPR) and the false negative rate (FNR) were demonstrated on the right. **e** The learning curves of the Bagging Trees model generated by varying the size of the input training samples. The validation accuracy increased with an increasing size of the input training samples. Eventually, the validation accuracy gradually approached the training accuracy. Data in the learning curves were presented as mean values derived from three independent measurements. Source data are provided as a Source Data file.

To enable automatic and unbiased event identification based on results from the five-feature nanopore spectra, a custom machine learning algorithm was developed. All machine learning was performed in the Classification Learner toolbox of MATLAB. Five event features, including *ΔI/I*_*0*_, std, kurt, skew and time (Fig. 3a) were extracted to form a feature matrix. The label of each event was assigned as the type of the sole analyte used for data acquisition. The event features of a total of 5600 events from all ten types of model analytes were collected to form the training set which was used for model training (Methods). In the parallel coordinate plot, all five features contribute to the discrimination between events (Fig. 3b). According to the validation accuracies produced by different classification models, the Bagging Trees model reported the highest accuracy of 99.3% (Fig. 3c, Supplementary Table 3).

To test the generality of the model, an additional 1120 events, which didn’t participate in the model training, were used as a testing set. The results of event prediction for the testing set are summarized in Supplementary Table 3, in which the testing accuracy of most models was >80%. During testing, the Bagging Trees model again reported the highest accuracy of 99.1%. Thus, if not otherwise stated, the previously trained Bagging Trees model was selected as the optimum model for all future event predictions. The confusion matrix plot of the testing set generated by prediction of the Bagging Trees model is shown in Fig. 3d. The learning curves are also shown, from which the model is confirmed to be not overfitting and an input training data of 1000 is enough to gain a >98% prediction accuracy (Fig. 3e). The machine learning algorithm, which simultaneously considers five event features in the event identification, is shown to be accurate, efficient and unbiased. Ten *cis*-diols were also simultaneously measured using MspA-PBA in a 1.5 M KCl buffer with a continually applied +160 mV bias. Representative traces acquired at this condition were presented in Supplementary Fig. 27. All events were identified by the trained Bagging Trees model and the events on the trace were labelled accordingly. The resolution of the pore and the performance of the machine learning model are thus well approved.

### Rapid analysis of natural fruit juice by a nanopore

**Fig. 4:**
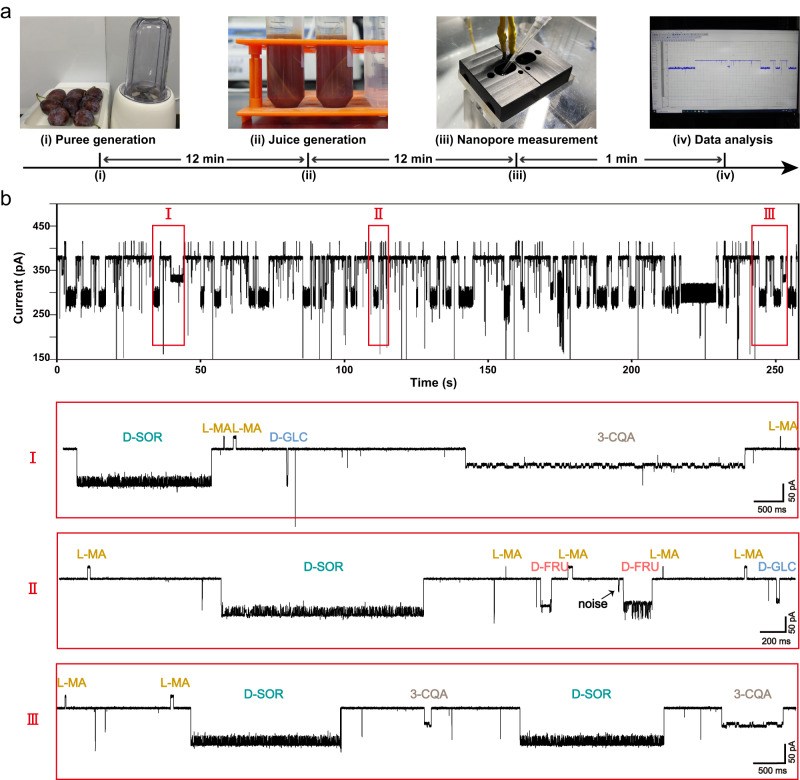
Rapid analysis of natural prune juice. **a** The procedures and the timeline of operations. (i) Puree generation. Prunes (left) were thoroughly treated by a mini food processor (right) to produce puree. (ii) Juice generation. The puree was centrifuged at 1900 g for 10 min and the supernatant was collected and ultrafiltration treated. (iii) Nanopore measurement. The filtrate was loaded to both *cis* and *trans* chambers and nanopore measurements were carried out. (iv) Data analysis. The corresponding nanopore events were further analyzed. The nanopore measurements were performed in a 1.5 M KCl buffer with a continually applied bias of +160 mV. 5 μL prune juice was added to both measurement chambers and thoroughly stirred. **b** A representative trace segment acquired with prune juice. The top figure is a 260 s trace segment. Zoomed-in views of different sections of the trace, which are marked with red boxes and roman numerals, are respectively demonstrated in the bottom. Events of 3-CQA, D-SOR, L-MA, D-GLC and D-FRU were respectively identified by machine learning and labelled on the trace.

**Fig. 5:**
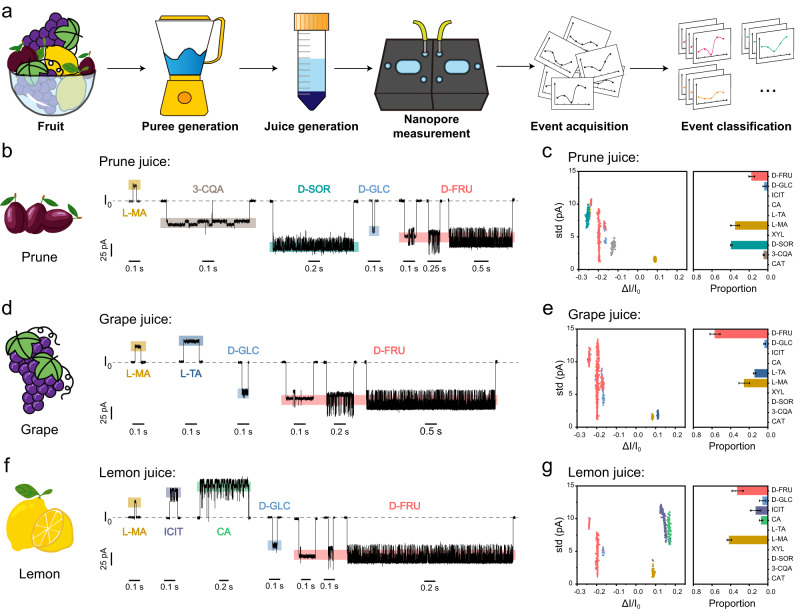
Rapid analysis of natural fruit juice. **a** The schematic diagram of rapid analysis of natural fruit juice. The fruit was first treated by a food processor to produce puree. The puree was further centrifuged at 1900 g for 10 min to collect the juice. The supernatant was ultrafiltration treated. The filtrate was then added to both nanopore measurement chambers to initiate the measurement. The nanopore measurements were performed using MspA-PBA in a 1.5 M KCl buffer with a continually applied bias of +160 mV. The nanopore events were then identified by the previously trained machine learning model. **b**, **d**, **f** Representative events acquired with the juice of (**b**) prune, (**d**) grape or (**f**) lemon. **c**, **e**, **g** The scatter plots of *ΔI/I*_*0*_ versus std of events acquired with the juice of (**c**) prune (*n* = 1770), (**e**) grape (*n* = 1732) or (**g**) lemon (*n* = 957). To remove background events, the events of prune, grape or lemon juice were respectively treated by cluster analysis as described in Supplementary Figs 28, 33 and 34. The identity of each event was predicted by the previously trained machine learning program. The proportion of events for each corresponding set of scatter plot data was produced and placed to the right of the scatter plot. Data in the bar plots (**c**, **e**, **g**) were presented as mean ± standard deviation values derived from results of three independent measurements (*N* = 3). The error bars represent standard deviation values. Source data are provided as a Source Data file.

The nanopore event characteristics of model analytes and the corresponding machine learning algorithm were used in the rapid analysis of *cis*-diol components in natural fruit juice. With prunes as the first example, a work flow of nanopore analysis of prune juice is summarized in Fig. 4a. Briefly, prunes were first pureed in a food processor and then centrifuged at 1900 g for 10 min to separate the solid material from the juice. To further remove solid residues, the juice was placed in an ultracentrifugation tube with a 3 kDa molecular weight cut off (MWCO) and then centrifuged at 2350 g for 10 min. The filtrate was collected and directly added to the nanopore device (Methods). Subsequently, the corresponding nanopore events were observed immediately (Fig. 4b). The results of nanopore measurements with natural fruit juice also report background noise events, appearing as non-clustered distributions in the scatter plot (Supplementary Fig. 28). To remove interference from them, a cluster analysis algorithm based on Density-Based Spatial Clustering of Applications with Noise (DBSCAN)^50^ was introduced to computationally and automatically remove these interfering events (Supplementary Fig. 28). Subsequently, the trained Bagging Trees model was used to identify nanopore events in prune juice, enabling identification of 3-CQA, D-SOR, L-MA, D-GLC and D-FRU (Fig. 5b, c, Supplementary Movie 1). Three independent trails were performed at the same measurement condition and highly consistent results were shown (Supplementary Fig. 29). The high resolution of event discrimination by nanopore further guarantees that each type of model compound can be recognized reliably. These sensing features of nanopore enable direct measurement of fruit samples and without complicated sample separation (Fig. 4a). Instead, when the same juice was sensed by M2 MspA^48^, which contains no PBA adapter, no nanopore events with well-defined features were observed (Supplementary Fig. 30). This further confirms that an unmodified MspA pore fails to provide any useful information for analysis of fruit juice and that the PBA modification is critical in the generation of *cis*-diol events.

The operations demonstrated above were also similarly used with grape or lemon juice. Following corresponding nanopore measurements using MspA-PBA (Supplementary Figs 31, 32), background noise reduction with DBSCAN (Supplementary Figs 33, 34) and machine learning based event identification by the Bagging Trees model, the *cis*-diol components in grape and lemon juice were all accurately recognized. For grapes, the *cis*-diols identified were L-MA, L-TA, D-GLC, D-FRU (Fig. 5d, e, Supplementary Fig. 35 and Supplementary Movie 2), which are consistent with the results obtained by HPLC^51^ and for lemon, the identified events were from L-MA, CA, ICIT, D-GLC and D-FRU (Fig. 5f, g, Supplementary Fig. 36 and Supplementary Movie 3). Quantification of relevant *cis*-diols in above discussed fruit juices were also performed as described in Methods. The corresponding results were also presented in Supplementary Tables 4, 5, according to which grape has the highest D-fructose content and lemon has the highest CA content, respectively.

According to these results, L-MA, D-FRU and D-GLC were commonly observed from all three types of fruits but D-SOR and 3-CQA were only detected in prunes. It has been reported that D-SOR intolerance is common in humans and can often cause diarrhea^52^. Therefore, the prune is a natural laxative which can be used to treat constipation. 3-CQA on the other hand was reported to have antioxidant and anti-inflammatory effects^53^. Therefore, proper consumption of prunes can prevent chronic inflammation. Among these three types of fruits, L-TA is only detected in grapes. It has been reported that the tartaric acid not only enhances the astringency of wine^54^, but also affects its rheological properties and color^55^. Thus, accurate sensing of L-TA by the nanopore may provide a rapid but accurate way to grade grapes in the wine industry. On the other hand, CA and its isomer ICIT were simultaneously detected in lemons, demonstrating the exceptional resolution of MspA in the discrimination of isomers from a complex sensing environment. Lemon is widely applied in the seasoning of food and drinks in daily life and these two acids make a strong contribution to the generation of the unique sour taste of lemon^56,57^.

Though the use of the mini food processor and the centrifuge guarantees the efficiency and the reproducibility of juice generation, these operations are optional. To further simplify the workflow, the fruit can be manually squeezed and the generated juice can be directly analyzed by nanopore. To demonstrate this, manually squeezed lemon juice was ultrafiltered (3 kDa MWCO) at 3380 g for 3 min at 4 °C. The collected lemon juice was directly added to the nanopore sensor (Supplementary Fig. 37a, Methods). The whole operation took only 8 min and the measurement results (Supplementary Fig. 37b) are generally consistent with those demonstrated in Fig. 5f.

By taking grape juice as a demonstrative sample, the stability and consistency of this technique was also evaluated in a time-extended measurement (Supplementary Fig. 38). According to this demonstration, the nanopore assay could be continuously run for a few hours and a high consistency of results is shown. The characteristic event features acquired at the beginning of the measurements and a few hours later also appear completely identical (Supplementary Figs 38, 39). Though the MspA nanopore is protein in nature, according to previous investigations^58–60^, it demonstrates a high rigidity and durability in the structure, which is ideal for long term measurement and device storage.

### Rapid analysis of different varieties of kiwifruits

**Fig. 6:**
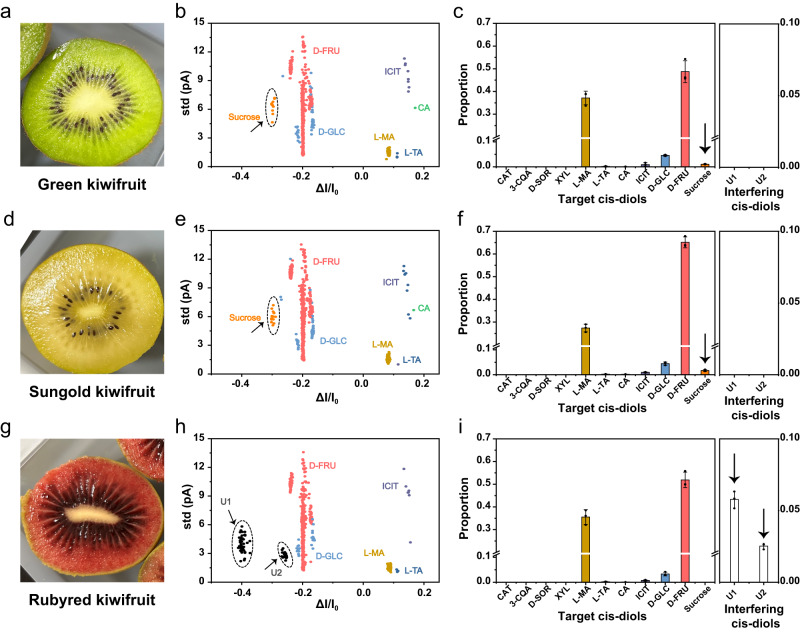
Rapid analysis of different varieties of kiwifruits. **a**, **d**, **g** Three commercially available kiwifruit varieties, including (**a**) Green, (**d**) Sungold and (**g**) Rubyred kiwifruit. The kiwifruit juice was generated as demonstrated in Methods. The nanopore measurements were performed using MspA-PBA in a 1.5 M KCl buffer with a continually applied bias of +160 mV. For each type of kiwifruit, 2 μL kiwifruit juice was added to both measurement chambers for nanopore sensing. **b**, **e**, **h** The scatter plots of *ΔI/I*_*0*_ versus std of events acquired with the juices of (**b**) Green kiwifruit (*n* = 930), (**e**) Sungold kiwifruit (*n* = 903) and (**h**) Rubyred kiwifruit (*n* = 950). These events were respectively acquired from 60 min continuous recordings for each condition. All target *cis*-diols except the sucrose were predicted by the Bagging Trees model. Sucrose events (marked as orange) were observed only in events acquired with green and sungold kiwifruits (Supplementary Figs 46–47, 49–51). Two extra populations of events, which don’t belong to any previously identified event type, were detected from events acquired with the Rubyred kiwifruit. These events were respectively marked as U1 (unidentified *cis*-diol 1) and U2 (unidentified *cis*-diol 2) (Supplementary Fig. 48) and colored black in (**h**). **c**, **f**, **i** The proportion of target *cis*-diols and interfering *cis*-diols events from results acquired with (**c**) Green kiwifruit, (**f**) Sungold kiwifruit and (**i**) Rubyred kiwifruit. The proportion of sucrose, U1 and U2 events from kiwifruits were also indicated with arrows. Data in the bar plots were presented as mean ± standard deviation values derived from results of three independent measurements (*N* = 3). The error bars represent standard deviation values. Source data are provided as a Source Data file.

The capacity of MspA-PBA to discriminate between a large variety of *cis*-diols also suggests its use to distinguish between fruits of different varieties. To demonstrate that, three varieties of commercially available kiwifruits from Zespri^TM^ (New Zeeland), including Green, Sungold and Rubyred kiwifruit (Fig. 6a, d, g), were analyzed by nanopore. The juice generation was carried out as described in Methods. The measurement was performed using MspA-PBA in a 1.5 M KCl buffer and a + 160 mV bias was continually applied. To initiate the measurement, 2 μL kiwifruit juice was respectively added to both *cis* and *trans* chambers. Immediately afterwards, corresponding events were observed (Supplementary Figs 40–42). All raw data acquired from different trials of measurements were shown in Supplementary Figs 43–45. The raw data were further treated by outlier analysis using One-Class SVM (Supplementary Figs 46–48), which is an unsupervised machine learning algorithm to detect outlier events. Details of One-Class SVM model establishment is summarized in Methods. After outlier analysis, all data were grouped into inlier and outlier events. All inlier events were predicted using the previously trained Bagging Trees model. Whereas, all outlier events were further treated by cluster analysis using DBSCAN to detect the appearance of newly appearing event clusters.

Specifically, new clusters of events, which don’t belong to any standard events recorded in the machine learning database, were detected in Sungold kiwifruit (Supplementary Fig. 47) and Rubyred kiwifruit (Supplementary Fig. 48), suggesting that previously uninvestigated *cis*-diols were detected in these kiwifruit varieties. According to that reported in literatures^61,62^, sucrose, which is a *cis*-diol not previously investigated by MspA-PBA, is also present in Sungold kiwifruit. It is thus speculated that the newly detected event cluster in Sungold kiwifruit might be from sucrose. To verify this hypothesis, sucrose was separately measured using MspA-PBA and its event features were recorded and confirmed to be consistent with that detected in Sungold kiwifruit (Supplementary Fig. 49). 500 standard events of sucrose were collected as a training dataset and fed to a One-Class SVM algorithm. This trained algorithm was further applied in the analysis of the outlier events acquired with the Green kiwifruit sample, by which few sucrose events were recognized according to the consistency of event features (Supplementary Fig. 50). The outlier analysis for sucrose detected in the Green kiwifruit was also demonstrated in Supplementary Fig. 51. By doing that, we can confirm that sucrose was detected in both Green and Sungold kiwifruit. The events of sucrose are more clearly seen in the Sungold kiwifruit likely because a higher concentration of sucrose is present in Sungold kiwifruit. However, events of sucrose were not detected in Rubyred kiwifruit, even assisted by searching using machine learning. However, two extra clusters of events were detected in Rubyred kiwifruit. Though these two event types were inconsistent with any previously identified *cis*-diols using MspA-PBA, they are treated as characteristic event types specifically detected in this kiwifruit variety. For the ease of demonstration, they were respectively marked as U1 (unidentified *cis*-diol 1) and U2 (unidentified *cis*-diol 2).

To summarize, *cis*-diols identified in Green kiwifruit and Sungold kiwifruit were confirmed to be sucrose, D-FRU, D-GLC, L-MA, L-TA, CA and ICIT (Fig. 6b, c, e, f). The results are also consistent with that previously studied using conventional methods^61,63^. For Rubyred kiwifruit, events of D-FRU, D-GLC, L-MA, L-TA, ICIT, U1 and U2 were identified (Fig. 6h, i). Three independent trails were also carried out with each kiwifruit variety to show the consistency of measurement (Supplementary Figs 52–54). According to above results, events of D-FRU, D-GLC, L-MA, L-TA and ICIT were detected from all kiwifruit varieties. Sucrose was only detected in Green and Sungold kiwifruit and the Sungold kiwifruit reports a higher concentration of sucrose than that measured with the Green kiwifruit and sucrose was not detected in Rubyred kiwifruit. Instead, two previously unidentified *cis*-diols were detected only in Rubyred kiwifruit. These different combinations of *cis*-diols in different kiwifruit varieties should contribute to the different taste of these kiwifruit products. However, to the best of our knowledge, a direct single molecule discrimination between molecular compounds in different fruit varieties has never been previously reported.

### Rapid analysis of commercial fruit juice products

**Fig. 7:**
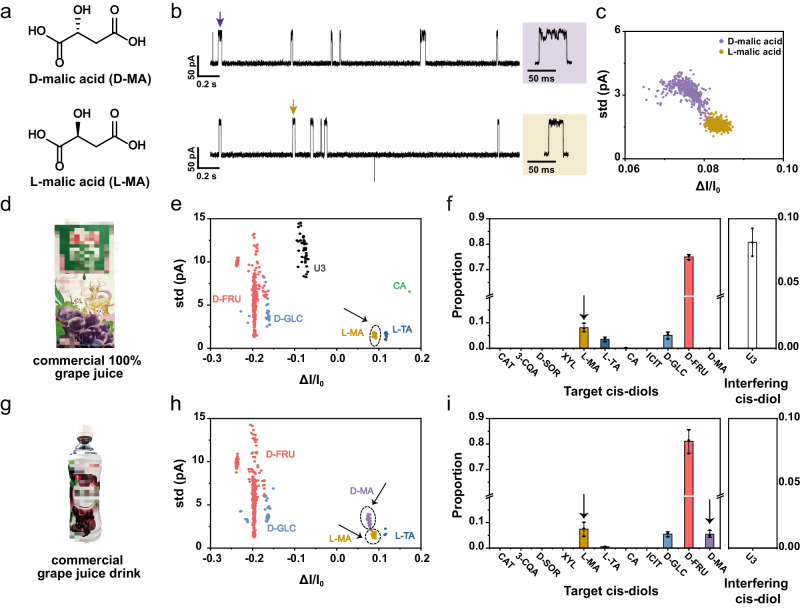
Rapid analysis of commercial juice and juice drink. **a** The chemical structures of D-malic acid (D-MA) and L-malic acid (L-MA). **b** Representative traces respectively acquired with D-MA (lavender) and L-MA (claybank). The corresponding representative events were demonstrated to the right of the traces. **c** The scatter plot of *ΔI/I*_*0*_ versus std of events acquired from D-MA and L-MA. 500 events from each type of analytes were included in the scatter plot (*n* = 1000). The event features of D-MA were also added to the previous training set to generate new machine learning models. The Bagging Trees still showed the highest accuracy, measuring 98.9% (Supplementary Fig. 57). **d**, **g** Two types of commercial juice and juice drink, including (**d**) 100% grape juice (Huiyuan^TM^) and (**g**) grape juice drink (Wahaha^TM^). **e**, **h** The scatter plot of *ΔI/I*_*0*_ versus std of events acquired with (**e**) 100% grape juice (*n* = 477) and (**h**) grape juice drink (*n* = 555). Events demonstrated were respectively acquired from 60 min continuous recording for each condition. The target *cis*-diols in 100% grape juice and grape juice drink were first treated with outlier analysis (Supplementary Figs 62, 63) and then predicted by the Bagging Trees model (Supplementary Fig. 57). L-MA in 100% grape juice and DL-MA in grape juice drink were identified using machine learning and indicated with arrows and the circles. A population of events in 100% grape juice, which doesn’t belong to any previously identified event type, was also detected and grouped by a DBSCAN algorithm and marked as U3 (unidentified *cis*-diol 3) (Supplementary Fig. 62). **f**, **i** The proportion of target *cis*-diols and interfering *cis*-diols events from results acquired with (**f**) 100% grape juice and (**i**) grape juice drink. Data in the bar plots were presented as mean ± standard deviation values derived from results of three independent measurements (*N* = 3). The error bars represent standard deviation values. The proportion of DL-MA events from two samples were also indicated with arrows. All results were acquired by nanopore measurements using MspA-PBA in a 1.5 M KCl buffer with a continually applied bias of +160 mV. Two commercial juice and juice drink products were directly added to both chambers without any sample treatment. Source data are provided as a Source Data file.

Following the same principle, *cis*-diols may as well be identified directly from commercial fruit juice products. Specifically, L-malic acid (L-MA), which is a naturally occurring α-hydroxy acid, is widely found in fruits^64^. Its enantiomer, D-malic acid (D-MA), is however rarely seen in nature. Due to the cost reasons, a mixture of DL-malic acid (Fig. 7a) instead of L-malic acid, is more frequently applied as synthetic food additive^65,66^. Another pair of similar α-hydroxy acid enantiomers used as food additive is DL-tartaric acid^67^. However, rapid discrimination of α-hydroxy acid enantiomers directly from a food sample is not a trivial task.

To testify this using nanopore, MspA-PBA sensing of D-MA and L-MA was respectively carried out in a 1.5 M KCl buffer with a + 160 mV bias. Representative traces were demonstrated in Fig. 7b, according to which the events of D-MA and L-MA are fully distinguishable. This difference is more clearly shown in the corresponding event scatter plot (Fig. 7c, Supplementary Figs 8, 55), further confirming the high resolution of MspA-PBA in the discrimination between α-hydroxy acid enantiomers. To enable machine learning based identification, 500 events acquired with D-MA were collected and added to the existing training set already containing ten *cis*-diols (Supplementary Fig. 56). By doing that, a new machine learning model, which can identify 11 *cis*-diols including both D-MA and L-MA, is established. According to the training and validation with a variety of models, the Bagging Trees model has performed the best by reporting an accuracy of 98.9% (Supplementary Fig. 57). This model is thus used for all further event predictions.

Nanopore sensing was then respectively carried out with commercial 100% grape juice (Huiyuan^TM^, Fig. 7d) and grape juice drink (Wahaha^TM^, Fig. 7g). The measurements were performed with MspA-PBA in a 1.5 M KCl buffer and a + 160 mV continually applied bias. Without performing any sample separation, 2 μL different types of juice sample was respectively added to the nanopore device in separate measurements. Immediately afterwards, corresponding nanopore events were observed with different commercial juice products (Supplementary Figs 58, 59). Three independent trails were also carried out for each condition to show its reproducibility (Supplementary Figs 60–61).

All acquired nanopore events were first treated by outlier analysis (Supplementary Figs 62, 63). By using One-Class SVM, an unsupervised machine learning algorithm for outlier detection, all events that fail to show any consistency with the standard events generated by the previously investigated 11 *cis*-diols, were grouped as outlier events. All outlier events were further treated by cluster analysis to check the presence of any cluster-forming events. Specifically, for the events acquired with the 100% grape juice, a cluster of events which has never been previously investigated using MspA-PBA, was discovered. For the ease of demonstration, this cluster of events is referred to as U3 (unidentified *cis*-diol 3). The U3 events were however never detected at all in any freshly squeezed grape juice samples mentioned in this paper. It is speculated that the U3 event might be from the specific variety of grape used in the generation of the commercial 100% grape juice or it might be from food additives introduced during juice production.

For all inlier events, the newly established Bagging Trees model was applied for event identification. For both types of commercial juice products, events of D-FRU, D-GLC, L-MA and L-TA were clearly identified (Fig. 7e–f, h–i). Three independent trails were also carried out with each commercial juice product to show the consistency of measurement (Supplementary Figs 64–65). In the 100% grape juice, only L-MA was detected, suggesting that no DL-MA food additive was added to this juice product. However, both D-MA and L-MA were detected in the grape juice drink, consistent with that listed in the corresponding ingredient table. With the established machine learning algorithm, all above fruit juice analysis was carried out extremely rapidly. Both juice samples were added directly to the nanopore device without any need of sample separation.

**Fig. 8:**
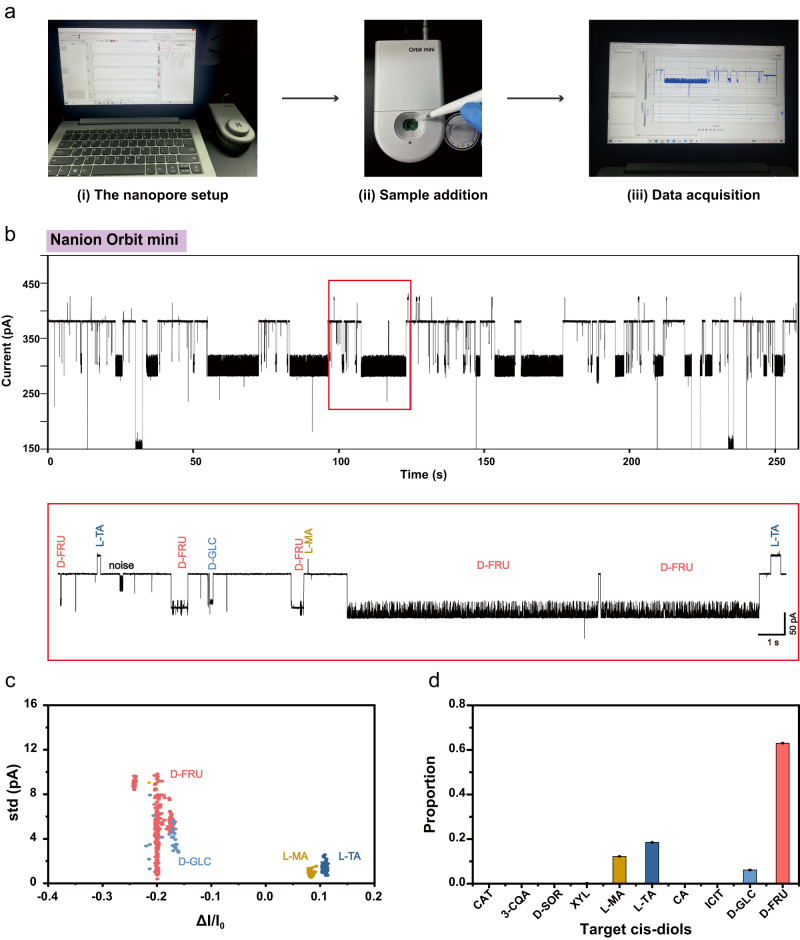
Portable nanopore analysis of grape juice. **a** The workflow of portable nanopore analysis of grape juice. The grape juice was prepared as described in Methods. (i) The nanopore setup. A Nanion Orbit mini was paired with a laptop computer to perform the measurements. (ii) Sample addition. 1 μL grape juice was added to the chip and stirred thoroughly to initiate the measurement. The nanopore measurement was performed using MspA-PBA in a 1.5 M KCl buffer and a continually applied bias of +160 mV. (iii) Data acquisition. Nanopore events of grape juice were observed immediately after sample addition. **b** A representative trace acquired with natural grape juice using Nanion Orbit mini. 1 μL grape juice was added to the measurement device. The top figure is a 260 s continuous trace. A section of this trace, as marked with a red box, is zoomed in and shown below. Events of L-MA, L-TA, D-GLC and D-FRU were identified and labelled on the trace using machine learning (Fig. 3). Noise events were also detected and marked. **c** Scatter plot of *ΔI/I*_*0*_ versus std for nanopore events acquired with grape juice (*n* = 492). All background events were removed with DBSCAN cluster analysis (Supplementary Fig. 66). All events were predicted by the previously trained Bagging Trees model (Fig. 3). The data presented in each scatter plot was from a continuous measurement of 40 min. **d** The proportion of target *cis*-diols events in grape juice acquired using Nanion Orbit mini. Data presented in the bar plot were from one measurement (*N* = 1). The results are consistent with that acquired with the Axon 200B+digidata 1550B device (Supplementary Figs 31, 35). Source data are provided as a Source Data file.

To also show the portability of the technique, nanopore analysis of fruits was also carried out with an Orbit mini portable patch clamp amplifier (Nanion Technologies GmbH) (Fig. 8). To perform the measurement, the Orbit mini, which is of a palm size and an extremely light weight, is connected to a routine laptop computer, weighing ~1 kg. The operation of the whole setup can be powered solely by the inbuilt laptop battery, enabling the application of nanopore sensing even without any electricity supply. Representative traces and statistical results produced with natural grape juice at this condition are summarized in Fig. 8. The results are generally consistent with that acquired using the Axon200B patch clamp amplifier and Digidata 1550B digitizer.

Compared with conventional analytical methods such as mass spectrometry, liquid chromatography and gas chromatography, a nanopore platform generally offers a higher portability, a lower measurement cost, a more simplified sample preparation and the capacity to perform simultaneous sensing of multiple analytes, which is extremely suitable for portable food analysis. This might be useful for food industry or food and drug administration. Specifically, its high resolution in direct discrimination between isomers and enantiomers is advantageous over MS. It also offers a more rapid result feedback, which is generally faster than chromatography-based methods. Being an emerging method, the nanopore platform at its present form is still disadvantageous in the limit of detection and the accuracy of quantification. We however foresee that these disadvantages could be improved in future development of this technique.

## Discussion

MspA-PBA, a reactive MspA nanopore containing a sole PBA adapter, was applied to the identification in fruit juice of catechin, neochlorogenic acid, D-sorbitol, xylitol, DL-malic acid, L-tartaric acid, citric acid, (2*R*,3*S*)-isocitric acid, sucrose, D-glucose and D-fructose. These 12 analytes respectively represent saccharides, alditols, 1,2-diphenols and α-hydroxy acids widely encountered in fruits. Though nucleotides are in principle detectable by MspA-PBA^42,44^, their concentrations are too low to be detected directly from the fruit juice. In each measurement, only ~ µL fruit juice was added, which doesn’t significantly change the pH and ion strength of the buffer environment (Supplementary Table 6). The inherent proteins, nucleic acids and polysaccharides in fruits, which either don’t interact with the PBA or report clearly distinguishable events, are also not interfering with the measurements. By simultaneously considering five event features, including *ΔI/I*_*0*_, std, kurt, skew and time, a custom machine learning algorithm provides automatic and unbiased identification of unknown nanopore events with a classification accuracy of 99.3%. A nanopore that can simultaneously detect saccharides, alditols, 1,2-diphenols and α-hydroxy acids has not been demonstrated previously. Although the previous studies have reported the use of phenylboronic acid-modified nanopores for saccharide sensing, they either fail to achieve a single molecule resolution and are incapable to perform simultaneous sensing^68,69^ or report a clearly insufficient resolution^70^ for fruit analysis. This sensing capacity of MspA-PBA was further applied to the direct and rapid analysis of natural fruit juice of prune, grape, lemon and different varieties of kiwifruits. The capacity of MspA-PBA to discriminate between DL-malic acid also enables its immediate application in the detection of synthetic food additive in commercial juice products. To the best of our knowledge, nanopore sensing of catechin, neochlorogenic acid, DL-malic acid, L-tartaric acid, citric acid, (2*R*,3*S*)-isocitric acid and sucrose has not been previously reported. Nanopore analysis of natural fruit juice, which systematically demonstrates how natural complex samples can be analyzed in single molecule, was also not previously reported. Though the technique at its current form can only detect free *cis*-diols in natural samples, it is possible to release the bound *cis*-diol by sample treatment of enzymatic^71,72^ or chemical hydrolysis^71,73,74^, followed with nanopore analysis. Though only demonstrated with fruits, this sensing strategy is in principle generally suitable for other natural samples containing *cis*-diol components. All nanopore measurements can also be carried out in a miniaturized device (Fig. 8) to demonstrate portable food analysis. Different from that required in genome sequencing, the food samples normally contain abundant *cis*-diols and the need of a high-throughput acquisition system to maximize the efficiency of sensing is less urgent. However, a multiplexed array of sensors which can simultaneously analyze different food samples is more practically needed.

## Methods

### Nanopore preparations

The preparation of MspA-PBA^36,42–44^ was carried out as described below. Briefly, the desired MspA hetero-octamer, which was referred to as (N90C)_1_(M2)_7_, was composed of seven units of M2 MspA-D16H6 (D90N/D91N/D93N/D118R/D134R/E139K) and one unit of N90C MspA-H6 (D90C/D91N/D93N/D118R/D134R/E139K). The genes of M2 MspA-D16H6 and N90C MspA-H6 were respectively synthesized by GenScript. Both genes were simultaneously placed in the same co-expression vector pETDuet-1 and expressed in *E. coli* BL21 (DE3) pLysS competent cells (GenScript, New Jersey). After protein expression, the collected bacterial pellets were resuspended in the lysis buffer (100 mM Na_2_HPO_4_/NaH_2_PO_4_, 0.1 mM EDTA, 150 mM NaCl, 0.5% (v/v) Genapol X-80, pH 6.5) and incubated at 60 °C for 50 min. After being centrifuged and ice-incubated for 30 min, the supernatant in the suspension was collected, filtered and loaded to a HisTrap^TM^HP nickel ion affinity column (GE Healthcare). The column was first eluted with buffer A (0.5 M NaCl, 20 mM HEPES, 5 mM imidazole, 0.5% (w/v) Genapol X-80, pH 8.0) to elute miscellaneous proteins. It was then eluted with a linear gradient of mixing buffer A and buffer B (0.5 M NaCl, 20 mM HEPES, 500 mM imidazole, 0.5% (w/v) Genapol X-80, pH 8.0) to collect all hetero-octameric assemblies of MspAs. To separate (N90C)_1_(M2)_7_ from all other hetero-octameric assemblies, a 10% SDS- polyacrylamide gel was used to perform gel electrophoresis of the eluent fractions collected from the nickel column. After gel electrophoresis, the gel was stained with coomassie brilliant blue (1.25 g coomassie brilliant blue R250, 225 mL methanol, 50 mL glacial acetic acid, 225 mL ultrapure water) for 4 h and de-stained with an elution buffer (40% (v/v) MeOH, 10% (v/v) glacial AcOH). The gel fragment containing the target (N90C)_1_(M2)_7_ assembly was then excised from the gel. The obtained gel piece was crushed and immersed in a protein extraction buffer (150 mM NaCl, 15 mM Tris-HCl, 0.2% (w/v) DDM, 0.5% (v/v) Genapol X-80, 5 mM TCEP, 10 mM EDTA, pH 7.5). The extracted (N90C)_1_(M2)_7_ was immediately used or stored at −80 °C for long term use.

To modify (N90C)_1_(M2)_7_ with phenylboronic acid, 2 μL 3-(maleimide) phenylboronic acid (MPBA, 500 mM) and 5 μL freshly prepared (N90C)_1_(M2)_7_ were simultaneously added to a 43 μL 1.5 M KCl buffer (1.5 M KCl, 100 mM MOPS, pH 7.0) and thoroughly mixed. The mixture was incubated at 25 °C for 10 min to finalize pore modification. The prepared nanopore was either immediately used in all downstream nanopore measurements or is stored at −80 °C for long term use. For simplicity, this MPBA modified (N90C)_1_(M2)_7_ is referred to as MspA-PBA throughout the manuscript.

### Nanopore measurements

Nanopore measurements were performed similarly to that reported previously^42^. All nanopore measurements were performed with an Axonpatch 200B patch-clamp amplifier paired with a Digidata 1550B digitizer (Molecular Devices). To reduce external environment noises, the custom measurement device was fixed in a custom Faraday cage mounted on an optical table (Jiangxi Liansheng technology Co., Ltd). The measurement device was composed of two chambers separated by a Teflon film containing an aperture of about 100 μm in diameter. Prior to the measurement, the aperture was first treated with 2% (v/v) hexadecane in pentane and set for pentane evaporation. Then both chambers were filled with 500 μL 1.5 M KCl buffer (1.5 M KCl, 100 mM MOPS, pH 7.0). Afterwards, two Ag/AgCl electrodes which were separately connected to the patch-clamp amplifier, were respectively inserted in each chamber, in contact with the buffer solution. By convention, the electrically grounded chamber is defined as the *cis* chamber and its opposing chamber is defined as the *trans* chamber. To form a phospholipid bilayer on the aperture of the Teflon film, 100 µL pentane solution of DPhPC (5 mg/mL) was added to both chambers and the lipid bilayer spontaneously forms when the buffer solution in one of the chambers has been pipetted up and down for several times. Spontaneous pore insertion was triggered by adding newly prepared MspA-PBA to the *cis* chamber. Upon a single nanopore insertion, the buffer in the *cis* chamber was immediately exchanged with fresh buffer to avoid further pore insertions.

All nanopore measurements were performed with a single MspA-PBA channel and all analytes were added to both chambers. Specifically, the portable nanopore analysis of natural grape juice analysis was carried out using Nanion Orbit Mini (Fig. 8). Unless otherwise stated, all other measurements were performed using Axonpatch 200B patch-clamp amplifier paired with a Digidata 1550B digitizer (Molecular Devices). Unless otherwise stated, all measurements were performed at 25 degrees of Celsius with a continually applied bias of +160 mV. All single-channel recordings were sampled at 25 kHz and low-pass filtered with a corner frequency of 1 kHz. Unless otherwise stated, the final concentrations of the analytes in both chambers were set as: 0.8 mM for catechin (CAT), 0.5 mM for neochlorogenic acid (3-CQA), 2 mM for D-sorbitol (D-SOR), 2 mM for xylitol (XYL), 0.6 mM for L-malic acid (L-MA) and D-malic acid (D-MA), 0.6 mM for L-tartaric acid (L-TA), 6 mM for citric acid (CA), 1 mM for isocitric acid (ICIT), 30 mM for D-glucose (D-GLC) and 10 mM for D-fructose (D-FRU). Based on the principle of nanopore sensing by single molecule reaction, the event feature is independent of the analyte concentration. With only a sole reactive adapter at the pore constriction, simultaneous capturing of more than one analyte is also technically impossible. However, some of the analyte type may require a higher analyte concentration to report a sufficiently high event appearance rate. Thus, the selection of these analyte concentrations is to maximize the efficiency of nanopore appearance rate.

### Data analysis

Event detection was first performed by the “single channel search” function of Clampfit 10.7. Subsequently, the start time, end time and dwell time of each event were obtained. Only events with a dwell time more than 10 ms were used for further analysis. The start and end times of events were listed in a text file for subsequent event feature extraction. The raw Axon abf files and the text file containing the start and end times of the events in the files were imported into MATLAB. Based on the start time and the end time of the event, the event features including blockage ratio (*ΔI/I*_*0*_), standard deviation (std), kurtosis (kurt), skewness (skew) and dwell time (time) were extracted for machine learning. The generation of histograms and scatter plots were performed by Origin 9.2 (Origin Lab).

Machine learning was performed by the “classification learner” toolbox in MATLAB. For CAT, 3-CQA, D-SOR, XYL, L-MA, L-TA, ICIT, 500 and 100 events of each analyte were collected to form the training set and the testing set, respectively. For D-GLC and D-FRU, 1000 and 200 events of each analyte were collected to form the training and the testing set. For CA, 100 and 20 events were collected to form the training and the testing set. The label of each event was assigned as the identification of the chemical compound used in the production of the data. Model training was performed with the training set and the 10-fold cross validation accuracies were obtained. The testing set was used to test all trained models and testing accuracies were reported. Different models, including Decision Trees, Discriminant Analysis, Naïve Bayes, Support Vector Machine (SVM), K Nearest Neighbor (KNN), Ensemble and Neural Network were trained and tested. According to the results of 10-fold cross validation accuracy and the testing accuracy (Supplementary Table 3), the Bagging Trees model reported the highest overall accuracy. Therefore, the trained Bagging Trees model was used for further event prediction.

The density-based spatial clustering of applications with noise (DBSCAN) cluster analysis was applied for clustering on a Python platform. The Epsilon was set to 0.17 and the min_samples was set to 18 for analysis of prune juice, grape juice and lemon juice. The Epsilon was set to 0.1 and the min_samples was set to 10 for analysis of kiwifruit. The Epsilon was set to 0.3 and the min_samples was set to 30 for analysis of commercial 100% grape juice.

One-Class SVM, a machine learning algorithm for outlier analysis, was performed by the ‘Scikit-learn library’ in Python. The training set for outlier analysis of kiwifruits is the same as the ten *cis*-diols training set described in Fig. 3. The training set for outlier analysis of commercial products is the same as the eleven *cis*-diols training set described in Supplementary Fig. 57. The training set of sucrose, which contains 500 standard events was acquired with sucrose. The data was later used in the identification of sucrose in Sungold kiwifruit and Green kiwifruit. Briefly, *ΔI/I*_*0*_, std, kurt, skew and time of events were employed as event features for outlier analysis. The parameter ‘nu’ was set to 0.05. Different One-Class SVM models were respectively trained by different training set for further event discrimination. The model automatically judges whether the analyzed event belongs to any previously trained event types so that the event is considered as an inlier event. If not, it is considered as an outlier event instead. The inlier events of kiwifruits and commercial products were further predicted by the trained Bagging Trees models, as respectively described in Fig. 3 and Supplementary Fig. 57. The outlier events were then treated with cluster analysis using DBSCAN. If a group of these outlier events were identified to have similar event features and appear as a cluster, this group of outlier events are considered a set of detected but unidentified *cis*-diol.

All machine learning models and sample data used to train, validate and test the model have been deposited in Figshare. Please follow the link https://figshare.com/s/5fc838dad55e6452bc95 for download.

### Fruit juice preparations

Technical details of all fruit samples used in the measurements were demonstrated in Supplementary Fig. 67. To perform the measurement, different fruits were respectively washed, cut into pieces and mashed into pulp. Then, the pulp of different fruits was separately treated by a mini food processor to produce puree (Figs 4a, 5a, 6). To remove the solid residues, the puree was centrifuged at 1900 g for 10 min at 4 °C and the supernatant was collected as juice. The supernatant was then added into an ultrafiltration tube with a 3 kDa molecular weight cut off (MWCO) and centrifuged at 2350 g for 10 min at 4 °C. The filtrate was collected and immediately used in all downstream measurements. The whole operation takes about 25 min (Fig. 4a).

In Supplementary Fig. 37, the lemons were cut into fruit pieces. The fruit pieces were pinched by hand to product fruit juice. And the fruit juice was added into the ultrafiltration tube with a 3 kDa molecular weight cut off (MWCO) and centrifuged at 3380 g for 3 min at 4 °C. The filtrate was collected and immediately used in all downstream measurements. The whole operation takes about 8 min (Supplementary Fig. 37a).

### Quantification of target *cis*-diols

The concentrations of target *cis*-diols were evaluated according to the following equation:

1 $${C}_{i}={E}_{i}/\left({k}_{i}*t\right)$$

Where $i$ (from 1 to 10) stands for different *cis*-diols, including CAT, 3-CQA, D-SOR, XYL, L-MA, L-TA, CA, ICIT, D-GLC and D-FRU, respectively. ${E}_{i}$ is the number of binding events of each *cis*-diol type. ${k}_{i}$ is the calibration coefficient defined as the number of event occurrences per unit concentration (mM) per unit time (ms). The value of ${k}_{i}$ is derived from results of the concentration dependence measurements (Supplementary Figs 14–23). The results were also summarized in Supplementary Tables 4, 5. $t$ is the recording time of the measurement.

### Reporting summary

Further information on research design is available in the Nature Portfolio Reporting Summary linked to this article.

### Supplementary information

Supplementary Information

Peer Review File

Description of additional supplementary files

Supplementary Movie 1

Supplementary Movie 2

Supplementary Movie 3

Reporting Summary

### Source data

Source data

## Data Availability

Data supporting the findings of this study are shown in the main text and the Supplementary Information, which are also available within the source data provided with this paper. All data used to train, evaluate and test the machine learning model have been deposited in Figshare. Please follow the link: https://figshare.com/s/5fc838dad55e6452bc95 for download. Source data are provided with this paper.

## Code availability

The custom machine learning code and sample data used to train, validate and test the model have been deposited in Figshare. For simplicity, the shared package is referred to as ‘fruit classifier’. Please follow the link https://figshare.com/s/5fc838dad55e6452bc95 for download.
