# Supplementary material for: Nanopore analysis of cis-diols in fruits
Source: Nat Commun. 2024 Mar 5;15:1969. doi: 10.1038/s41467-024-46303-x (PMC10915164; doi:10.1038/s41467-024-46303-x)
Supplement: Supplementary file 1 — Supplementary Information [file 41467_2024_46303_MOESM1_ESM.pdf]

Supplementary Information

Nanopore analysis of *cis*-diols in fruits

Pingping Fan[ab#], Zhenyuan Cao[ab#], Shanyu Zhang[ab#], Yuqin Wang [abcd], Yunqi Xiao[ab], Wendong Jia[ab], Panke Zhang[a] and Shuo Huang[*ab]

[a]State Key Laboratory of Analytical Chemistry for Life Sciences, School of Chemistry and Chemical Engineering, Nanjing University, 210023, Nanjing, China.

[b]Chemistry and Biomedicine Innovation Center (ChemBIC), Nanjing University, 210023, Nanjing, China.

[c.] State Key Laboratory of Pollution Control and Resource Reuse, School of the Environment, Nanjing University, 210023, Nanjing, China.

[d.] Institute for the Environment and Health, Nanjing University Suzhou Campus, 215163, Suzhou, China

[#]These authors contributed equally to this work.

*Corresponding Author. Email: shuo.huang@nju.edu.cn

Materials

1,2-diphytanoyl-*sn*-glycero-3-phosphocholine (DPhPC) was obtained from Avanti Polar Lipids. Pentane, hexadecane, tris(2-carboxyethyl) phosphine hydrochloride (TCEP), ethylenediamine-tetraAcOH (EDTA), Genapol X-80, ammonium persulfate (≥98.0%) (APS), sodium dodecyl sulfate (≥98.5%) (SDS), N,N,N′,N′-tetramethylethylenediamine (99.0%) (TEMED), 30% acrylamide/bis-acrylamide solution and sodium acetate (CH_3COONa) were from Sigma-Aldrich. Potassium chloride (KCl), sodium chloride (99.99%) (NaCl), sodium hydroxide (99.9%) (NaOH), sodium hydrogen phosphate (Na_2HPO_4), sodium dihydrogen phosphate (NaH_2PO_4), 3-morpholine propionic acid (MOPS), methanol, glacial acetic acid, n-dodecyl-β-D-maltoside (DDM), coomassie brilliant blue R250, D-malic acid (D-MA, 99%) and D-sorbitol (D-SOR, 98%) were from Aladdin (China). Hydrochloric acid (HCl) was from Sinopharm (China). Xylitol (XYL, 98%) was from Shanghai Yuanye Bio-Technology (China). Dioxane-free isopropyl-β-D-thiogalactopyranoside (IPTG), kanamycin sulfate, ampicillin, imidazole and tris (hydroxymethyl)aminomethane (Tris) were from Solarbio. Precision Plus Protein™ Dual color Standards, TGX™ FastCast™ Acylamide Kit (4-15%), stacking gel buffer (0.5M Tris-HCl buffer, pH 6.8) and resolving gel buffer (1.5M Tris-HCl buffer, pH 8.8) were from Bio-rad. SDS-PAGE electrophoresis buffer powder was from Beyotime (China). LB broth and LB agar were from Hopebio (China). *E. coli* BL21(DE3) pLysS competent cells and chloramphenicol were from Sangon Biotech. Isocitric acid (ICIT, 97%), neochlorogenic acid (3-CQA, 99%) and sucrose (≥99.5%) were from Macklin (China). 3-(maleimide) phenylboronic acid (MPBA, Cat. #sc-352346) was from Santa Cruz Biotechnology (Shanghai) Co., Ltd. L-malic acid (L-MA, 98%), tartaric acid (L-TA, 98%), citric acid (CA, 98%) and catechin (CAT, 97%) were from Bide Pharmathch Co., Ltd (China). D-fructose (D-FRU, ≥98%) was from Shanghai Dibai Bio-Technology (China). D-glucose (D-GLC, 99%) was from Damas-beta (China). All the items listed above were used as received.

1.5 M KCl buffer (1.5 M KCl, 100 mM MOPS, pH 7.0), lysis buffer (100 mM Na_2HPO_4/NaH_2PO_4, 0.1 mM EDTA, 150 mM NaCl, 0.5% (v/v) Genapol X-80, pH 6.5), buffer A (0.5 M NaCl, 20 mM HEPES, 5 mM imidazole, 0.5% (w/v) Genapol X-80, pH 8.0), buffer B (0.5 M NaCl, 20 mM HEPES, 500 mM imidazole, 0.5% (w/v) Genapol X-80, pH 8.0), protein extraction buffer (150 mM NaCl, 15 mM Tris-HCl, 0.2% (w/v) DDM, 0.5% (v/v) Genapol X-80, 5 mM TCEP, 10 mM EDTA, pH 7.5) and elution buffer (40% (v/v) MeOH, 10% (v/v) glacial AcOH) were prepared with Milli-Q water and membrane (0.2 μm, Whatman) filtered. All fruits and commercial fruit juice or drink were purchased from local super market.

Supplementary Table 1. The average $\overline{1/\tau_{on}}$ and $\overline{1/\tau_{off}}$ of ten target *cis*-diols measured at various concentrations. All measurements were performed as described in **Methods**. Ten target *cis*-diols were respectively added to *cis* and *trans* chambers with the desired concentrations. The mean of reciprocal of inter-event interval ($\overline{1/\tau_{on}}$) and mean dwell time $\overline{1/\tau_{off}}$ were derived from single-exponential fitting, according to the equation $y = a * \exp(-t/\tau)$. All statistical results were derived from results of three independent measurements (N=3).

Cis-diols	Concentration(mM)	$\overline{1/\tau_{off}}$ (ms^{-1})	$\overline{1/\tau_{on}}$ (ms^{-1})
CAT	0.2	(1.3±0.3) *10^{-3}	(0.97±0.04) *10^{-3}
	0.4	(1.37±0.12) *10^{-3}	(2.34±0.09) *10^{-3}
	0.6	(1.5±0.4) *10^{-3}	(3.8±0.3) *10^{-3}
	0.8	(1.4±0.2) *10^{-3}	(5.10±0.08) *10^{-3}
	1	(1.22±0.16) *10^{-3}	(6.2±0.7) *10^{-3}
3-CQA	0.1	(1.28±0.13) *10^{-3}	(2.1±0.8) *10^{-3}
	0.2	(1.24±0.16) *10^{-3}	(3.9±0.8) *10^{-3}
	0.3	(1.16±0.10) *10^{-3}	(5.0±1.1) *10^{-3}
	0.4	(1.43±0.09) *10^{-3}	(6.1±1.5) *10^{-3}
	0.5	(1.4±0.2) *10^{-3}	(7.8±0.2) *10^{-3}
D-SOR	1	(0.86±0.07) *10^{-3}	(0.37±0.05) *10^{-3}
	2	(0.88±0.11) *10^{-3}	(0.66±0.06) *10^{-3}
	3	(0.87±0.05) *10^{-3}	(1.1±0.2) *10^{-3}
	4	(0.88±0.18) *10^{-3}	(1.5±0.5) *10^{-3}
	5	(0.88±0.12) *10^{-3}	(2.0±0.4) *10^{-3}
XYL	1	(3.7±0.4) *10^{-3}	(0.44±0.05) *10^{-3}
	2	(3.5±0.5) *10^{-3}	(0.91±0.09) *10^{-3}
	3	(3.6±0.4) *10^{-3}	(1.5±0.2) *10^{-3}
	4	(3.8±0.4) *10^{-3}	(1.8±0.3) *10^{-3}
	5	(3.6±0.3) *10^{-3}	(2.3±0.4) *10^{-3}
L-MA	0.05	(66±6) *10^{-3}	(0.08±0.02) *10^{-3}
	0.1	(75±8) *10^{-3}	(0.14±0.02) *10^{-3}
	0.15	(74±3) *10^{-3}	(0.21±0.05) *10^{-3}
	0.2	(69±4) *10^{-3}	(0.29±0.05) *10^{-3}
	0.25	(82±6) *10^{-3}	(0.339±0.008) *10^{-3}
L-TA	0.3	(11.8±0.3) *10^{-3}	(0.16±0.04) *10^{-3}
	0.6	(12.0±0.4) *10^{-3}	(0.27±0.02) *10^{-3}
	0.9	(12.9±0.8) *10^{-3}	(0.43±0.09) *10^{-3}
	1.2	(12.7±1.0) *10^{-3}	(0.70±0.07) *10^{-3}
	1.5	(13.6±0.8) *10^{-3}	(0.95±0.09) *10^{-3}
CA	2	(0.036±0.013) *10^{-3}	(0.013±0.005) *10^{-3}
	4	(0.06±0.03) *10^{-3}	(0.04±0.02) *10^{-3}
	6	(0.076±0.009) *10^{-3}	(0.051±0.013) *10^{-3}
	8	(0.045±0.002) *10^{-3}	(0.060±0.017) *10^{-3}
	10	(0.055±0.006) *10^{-3}	(0.08±0.03) *10^{-3}

Cis-diols	Concentration(mM)	$\overline{1/\tau_{off}}$ (ms^{-1})	$\overline{1/\tau_{on}}$ (ms^{-1})
ICIT	0.2	(30.7±1.7) *10^{-3}	(0.33±0.07) *10^{-3}
	0.4	(32±2) *10^{-3}	(0.75±0.15) *10^{-3}
	0.6	(29.7±1.8) *10^{-3}	(1.12±0.11) *10^{-3}
	0.8	(28.1±1.9) *10^{-3}	(1.6±0.2) *10^{-3}
	1	(27.8±1.1) *10^{-3}	(2.04±0.10) *10^{-3}
D-GLC	6	(15±3) *10^{-2}	(0.13±0.03) *10^{-3}
	12	(14.4±1.5) *10^{-2}	(0.23±0.03) *10^{-3}
	18	(17±4) *10^{-2}	(0.36±0.04) *10^{-3}
	24	(15±2) *10^{-2}	(0.47±0.05) *10^{-3}
	30	(14±5) *10^{-2}	(0.5±0.7) *10^{-3}
D-FRU	1	(4.3±0.5) *10^{-3}	(0.49±0.13) *10^{-3}
	2	(4.5±0.5) *10^{-3}	(0.99±0.02) *10^{-3}
	3	(4.33±0.06) *10^{-3}	(1.54±0.07) *10^{-3}
	4	(3.93±0.03) *10^{-3}	(2.02±0.09) *10^{-3}
	5	(4.93±0.07) *10^{-3}	(2.45±0.09) *10^{-3}

Supplementary Table 2. The average Δ*I*/*I*$_0$, std, skew, kurt and time of ten *cis*-diols from three independent measurements. Five features of nanopore events were extracted to obtain an average value from each independent measurement separately (n = 500 for CAT, 3-CQA, D-SOR, XYL, L-MA, L-TA, ICIT, n = 100 for CA, n = 1000 for D-GLC and D-FRU in each pore). Each analyte was added to both chambers with a desired concentration (**Methods**). The nanopore measurements were performed in 1.5 M KCl buffer with a continuous bias of +160 mV. The average values of features obtained from three independent experiments for each *cis*-diol are reproducible.

Analyte		$\Delta I/I_0$	std (pA)	skew	kurt	time (ms)
**CAT**	Pore1	-0.2523	2.9214	0.2034	6.4005	690.6066
	Pore2	-0.2537	3.0665	0.3705	8.0389	711.8555
	Pore3	-0.2540	2.9934	0.3176	8.6059	696.5199
**3-CQA**	Pore1	-0.1172	3.7613	0.1319	7.3431	740.4987
	Pore2	-0.1198	3.7867	0.0100	5.4675	651.5676
	Pore3	-0.1213	3.7868	-0.0333	5.7466	705.9296
**D-SOR**	Pore1	-0.2563	8.0562	-1.0483	3.3160	1212.3656
	Pore2	-0.2516	7.9843	-1.0475	3.2124	1172.3828
	Pore3	-0.2535	8.1353	-1.0176	3.7647	1252.9103
**XYL**	Pore1	-0.2148	9.4630	0.0198	1.8049	243.2963
	Pore2	-0.2139	9.4532	0.0355	1.8345	253.6339
	Pore3	-0.2134	9.3206	0.0134	1.8372	230.4325
**L-MA**	Pore1	0.0840	1.5154	0.0887	2.9758	24.6745
	Pore2	0.0833	1.5906	0.1425	3.0196	23.5793
	Pore3	0.0836	1.5850	0.1062	3.1154	24.7442
**L-TA**	Pore1	0.1108	1.956	-0.3567	9.2292	67.7265
	Pore2	0.1146	1.7647	-0.3907	9.9803	86.5035
	Pore3	0.1106	2.0775	-0.2963	6.6427	82.8009
**CA**	Pore1	0.1652	6.7449	2.7832	18.3329	13372.9021
	Pore2	0.1629	6.8852	2.5484	17.1089	17059.2494
	Pore3	0.1639	6.8825	2.3242	14.7278	14775.2773
**ICIT**	Pore1	0.1435	8.8025	1.3938	4.3364	50.2974
	Pore2	0.1430	8.5306	1.4418	4.5831	50.6782
	Pore3	0.1442	8.6651	1.4261	4.6128	49.9132
**D-GLC**	Pore1	-0.1787	4.8944	0.3515	4.7794	297.0913
	Pore2	-0.1873	4.9145	0.1525	4.8329	337.2057
	Pore3	-0.1820	4.9386	0.2175	4.9051	201.2251
**D-FRU**	Pore1	-0.2032	6.7025	-0.0749	7.7636	1760.2389
	Pore2	-0.2024	6.6632	-0.0471	8.1890	1424.1835
	Pore3	-0.2029	6.6405	-0.0721	7.8646	1601.5371

Supplementary Table 3. Accuracies of different models. The training set consists of 5600 events (n = 1000 for D-FRU and D-GLC, n = 500 for CAT, 3-CQA, D-SOR, XYL, L-MA, L-TA, ICIT, and n = 100 for CA). For each analyte, the event number of the testing set was one-fifth of the training set. All models were trained and tested using the Classification Learner toolbox in MATLAB. The validation accuracies were calculated by 10-fold cross-validation of the training set, and the testing accuracies were calculated by the validation of the testing set. The Bagging Trees model exhibits the highest accuracy. It is therefore selected for further applications.

**Model**		**Validation Accuracy**	**Testing Accuracy**
**Decesion Tree**	Fine Tree	98.8%	98.8%
	Medium Tree	97.2%	97.6%
	Coarse Tree	53.6%	53.6%
**Discriminant Analysis**	Linear Discriminant	82.8%	84.1%
	Quadratic Discriminant	87.1%	87.5%
**Naïve Bayes**	Gaussian Naïve Bayes	85.8%	85.8%
	Kernel Naïve Bayes	95.2%	95.5%
**SVM**	Linear SVM	93.7%	94.1%
	Quadratic SVM	97.8%	97.8%
	Fine Gaussian SVM	96.9%	97.3%
**KNN**	Fine KNN	97.9%	97.8%
	Cubic KNN	96.6%	96.3%
	Weighted KNN	97.4%	97.4%
**Ensemble**	Boosted Trees	99.0%	98.8%
	Bagging Trees	99.3%	99.1%
	RUSBoost Trees	98.8%	98.4%
**Neural Network**	Narrow Neural Network	98.4%	98.2%
	Medium Neural Network	98.4%	97.9%
	Wide Neural Network	98.4%	98.4%

Supplementary Table 4. Calibration coefficients of ten target *cis*-diols. The calibration coefficients (k_i) is defined as the number of event occurrences for each *cis*-diol type per unit concentration (mM) per unit time (ms). The value was derived from results of linear fitting of the concentration dependence assay shown in **Supplementary Figs. 14-23**. All nanopore measurements were performed using MspA-PBA in a buffer of 1.5 M KCl, 100 mM MOPS, pH 7.0 and a continually applied bias of +160 mV.

**Analyte**	k **(mM^{-1}*ms^{-1})**
CAT	6.89E-3
3-CQA	1.39E-2
D-SOR	3.44E-4
XYL	4.85E-4
L-MA	1.30E-3
L-TA	6.28E-4
CA	8.48E-6
ICIT	2.12E-3
D-GLC	1.80E-5
D-FRU	1.99E-4

Supplementary Table 5. The concentrations (C_i) of *cis*-diols in each fruit juice. All measurements were performed as described in **Methods**. A +160 mV bias was continually applied during the measurements. To initiate the measurement, 5 μL prune juice, 5 μL grape juice and 15 μL lemon juice were respectively added to the *cis* and *trans* chambers to initiate the measurement (**Fig. 5**). The measured values were acquired as demonstrated in **Methods**. Three independent trails were performed for each condition.

Analyte	C_i in Prune/(mM)	C_i in Grape/(mM)	C_i in Lemon/(mM)
CAT	N.A.	N.A.	N.A.
3-CQA	0.14±0.01	N.A.	N.A.
D-SOR	53±4	--	N.A.
XYL	--	0.26±0.12	N.A.
L-MA	12.4±1.0	9±4	2.4±0.9
L-TA	N.A.	10±3	--
CA	N.A.	N.A.	60±15
ICIT	N.A.	N.A.	0.41±0.05
D-GLC	90±50	90±50	30±20
D-FRU	41±8	130±40	13±7

N.A. stands for that no events of this analyte were detected;

--, stands for that the corresponding event of the analyte was too low to for concentration calculation .

Supplementary Table 6. The pH value and ion strength of the electrical buffer after addition of fruit juice. All measurements were performed as described in **Methods**. 5 μL prune juice, 5 μL grape juice and 15 μL lemon juice were respectively added to both chambers for nanopore measurements (**Fig. 5**). The concentrations of juice were consistent with that in nanopore measurements. After addition of juice, the values of pH were consistent with the electrical buffer of 1.5 M KCl, 100 mM MOPS, pH 7.0 used in nanopore measurements. Besides that, the open current values almost not changed before and after juice addition, indicating that juice addition doesn't change ion strength in measurement.

Analytes / samples	concentration	pH	current before addition (pA)	current after addition (pA)
Prune juice	0.99% (v:v)	6.95	377	380
Grape juice	0.99% (v:v)	6.96	372	370
Lemon juice	2.91% (v:v)	6.80	379	373.1

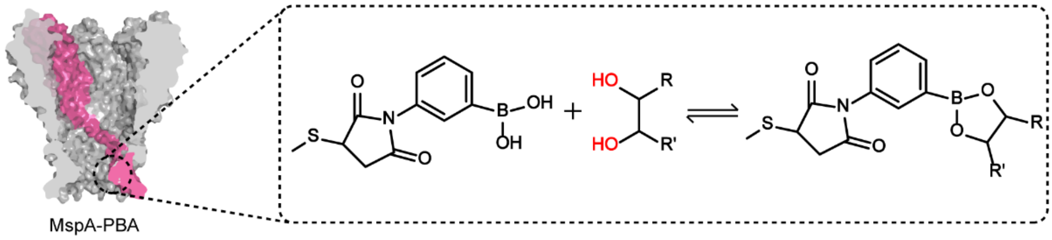

Supplementary Figure 1. The sensing mechanism. An MspA-PBA is consisted of one unit of N90C MspA-H6 (pink) modified with a 3-(maleimide) phenylboronic acid and seven units of M2 MspA-D16H6 (grey) without any modification. The phenylboronic acid in MspA-PBA can reversibly react with *cis*-diols, forming the general mechanism of sensing.

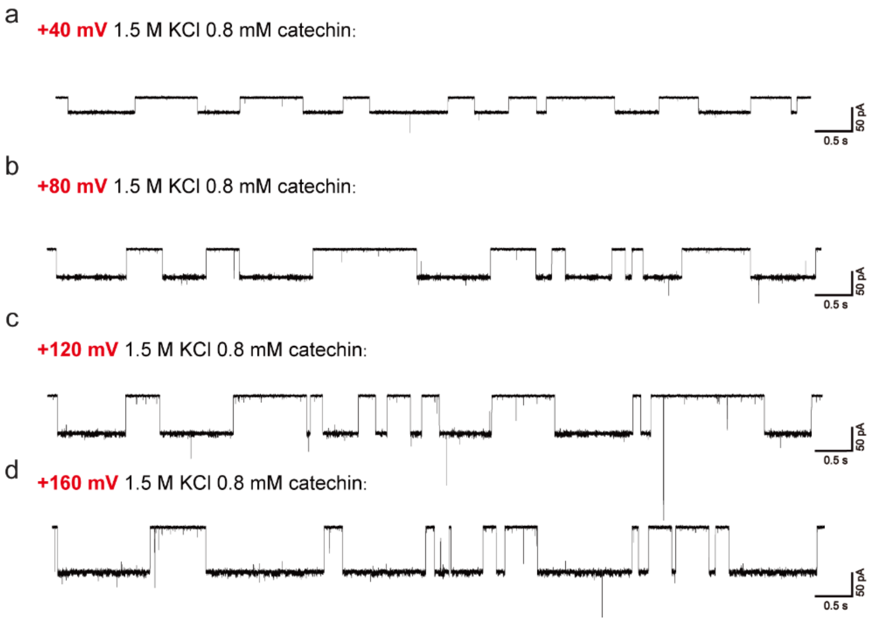

Supplementary Figure 2. The voltage dependence of nanopore sensing. (a-d) Representative traces acquired with catechin however measured with a voltage between +40 mV to +160 mV. Here, catechin was applied as model analyte to probe the voltage dependence of nanopore events. Clearly, at a higher applied voltage, the nanopore event amplitude is generally increased. Catechin was added to both of measurement clambers with a final concentration of 0.8 mM. All measurements were carried out using MspA-PBA in a buffer of 1.5 M KCl, 100 mM MOPS, pH 7.0.

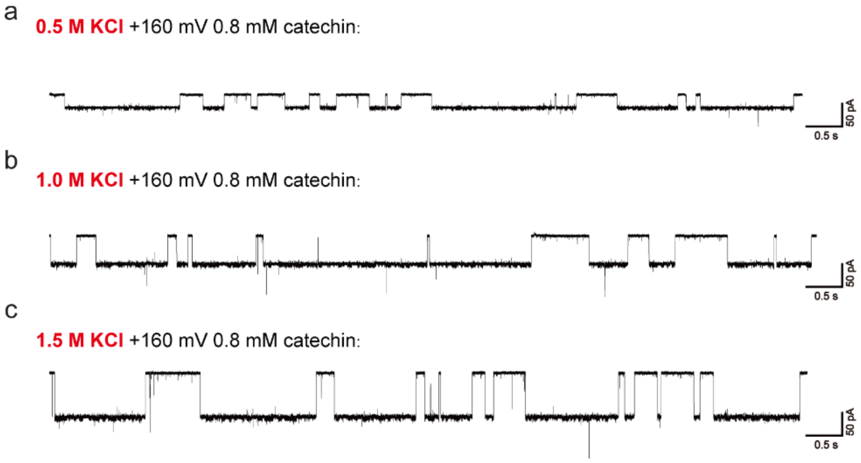

Supplementary Figure 3. The salt concentration dependence of nanopore measurement. (a-c) Representative traces respectively acquired with catechin however with buffers of a 0.5 M, 1 M and 1.5 M KCl. Here, catechin was applied as a model analyte to probe the salt concentration of nanopore events. Clearly, with a higher salt concentration, the nanopore event amplitude is higher, reporting a more superior discrimination resolution. Catechin was added to both of measurement clambers with a final concentration of 0.8 mM. All measurements were carried out using MspA-PBA with a continually applied bias of +160 mV.

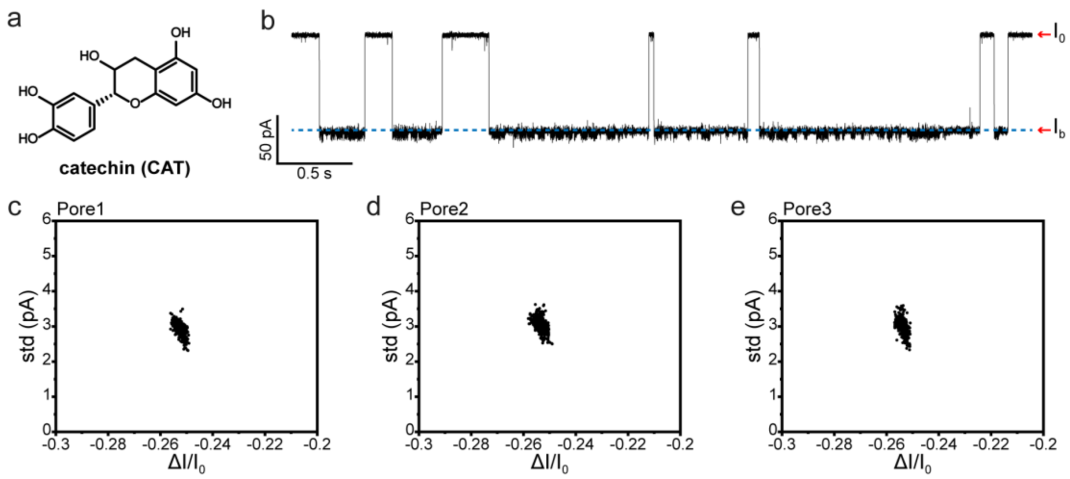

Supplementary Figure 4. Events of CAT. (a) The chemical structure of CAT. **(b)** A representative trace acquired with CAT as the analyte. **(c-e)** Scatter plots of $\Delta I/I_0$ versus std for CAT events. Results in different scatter plots were respectively acquired from three independent measurements. Each scatter plot contains 500 events. The nanopore measurements were performed using MspA-PBA in a 1.5 M KCl buffer. CAT was added to *cis* and *trans* with a final concentration of 0.8 mM. A +160 mV bias was continually applied.

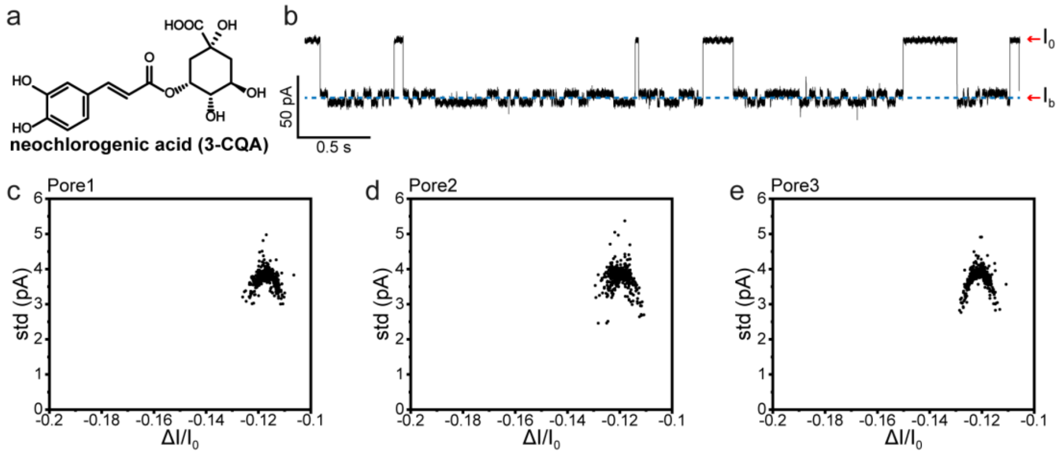

Supplementary Figure 5. Events of 3-CQA. (a) The chemical structure of 3-CQA. **(b)** A representative trace acquired with 3-CQA as the analyte. **(c-e)** Scatter plots of $\Delta I/I_0$ versus std for 3-CQA events. Results in different scatter plots were respectively acquired from three independent measurements. Each scatter plot contains 500 events. The nanopore measurements were performed using MspA-PBA in a 1.5 M KCl buffer. 3-CQA was added to *cis* and *trans* with a final concentration of 0.5 mM. A +160 mV bias was continually applied.

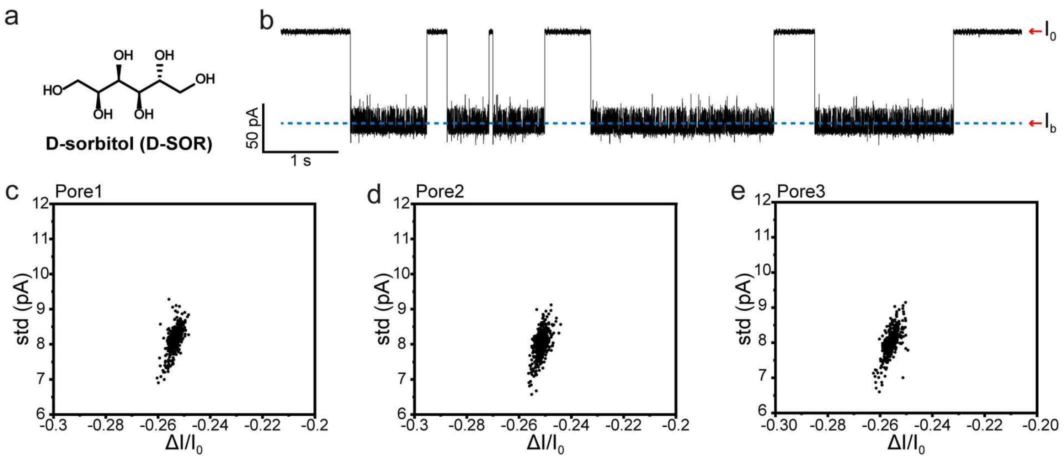

Supplementary Figure 6. Events of D-SOR. (a) The chemical structure of D-SOR. **(b)** A representative trace acquired with D-SOR as the analyte. **(c-e)** Scatter plots of *ΔI/I₀* versus std for D-SOR events. Results in different scatter plots were respectively acquired from three independent measurements. Each scatter plot contains 500 events. The nanopore measurements were performed using MspA-PBA in a 1.5 M KCl buffer. D-SOR was added to *cis* and *trans* with a final concentration of 2 mM. A +160 mV bias was continually applied.

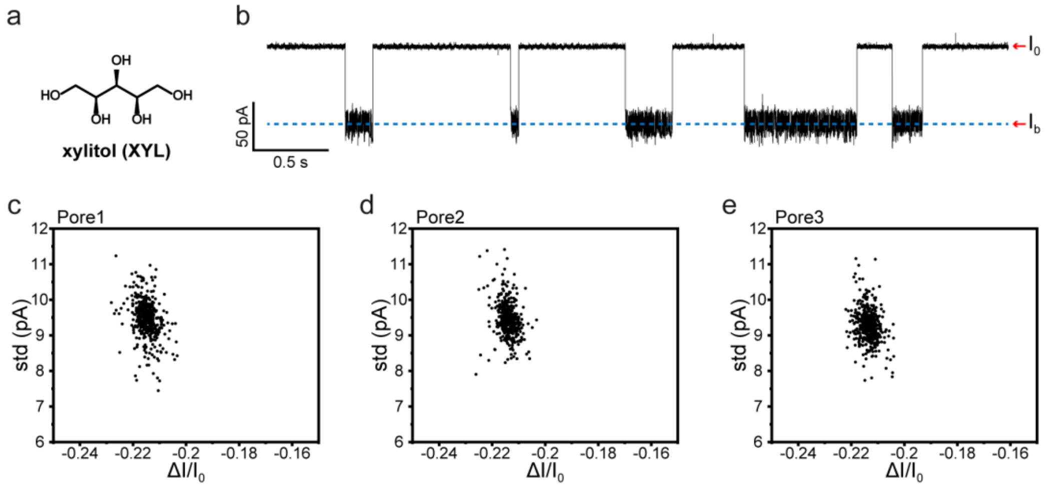

Supplementary Figure 7. Events of XYL. (a) The chemical structure of XYL. **(b)** A representative trace acquired with XYL as the analyte. **(c-e)** Scatter plots of $\Delta I/I_0$ versus std for XYL events. Results in different scatter plots were respectively acquired from three independent measurements. Each scatter plot contains 500 events. The nanopore measurements were performed using MspA-PBA in a 1.5 M KCl buffer. XYL was added to *cis* and *trans* with a final concentration of 2 mM. A +160 mV bias was continually applied.

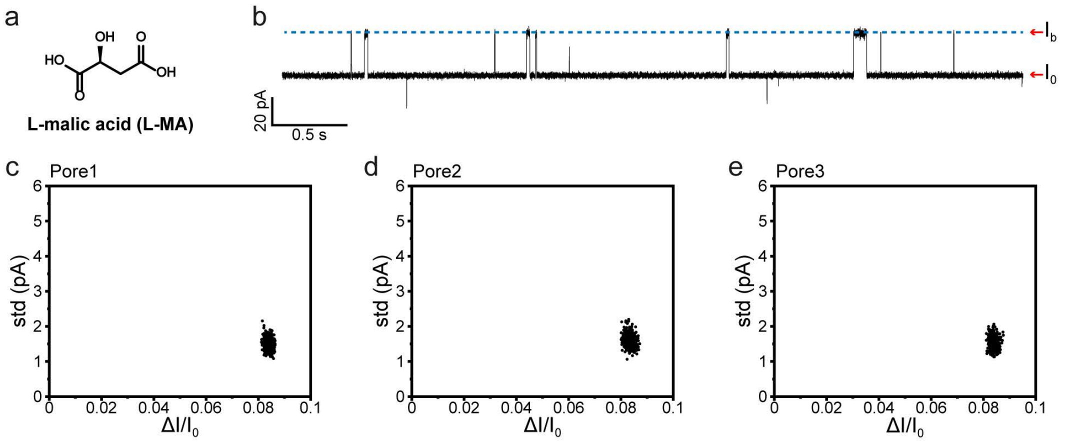

Supplementary Figure 8. Events of L-MA. (a) The chemical structure of L-MA. **(b)** A representative trace acquired with L-MA as the analyte. **(c-e)** Scatter plots of $\Delta I/I_0$ versus std for L-MA events. Results in different scatter plots were respectively acquired from three independent measurements. Each scatter plot contains 500 events. The nanopore measurements were performed using MspA-PBA in a 1.5 M KCl buffer. L-MA was added to *cis* and *trans* with a final concentration of 0.6 mM. A +160 mV bias was continually applied.

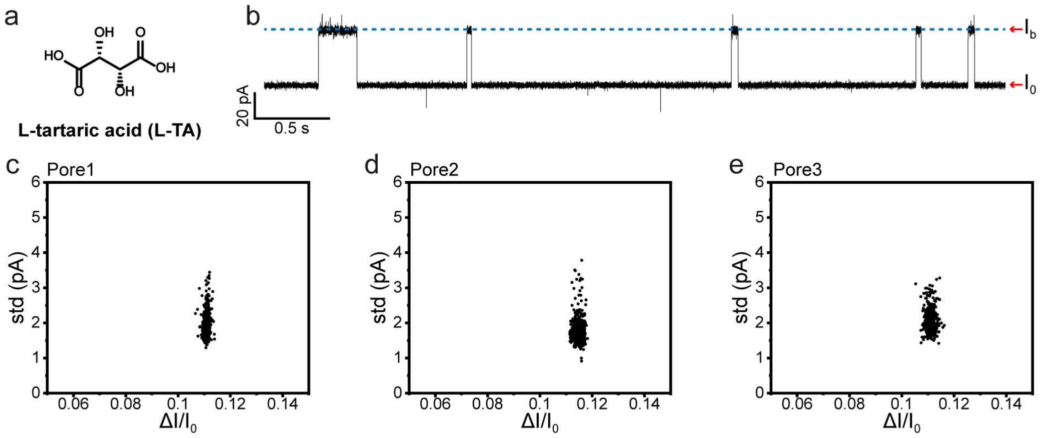

Supplementary Figure 9. Events of L-TA. (a) The chemical structure of L-TA. **(b)** A representative trace acquired with L-TA as the analyte. **(c-e)** Scatter plots of $\Delta I/I_0$ versus std for L-TA events. Results in different scatter plots were respectively acquired from three independent measurements. Each scatter plot contains 500 events. The nanopore measurements were performed using MspA-PBA in a 1.5 M KCl buffer. L-TA was added to *cis* and *trans* with a final concentration of 0.6 mM. A +160 mV bias was continually applied.

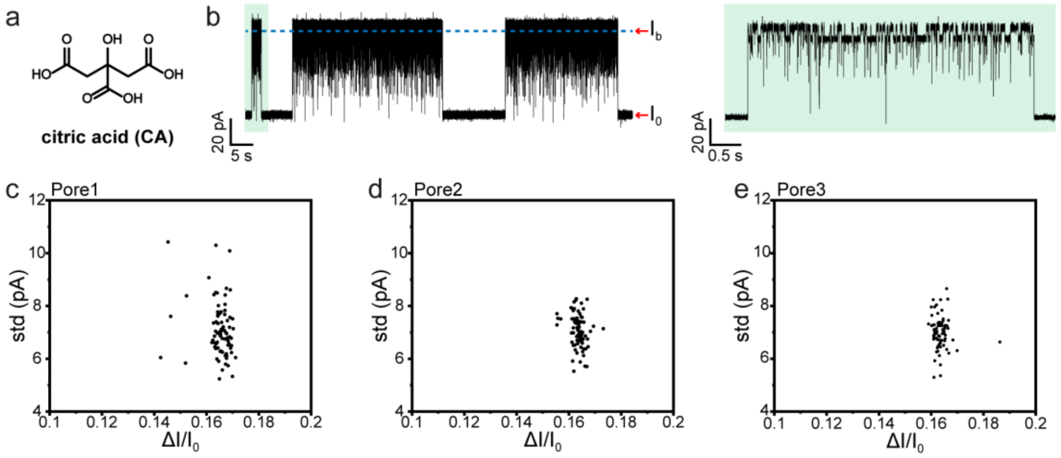

Supplementary Figure 10. Events of CA. (a) The chemical structure of CA. **(b)** A representative trace acquired with CA as the analyte. A zoomed-in view of the trace segment marked with a green rectangle is also presented to the right. **(c-e)** Scatter plots of $\Delta I/I_0$ versus std for CA events. Results in different scatter plots were respectively acquired from three independent measurements. Each scatter plot contains 100 events. The nanopore measurements were performed using MspA-PBA in a 1.5 M KCl buffer. CA was added to *cis* and *trans* with a final concentration of 6 mM. A +160 mV bias was continually applied.

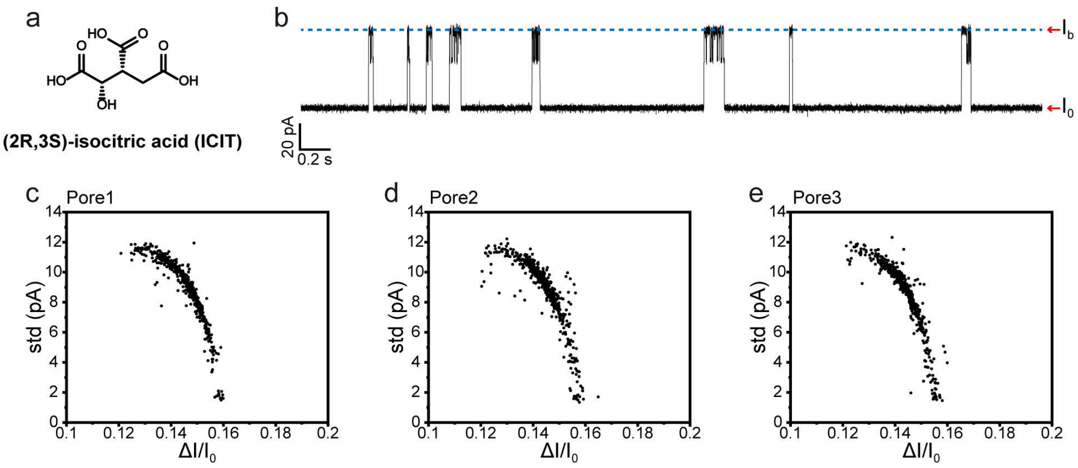

Supplementary Figure 11. Events of ICIT. (a) The chemical structure of ICIT. **(b)** A representative trace acquired with ICIT as the analyte. **(c-e)** Scatter plots of $\Delta I/I_0$ versus std for ICIT events. Results in different scatter plots were respectively acquired from three independent measurements. Each scatter plot contains 500 events. The nanopore measurements were performed using MspA-PBA in a 1.5 M KCl buffer. ICIT was added to *cis* and *trans* with a final concentration of 1 mM. A +160 mV bias was continually applied.

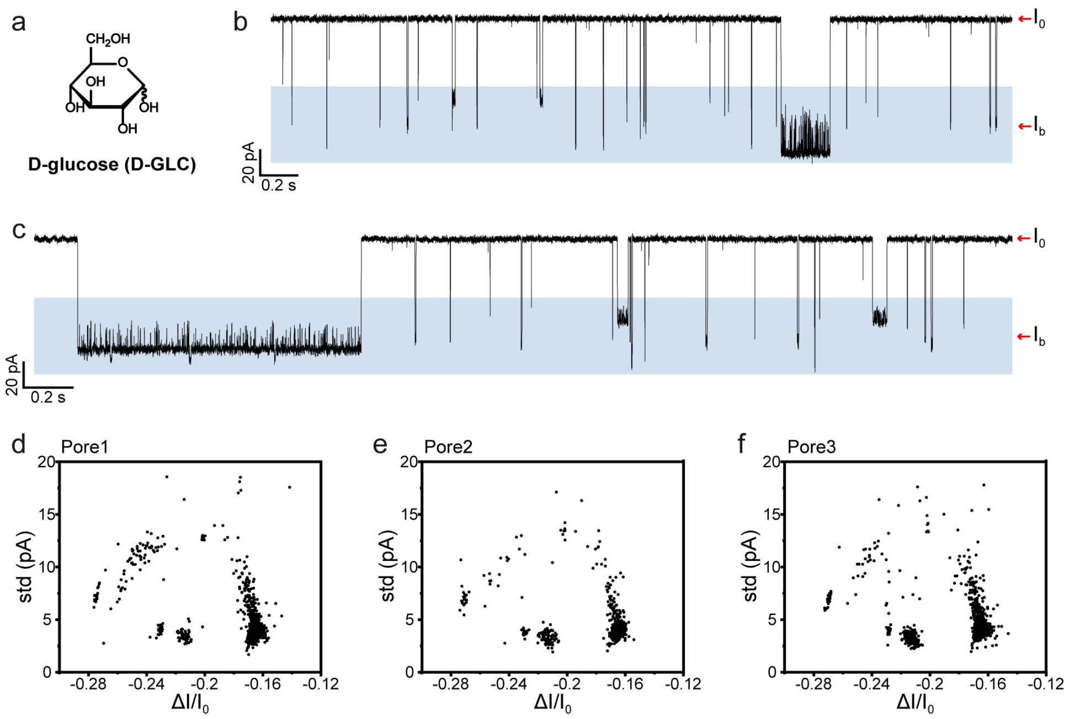

Supplementary Figure 12. Events of D-GLC. (a) The chemical structure of D-GLC. **(b-c)** Representative traces acquired with D-GLC as the ananlyte. **(d-f)** Scatter plots of $\Delta I/I_0$ versus std for D-GLC events. Results in different scatter plots were respectively acquired from three independent measurements. Each scatter plot contains 1000 events. The nanopore measurements were performed using MspA-PBA in a 1.5 M KCl buffer. D-GLC was added to *cis* and *trans* with a final concentration of 30 mM. A +160 mV bias was continually applied.

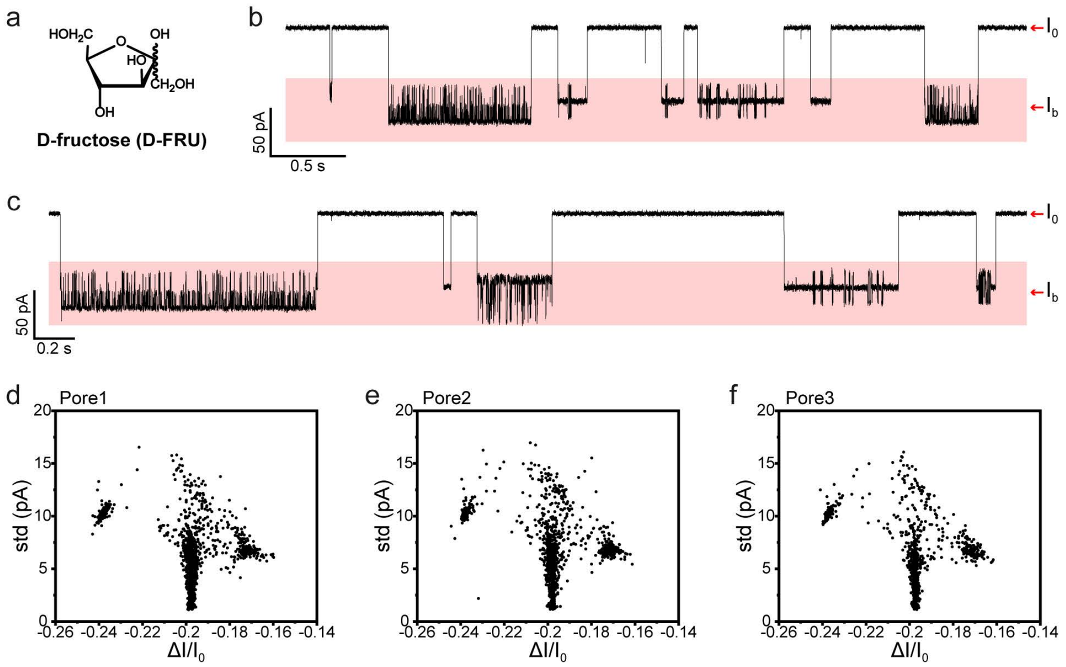

Supplementary Figure 13. Events of D-FRU. (a) The chemical structure of D-FRU. **(b-c)** Representative traces acquired with D-FRU as the analyte. **(d-f)** Scatter plots of *ΔI/I₀* versus std for D-FRU events. Results in different scatter plots were respectively acquired from three independent measurements. Each scatter plot contains 1000 events. The nanopore measurements were performed using MspA-PBA in a 1.5 M KCl buffer. D-FRU was added to *cis* and *trans* with a final concentration of 10 mM. A +160 mV bias was continually applied.

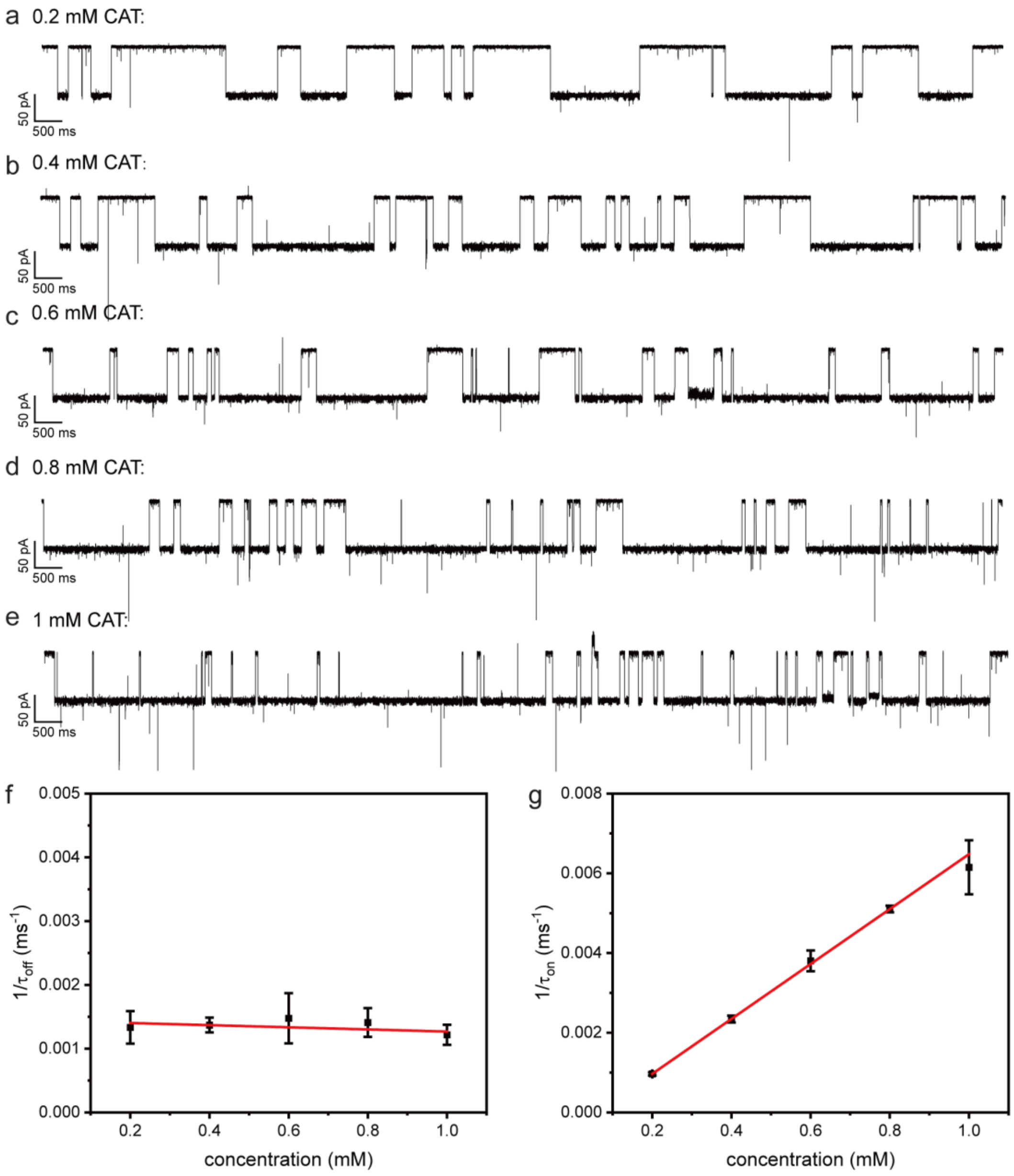

Supplementary Figure 14. The concentration dependence of CAT sensing. (a-e) Representative traces acquired with varying CAT concentrations. All nanopore measurements were performed using MspA-PBA in a buffer of 1.5 M KCl, 100 mM MOPS, pH 7.0 and a continually applied bias of +160 mV **(Methods)**. CAT was added to both of measurement chambers with a final concentration of 0.2-1 mM. **(f)** The plot of $1/\tau_{off}$ versus the CAT concentration. The $1/\tau_{off}$ remains constant as CAT concentration varies. **(g)** The plot of $1/\tau_{on}$ versus the CAT concentration. $1/\tau_{on}$ is linearly correlated with the CAT concentration. Three independent measurements were performed to acquire the statistic results (N=3). All statistics are also described in **Supplementary Table 1.** The data in **(f-g)** show mean ± standard deviations derived from results of three independent measurements (N=3). The error bars represent standard deviation values.

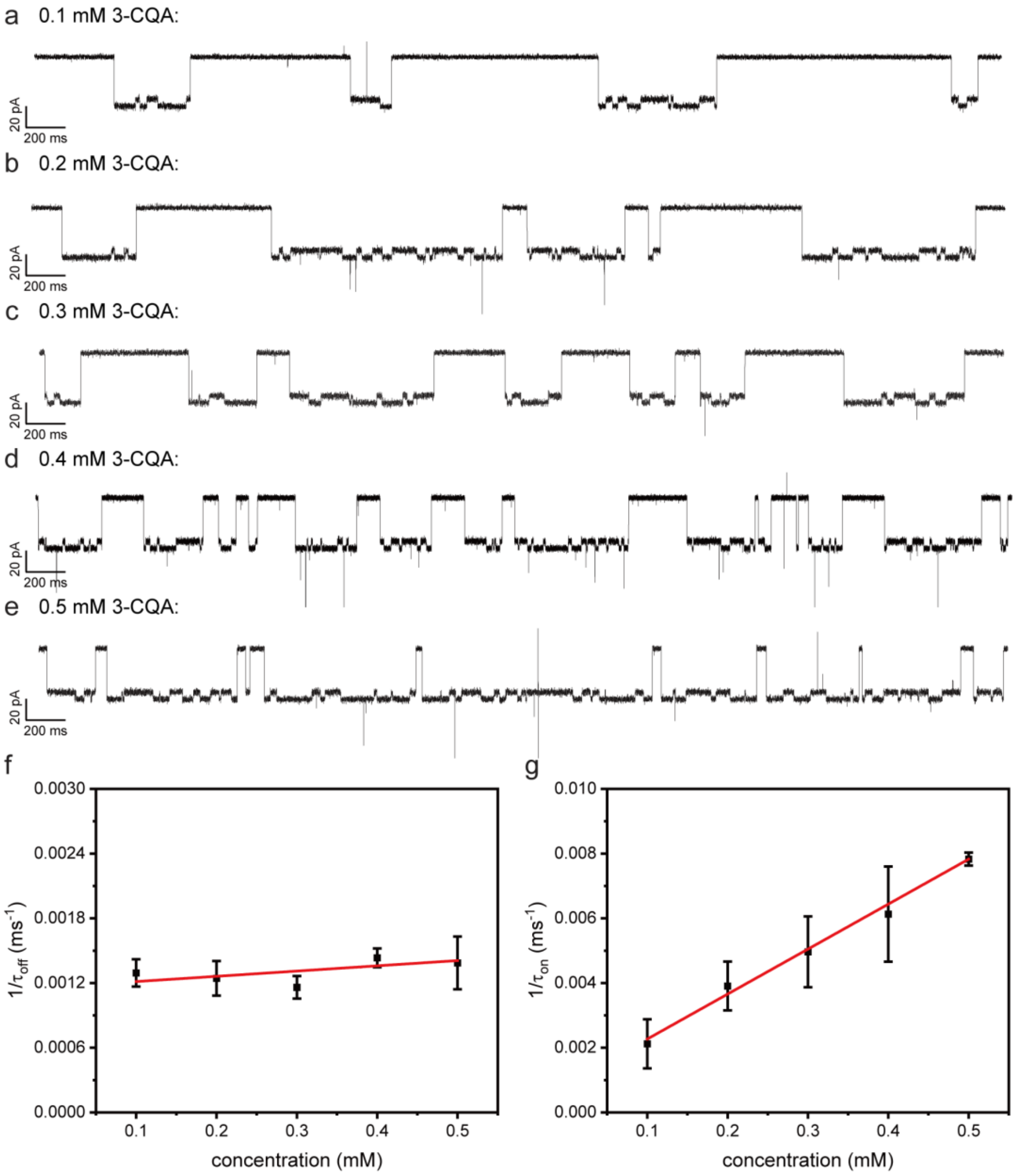

Supplementary Figure 15. The concentration dependence of 3-CQA sensing. (a-e) Representative traces acquired with varying 3-CQA concentrations. All nanopore measurements were performed using MspA-PBA in a buffer of 1.5 M KCl, 100 mM MOPS, pH 7.0 and a continually applied bias of +160 mV **(Methods)**. 3-CQA was added to both of measurement chambers with a final concentration of 0.1-0.5 mM. **(f)** The plot of $1/\tau_{off}$ versus the 3-CQA concentration. The $1/\tau_{off}$ remains constant as 3-CQA concentration varies. **(g)** The plot of $1/\tau_{on}$ versus the 3-CQA concentration. $1/\tau_{on}$ is linearly correlated with the 3-CQA concentration. Three independent measurements were performed to acquire the statistic results (N=3). All statistics are also described in **Supplementary Table 1.** The data in **(f-g)** show mean ± standard deviations derived from results of three independent measurements (N=3). The error bars represent standard deviation values.

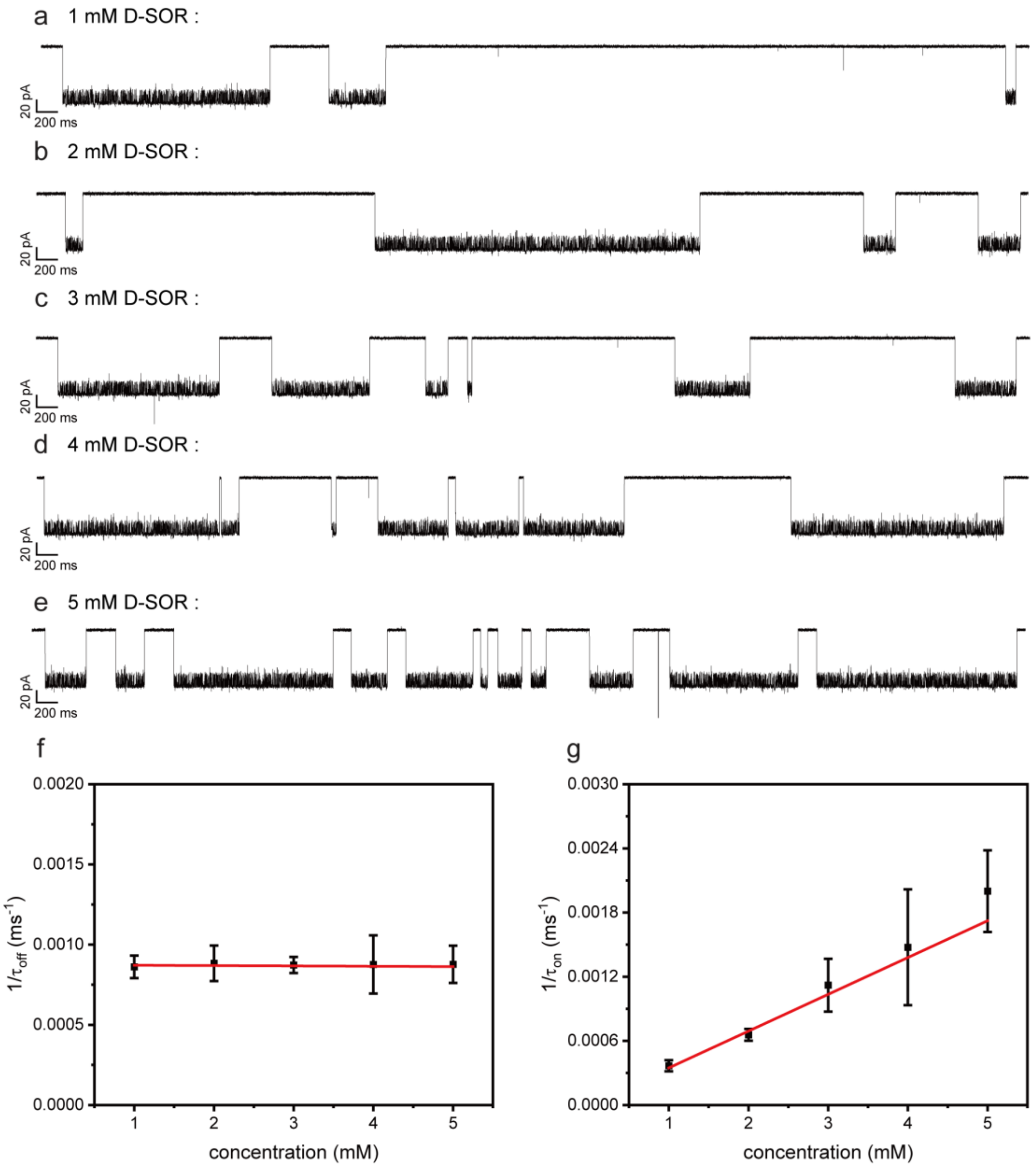

Supplementary Figure 16. The concentration dependence of D-SOR sensing. (a-e) Representative traces acquired with varying D-SOR concentrations. All nanopore measurements were performed using MspA-PBA in a buffer of 1.5 M KCl, 100 mM MOPS, pH 7.0 and a continually applied bias of +160 mV **(Methods)**. D-SOR was added to both of measurement chambers with a final concentration of 1-5 mM. **(f)** The plot of $1/\tau_{off}$ versus the D-SOR concentration. The $1/\tau_{off}$ remains constant as D-SOR concentration varies. **(g)** The plot of $1/\tau_{on}$ versus the D-SOR concentration. $1/\tau_{on}$ is linearly correlated with the D-SOR concentration. Three independent measurements were performed to acquire the statistic results (N=3). All statistics are also described in **Supplementary Table 1.** The data in **(f-g)** show mean ± standard deviations derived from results of three independent measurements (N=3). The error bars represent standard deviation values.

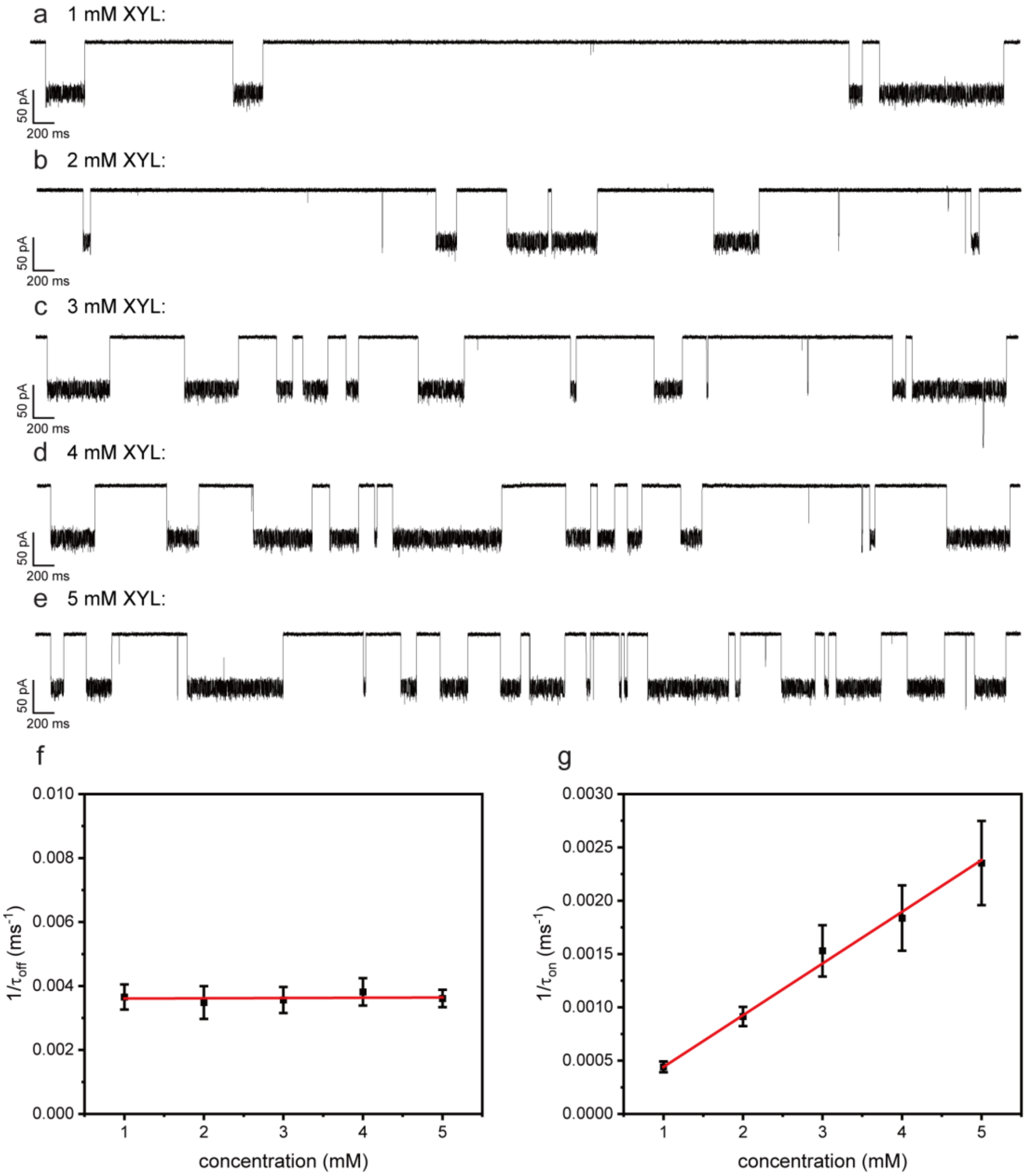

Supplementary Figure 17. The concentration dependence of XYL sensing. (a-e) Representative traces acquired with varying XYL concentrations. All nanopore measurements were performed using MspA-PBA in a buffer of 1.5 M KCl, 100 mM MOPS, pH 7.0 and a continually applied bias of +160 mV **(Methods)**. XYL was added to both of measurement chambers with a final concentration of 1-5 mM. **(f)** The plot of $1/\tau_{off}$ versus the XYL concentration. The $1/\tau_{off}$ remains constant as XYL concentration varies. **(g)** The plot of $1/\tau_{on}$ versus the XYL concentration. $1/\tau_{on}$ is linearly correlated with the XYL concentration. Three independent measurements were performed to acquire the statistic results (N=3). All statistics are also described in **Supplementary Table 1.** The data in **(f-g)** show mean ± standard deviations derived from results of three independent measurements (N=3). The error bars represent standard deviation values.

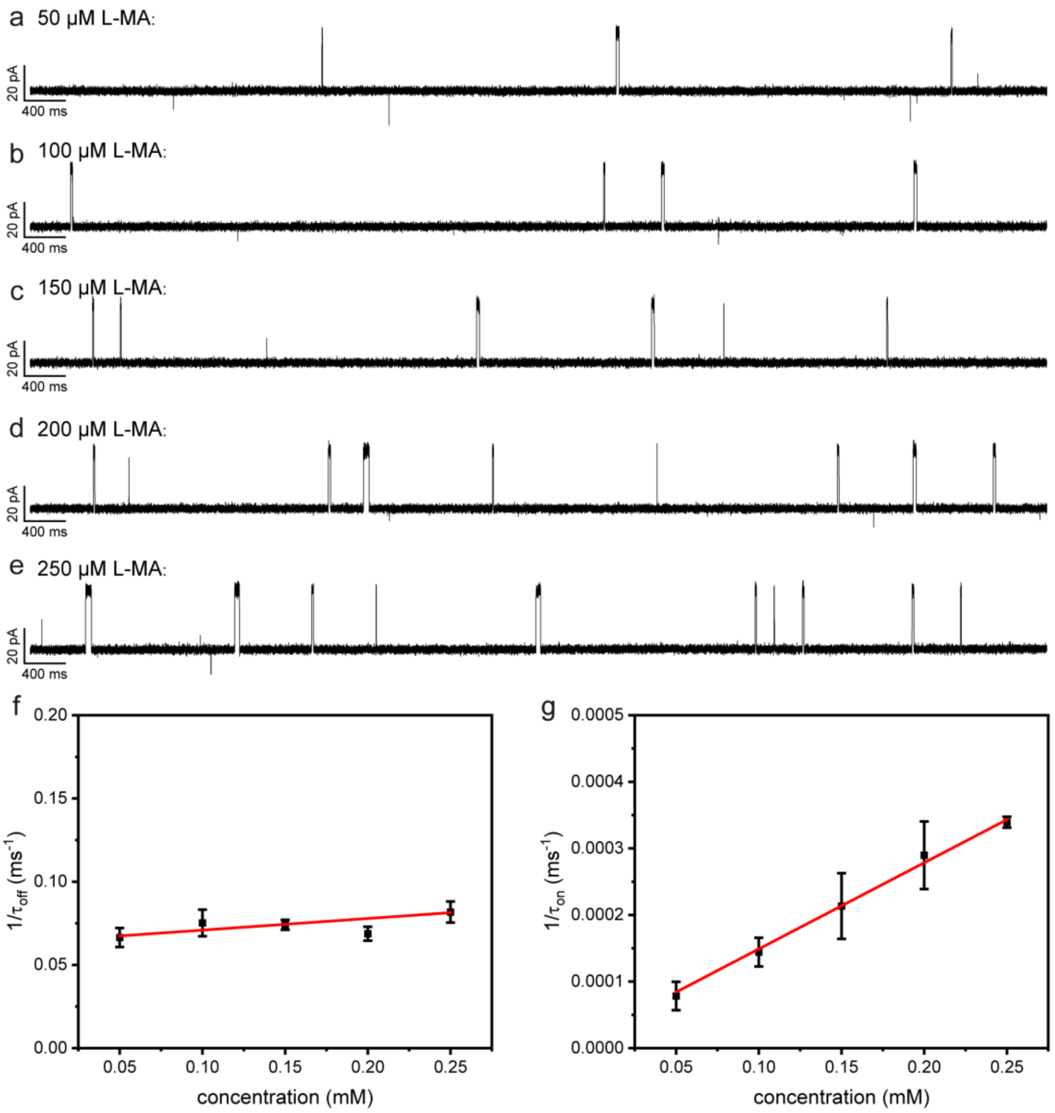

Supplementary Figure 18. The concentration dependence of L-MA sensing. (a-e) Representative traces acquired with varying L-MA concentrations. All nanopore measurements were performed using MspA-PBA in a buffer of 1.5 M KCl, 100 mM MOPS, pH 7.0 and a continually applied bias of +160 mV **(Methods)**. L-MA was added to both of measurement chambers with a final concentration of 0.05-0.25 mM. **(f)** The plot of $1/\tau_{off}$ versus the L-MA concentration. The $1/\tau_{off}$ remains constant as L-MA concentration varies. **(g)** The plot of $1/\tau_{on}$ versus the L-MA concentration. $1/\tau_{on}$ is linearly correlated with the L-MA concentration. Three independent measurements were performed to acquire the statistic results (N=3). All statistics are also described in **Supplementary Table 1.** The data in **(f-g)** show mean ± standard deviations derived from results of three independent measurements (N=3). The error bars represent standard deviation values.

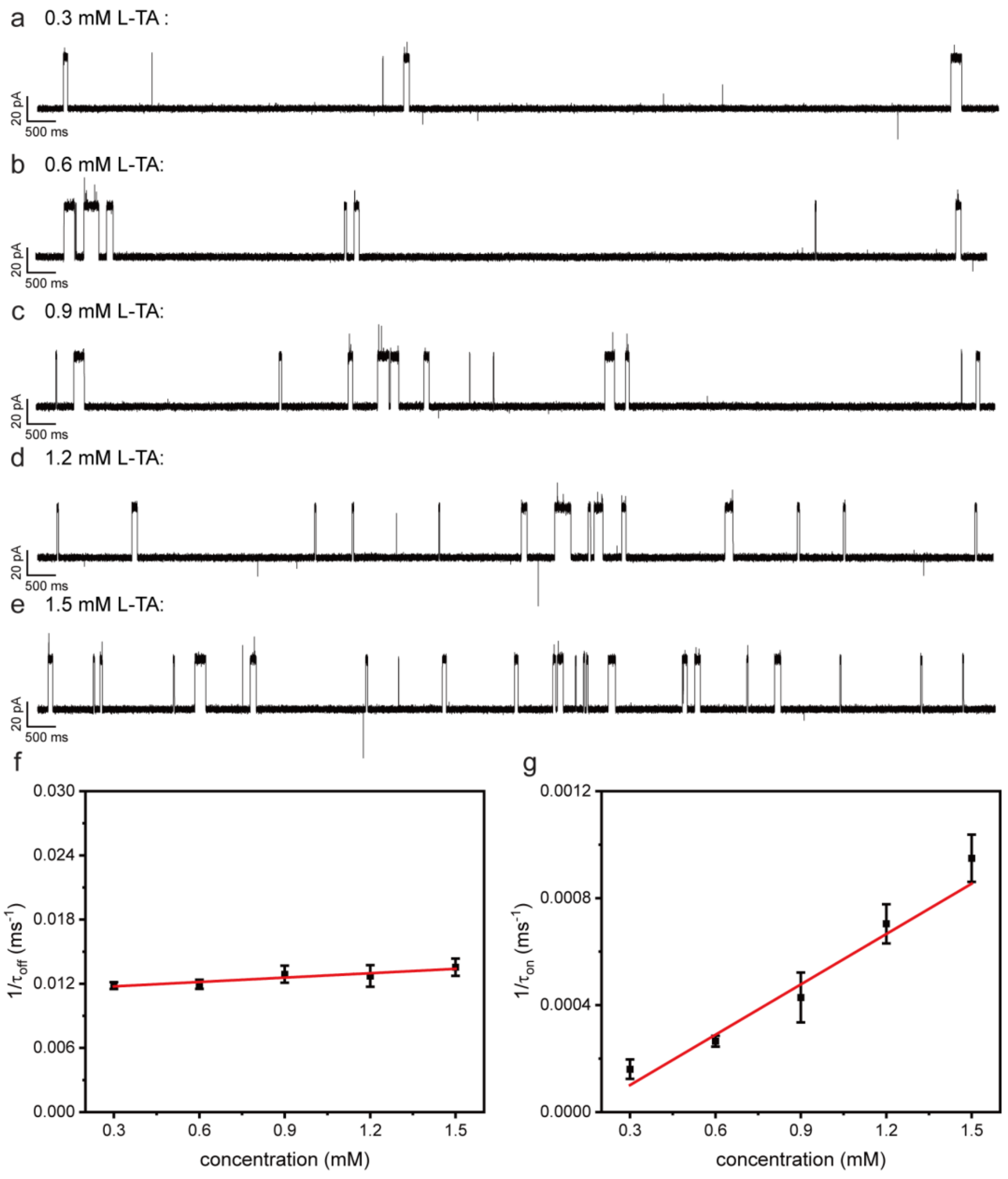

Supplementary Figure 19. The concentration dependence of L-TA sensing. (a-e) Representative traces acquired with varying L-TA concentrations. All nanopore measurements were performed using MspA-PBA in a buffer of 1.5 M KCl, 100 mM MOPS, pH 7.0 and a continually applied bias of +160 mV **(Methods)**. L-TA was added to both of measurement chambers with a final concentration of 0.3-1.5 mM. **(f)** The plot of $1/\tau_{off}$ versus the L-TA concentration. The $1/\tau_{off}$ remains constant as L-TA concentration varies. **(g)** The plot of $1/\tau_{on}$ versus the L-TA concentration. $1/\tau_{on}$ is linearly correlated with the L-TA concentration. Three independent measurements were performed to acquire the statistic results (N=3). All statistics are also described in **Supplementary Table 1.** The data in **(f-g)** show mean ± standard deviations derived from results of three independent measurements (N=3). The error bars represent standard deviation values.

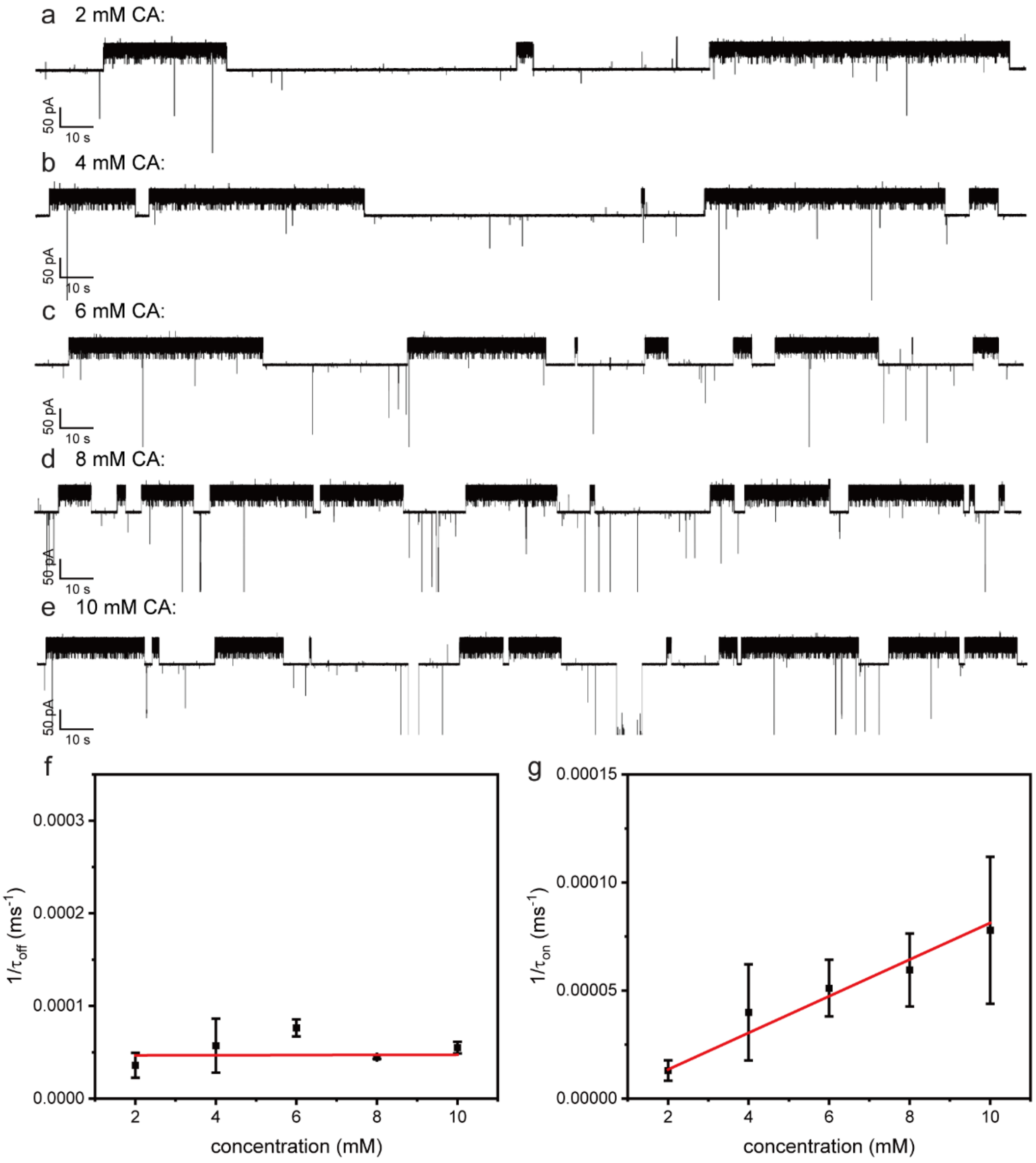

Supplementary Figure 20. The concentration dependence of CA sensing. (a-e) Representative traces acquired with varying CA concentrations. All nanopore measurements were performed using MspA-PBA in a buffer of 1.5 M KCl, 100 mM MOPS, pH 7.0 and a continually applied bias of +160 mV **(Methods)**. CA was added to both of measurement chambers with a final concentration of 2-10 mM. **(f)** The plot of $1/\tau_{off}$ versus the CA concentration. The $1/\tau_{off}$ remains constant as CA concentration varies. **(g)** The plot of $1/\tau_{on}$ versus the CA concentration. $1/\tau_{on}$ is linearly correlated with the CA concentration. Three independent measurements were performed to acquire the statistic results (N=3). All statistics are also described in **Supplementary Table 1.** The data in **(f-g)** show mean ± standard deviations derived from results of three independent measurements (N=3). The error bars represent standard deviation values.

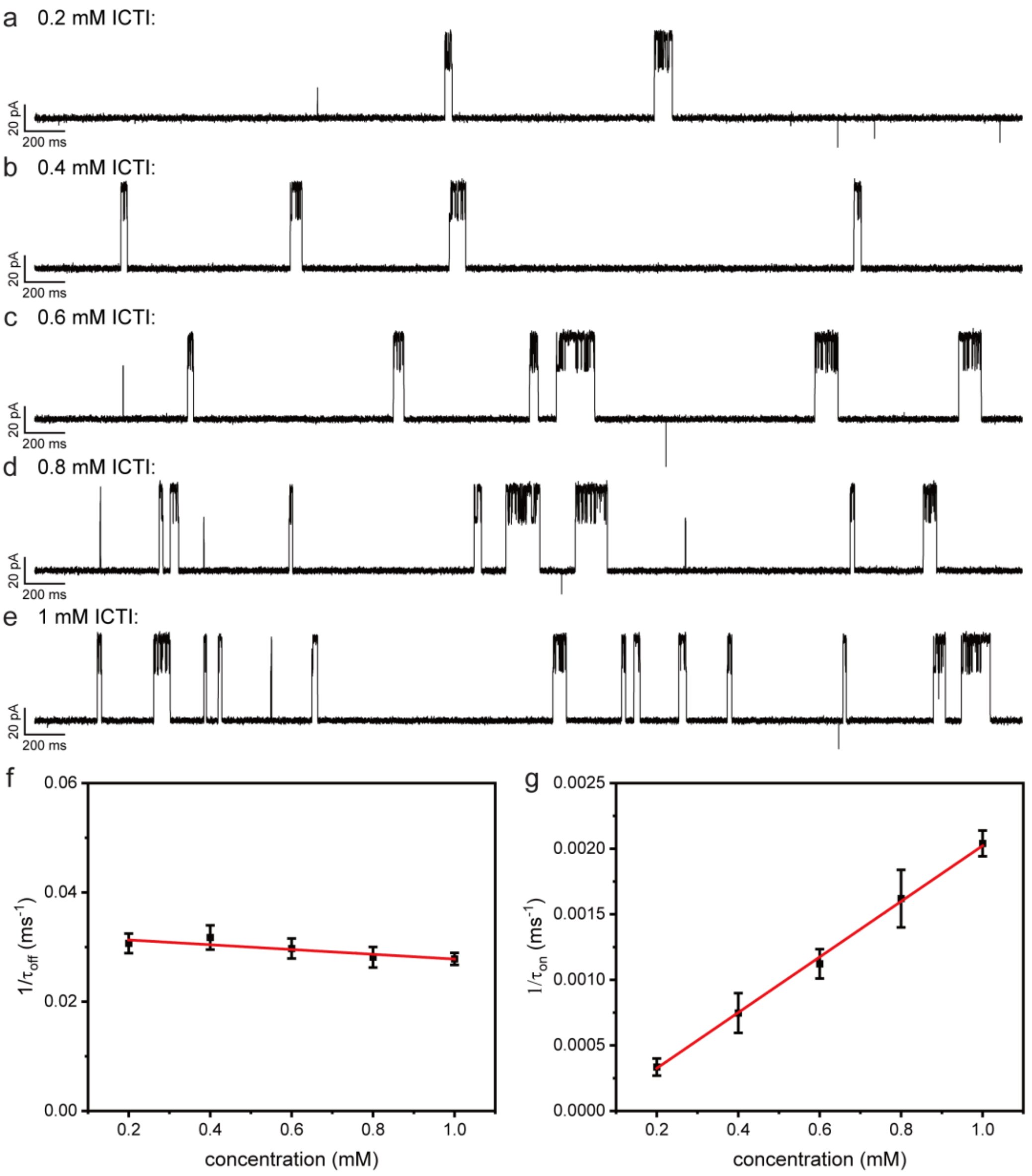

Supplementary Figure 21. The concentration dependence of ICIT sensing. (a-e) Representative traces acquired with varying ICIT concentrations. All nanopore measurements were performed using MspA-PBA in a buffer of 1.5 M KCl, 100 mM MOPS, pH 7.0 and a continually applied bias of +160 mV **(Methods)**. ICIT was added to both of measurement chambers with a final concentration of 0.2-1.0 mM. **(f)** The plot of $1/\tau_{off}$ versus the ICIT concentration. The $1/\tau_{off}$ remains constant as ICIT concentration varies. **(g)** The plot of $1/\tau_{on}$ versus the ICIT concentration. $1/\tau_{on}$ is linearly correlated with the ICIT concentration. Three independent measurements were performed to acquire the statistic results (N=3). All statistics are also described in **Supplementary Table 1.** The data in **(f-g)** show mean ± standard deviations derived from results of three independent measurements (N=3). The error bars represent standard deviation values.

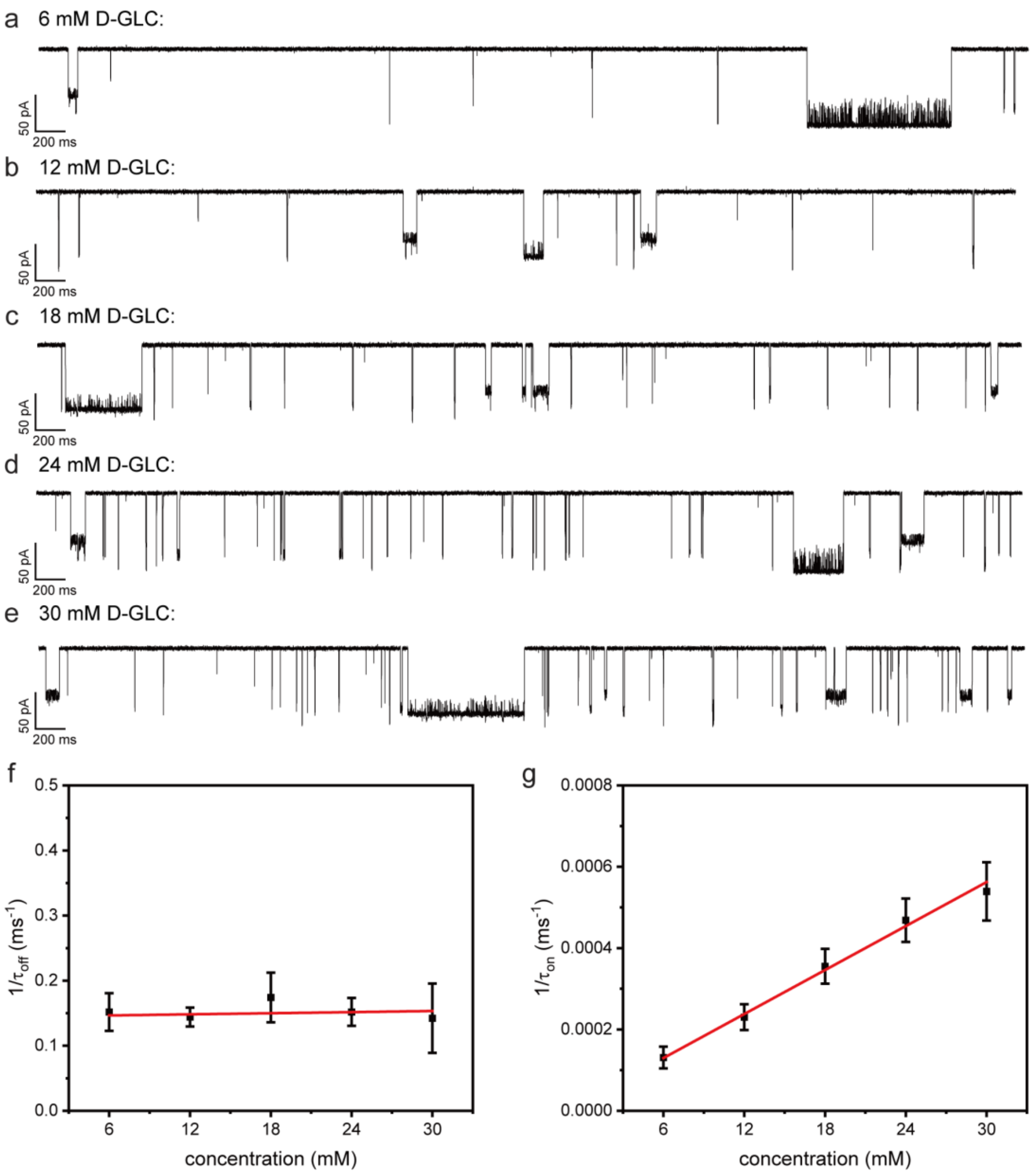

Supplementary Figure 22. The concentration dependence of D-GLC sensing. (a-e) Representative traces acquired with varying D-GLC concentrations. All nanopore measurements were performed using MspA-PBA in a buffer of 1.5 M KCl, 100 mM MOPS, pH 7.0 and a continually applied bias of +160 mV **(Methods)**. D-GLC was added to both of measurement chambers with a final concentration of 6-30 mM. **(f)** The plot of $1/\tau_{off}$ versus the D-GLC concentration. The $1/\tau_{off}$ remains constant as D-GLC concentration varies. **(g)** The plot of $1/\tau_{on}$ versus the D-GLC concentration. $1/\tau_{on}$ is linearly correlated with the D-GLC concentration. Three independent measurements were performed to acquire the statistic results (N=3). All statistics are also described in **Supplementary Table 1.** The data in **(f-g)** show mean ± standard deviations derived from results of three independent measurements (N=3). The error bars represent standard deviation values.

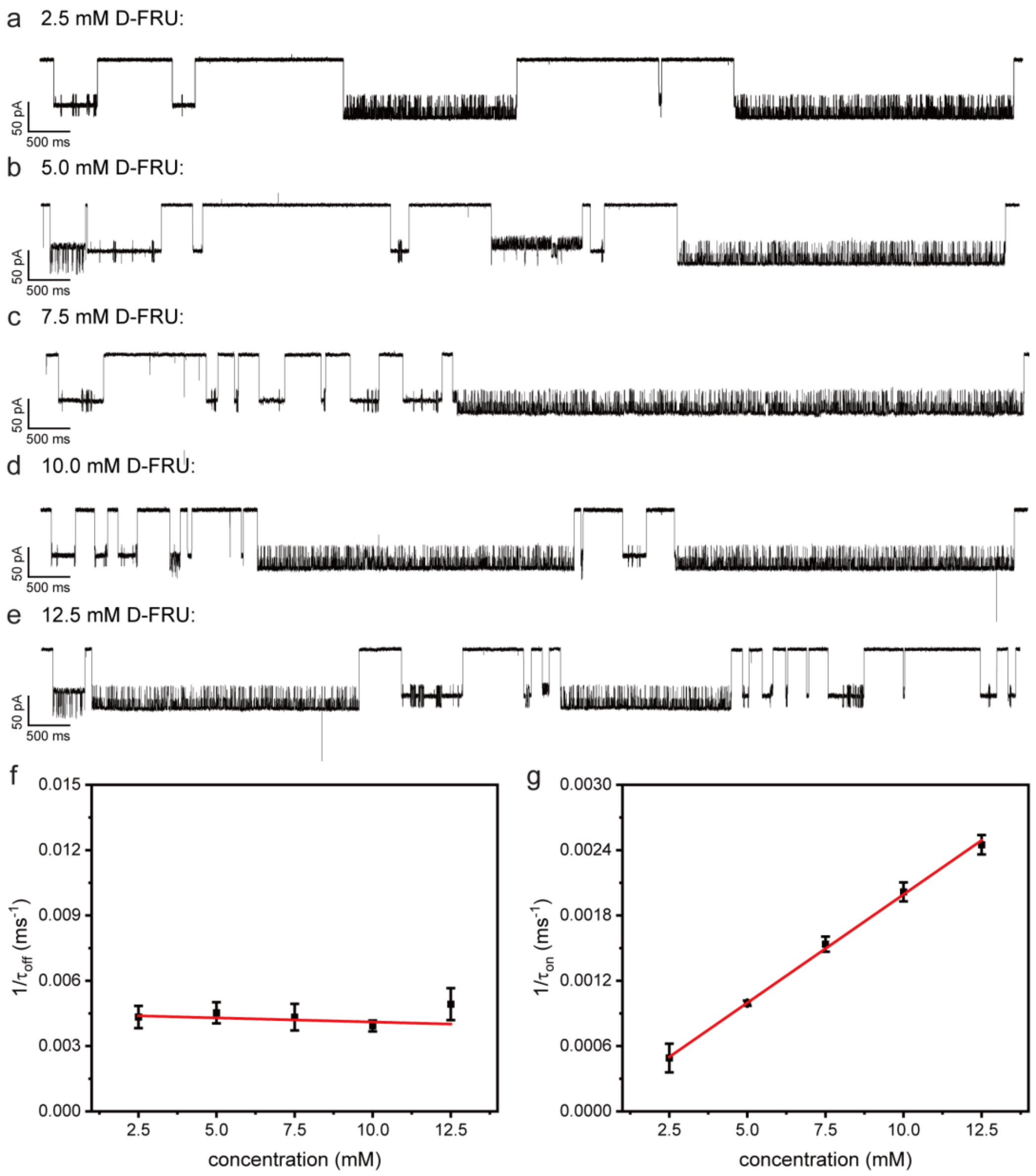

Supplementary Figure 23. The concentration dependence of D-FRU sensing. (a-e) Representative traces acquired with varying D-FRU concentrations. All nanopore measurements were performed using MspA-PBA in a buffer of 1.5 M KCl, 100 mM MOPS, pH 7.0 and a continually applied bias of +160 mV **(Methods)**. D-FRU was added to both of measurement chambers with a final concentration of 2.5-12.5 mM. **(f)** The plot of $1/\tau_{off}$ versus the D-FRU concentration. The $1/\tau_{off}$ remains constant as D-FRU concentration varies. **(g)** The plot of $1/\tau_{on}$ versus the D-FRU concentration. $1/\tau_{on}$ is linearly correlated with the D-FRU concentration. Three independent measurements were performed to acquire the statistic results (N=3). All statistics are also described in **Supplementary Table 1.** The data in **(f-g)** show mean ± standard deviations derived from results of three independent measurements (N=3). The error bars represent standard deviation values.

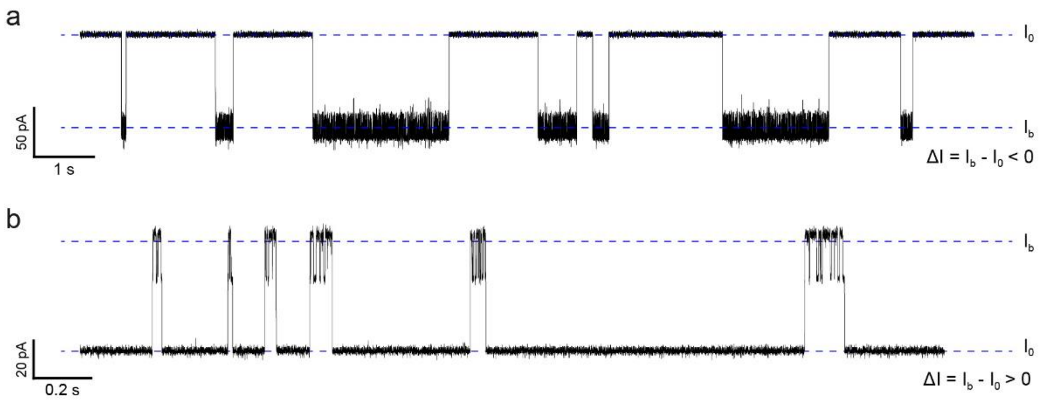

Supplementary Figure 24. The demonstration of positive and negative going events. (a, b) Representative traces respectively acquired with D-SOR **(a)** or ICIT **(b)** as the analytes. The open pore current (I_0) and the residual current (I_b) were marked on the trace. The blockage amplitude (ΔI) was defined as $I_b - I_0$. Events of D-SOR are negative going events ($\Delta I < 0$). Whereas, events of ICIT are positive going events ($\Delta I > 0$).

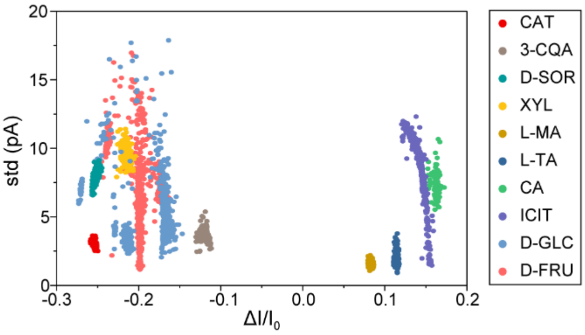

Supplementary Figure 25. The scatter plot of *$\Delta I/I_0$* versus std for ten target *cis*-diols. Events of ten types of *cis*-diols were respectively acquired from ten independent measurements (n = 1000 for D-FRU and D-GLC, n = 500 for CAT, 3-CQA, D-SOR, XYL, L-MA, L-TA, ICIT, and n = 100 for CA). All *cis*-diols were measured using MspA-PBA in a 1.5 M KCl buffer. A bias of +160 mV was continually applied. The final concentration of each *cis*-diol was set as described in **Methods**.

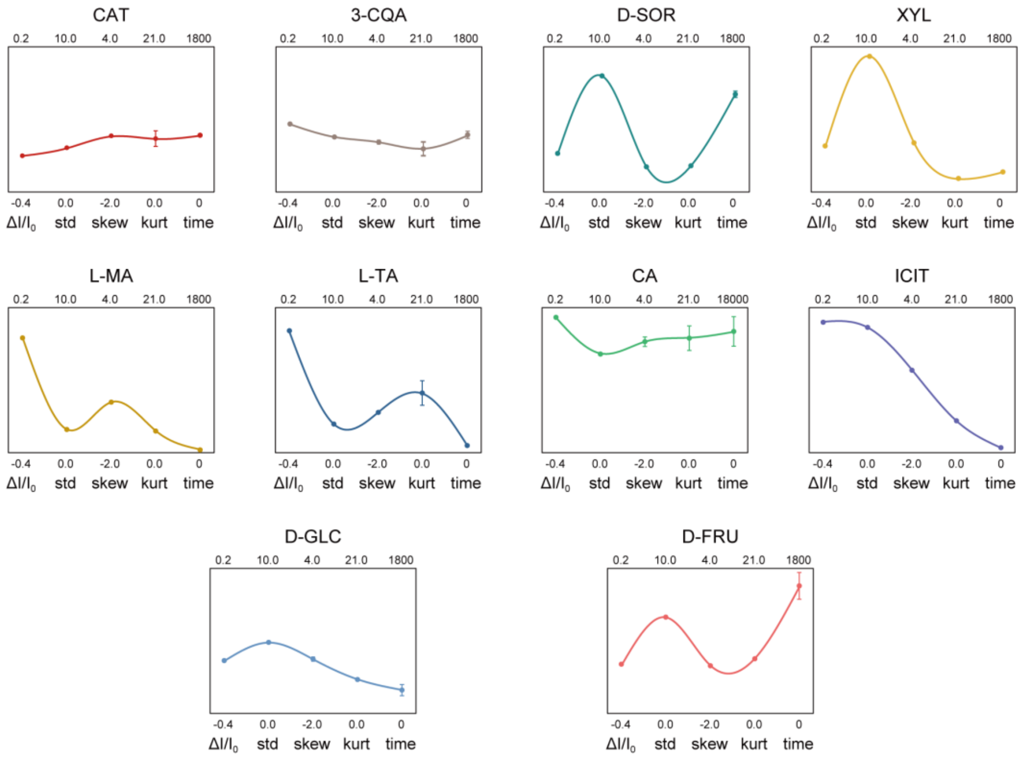

Supplementary Figure 26. Multi-feature nanopore spectrums of ten different analytes. A multi-feature nanopore spectrum provides a direct comparison of core event features between different analytes. The data in each multi-feature nanopore spectrum are the mean and standard deviations of the corresponding event parameter acquired from three independent measurements and five event features were applied for each type of analytes. Please refer to results summarized in **Supplementary Table 2** for more details. The curves were generated using the spline connected function of Origin. This side by side comparison of multi-feature spectrum clearly demonstrates the consistency of event features when independently measured by different pores. Different analytes can thus be easily discriminated by simultaneously considering all five event features. The unit of time is ms and the unit of std is pA. The data in these plots show mean ± standard deviations derived from results of three independent measurements (N=3). The error bars represent standard deviation values.

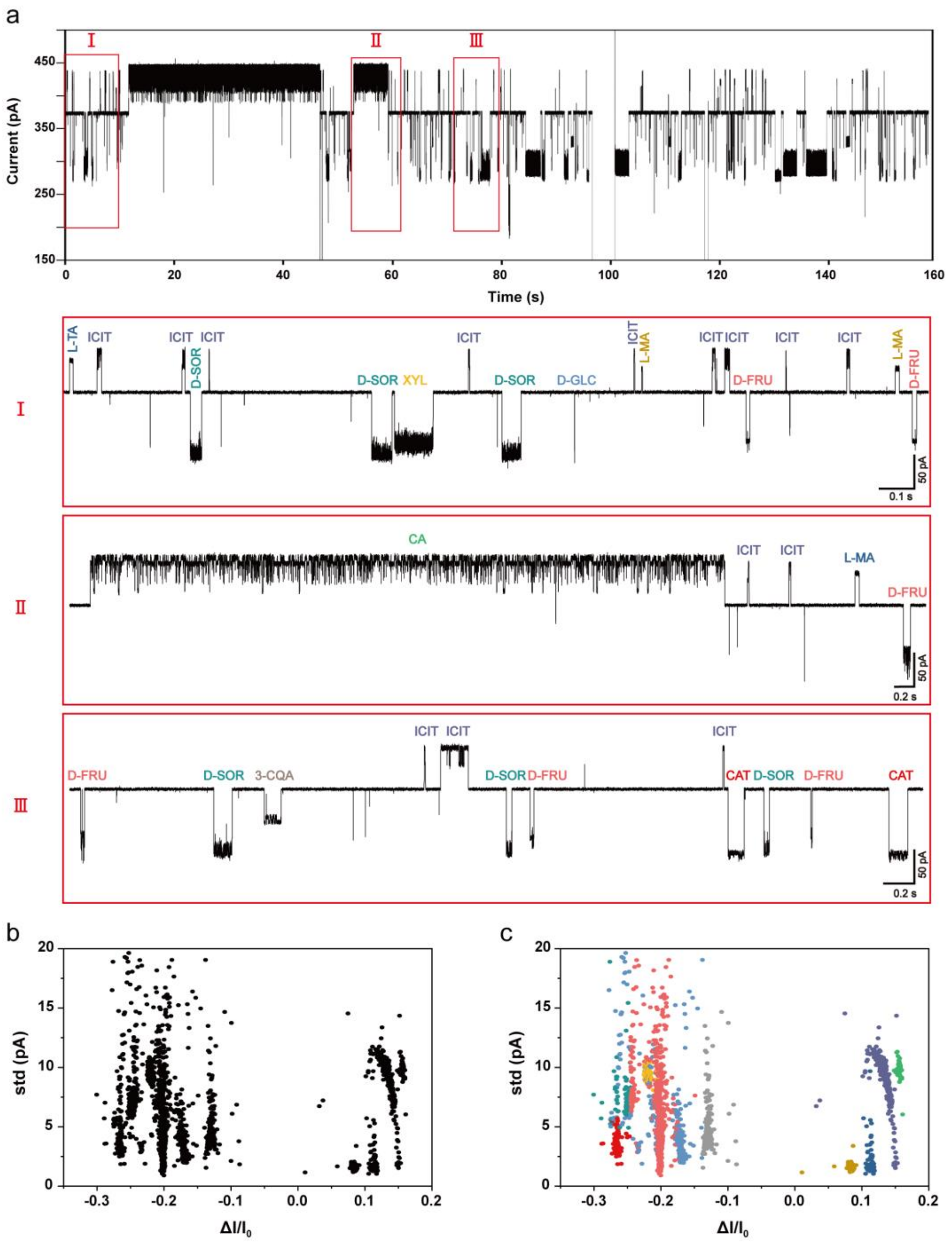

Supplementary Figure 27. Simultaneous identification of ten target *cis*-diols. (a) Representative traces acquired by simultaneous sensing of ten target *cis*-diols. A trace of 160s is shown on the top. Different sections of this trace, as marked with red boxes, were zoomed-in and demonstrated in the bottom. Events of all ten target *cis*-diols were identified by machine learning (**Fig. 3**). The measurement was carried out using MspA-PBA in a 1.5 M KCl buffer and a +160 mV bias was continually applied **(Methods)**. All *cis*-diols were added to both measurement chambers to reach the desired final concentrations. The final concentrations of CAT, 3-CQA and L-MA were 50 µM, that of D-SOR was 0.5 mM, that of XYL was 0.6 mM, that of L-TA was 0.2 mM, that of CA was 6 mM, that of ICIT was 0.1 mM, that of D-GLC was 20 mM and that of D-FRU was 4 mM. **(b)** The scatter plot of $\Delta I/I_0$ versus std of results acquired from ten *cis*-diols using the same nanopore (n=3025). Events in the scatter plot were from an 80 min continuous recorded trace. **(c)** The scatter plot of $\Delta I/I_0$ versus std of result from model prediction (n=3025). All events were predicted by the previously trained Bagging Trees model and labelled accordingly.

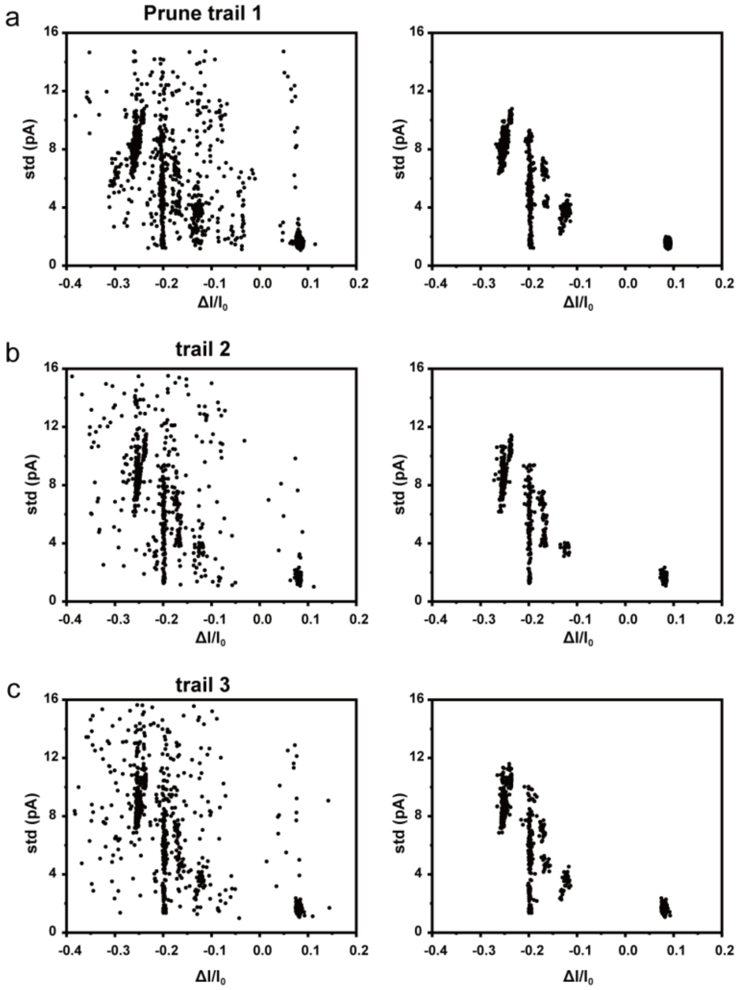

Supplementary Figure 28. Cluster analysis of prune juice. (Left) The scatter plot of *$\Delta I/I_0$* versus std for nanopore events acquired with prune juice. Three independent trails **(a-c)** under the same conditions were performed. The nanopore measurements were performed using MspA-PBA in a 1.5 M KCl buffer. A bias of +160 mV was continually applied. To both the *cis* and *trans* chambers, 5 µL prune juice was respectively added and thoroughly stirred. The data presented in each scatter plot was from a continuous measurement of 60 min. a) 2209 events, b) 1221 events and c) 1398 events were respectively included in the scatter plots. Besides the data that form clear clusters in the event distribution, some randomly distributed events were also detected. **(Right)** Events after cluster analysis treatment. The cluster analysis, which removes non-clustered data points from the scatter plot, was performed by DBSCAN. The epsilon was set to 0.17 and the min_samples was set to 18. After the treatment, a) 1770 events, b) 1021 events and c) 1151 events were respectively maintained.

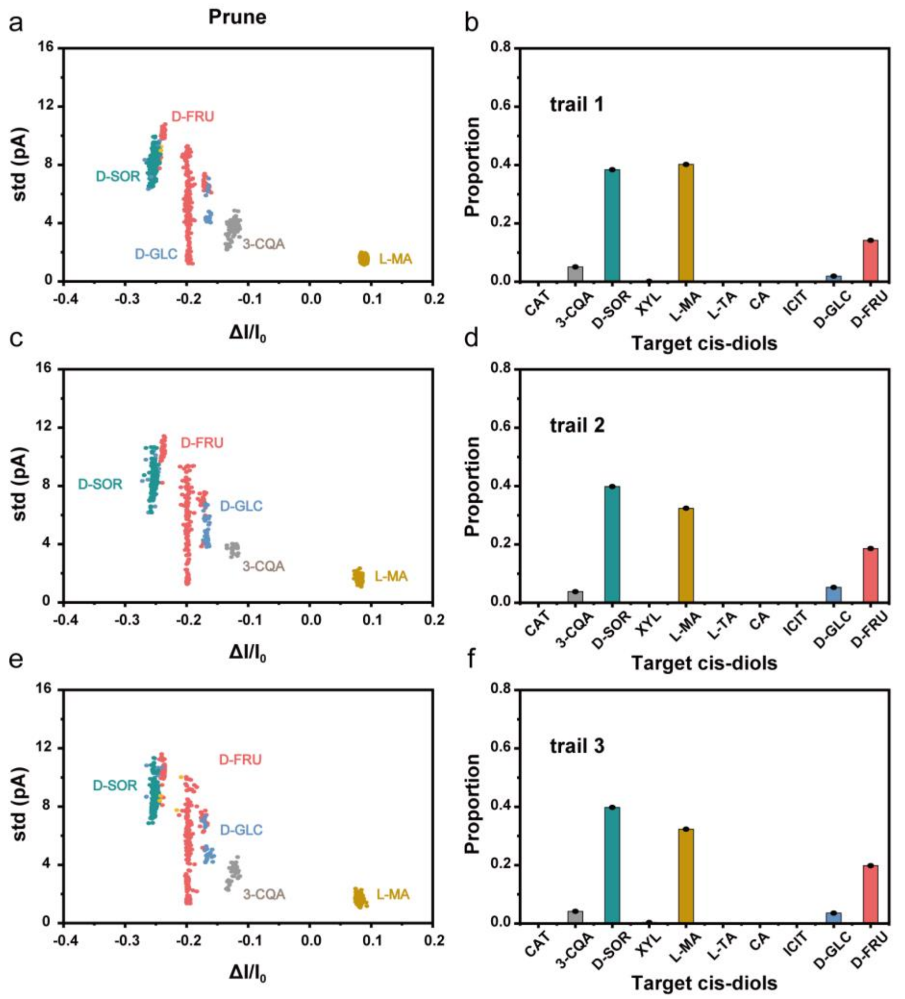

Supplementary Figure 29. Results of prune juice sensing. (a, c, e) The scatter plot of *ΔI/I₀* versus std for nanopore events acquired with prune juice. All background events were removed by DBSCAN cluster analysis (**Supplementary Fig. 28**). All events were predicted and labeled by the previously trained Bagging Trees model (**Fig. 3**). The data presented in each scatter plot was from a continuous measurement of 60 min. **a)** 1770 events, **c)** 1021 events and **e)** 1151 events were respectively included in each scatter plot. **(b, d, f)** The proportion of target *cis*-diols events in each corresponding set of scatter plot data. Results of all three independent trails showed the consistency of the measurements. The nanopore measurements were performed using MspA-PBA in a 1.5 M KCl buffer. A bias of +160 mV was continually applied. To both the *cis* and *trans* chambers, 5 µL prune juice was respectively added and thoroughly stirred. Data presented in each bar plot were from one measurements (N=1).

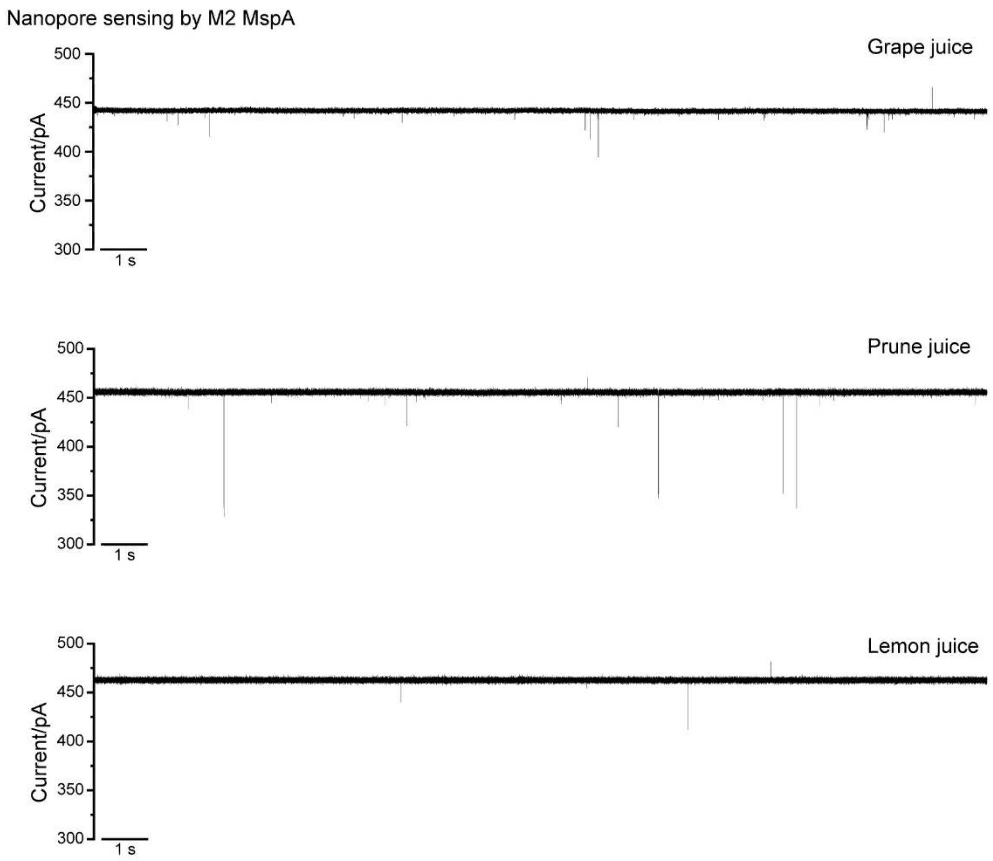

Supplementary Figure 30. Sensing of natural fruit juice using M2 MspA. The nanopore measurements were performed using M2 MspA in a 1.5 M KCl buffer. A bias of +160 mV was continually applied. In each measurement, 15 μL fruit juice was respectively added to both the *cis* and the *trans* chambers. No binding events were observed during the nanopore measurements, confirming that the phenylboronic acid modification placed at the pore constriction is critical in the detection of *cis*-diols in fruit juice.

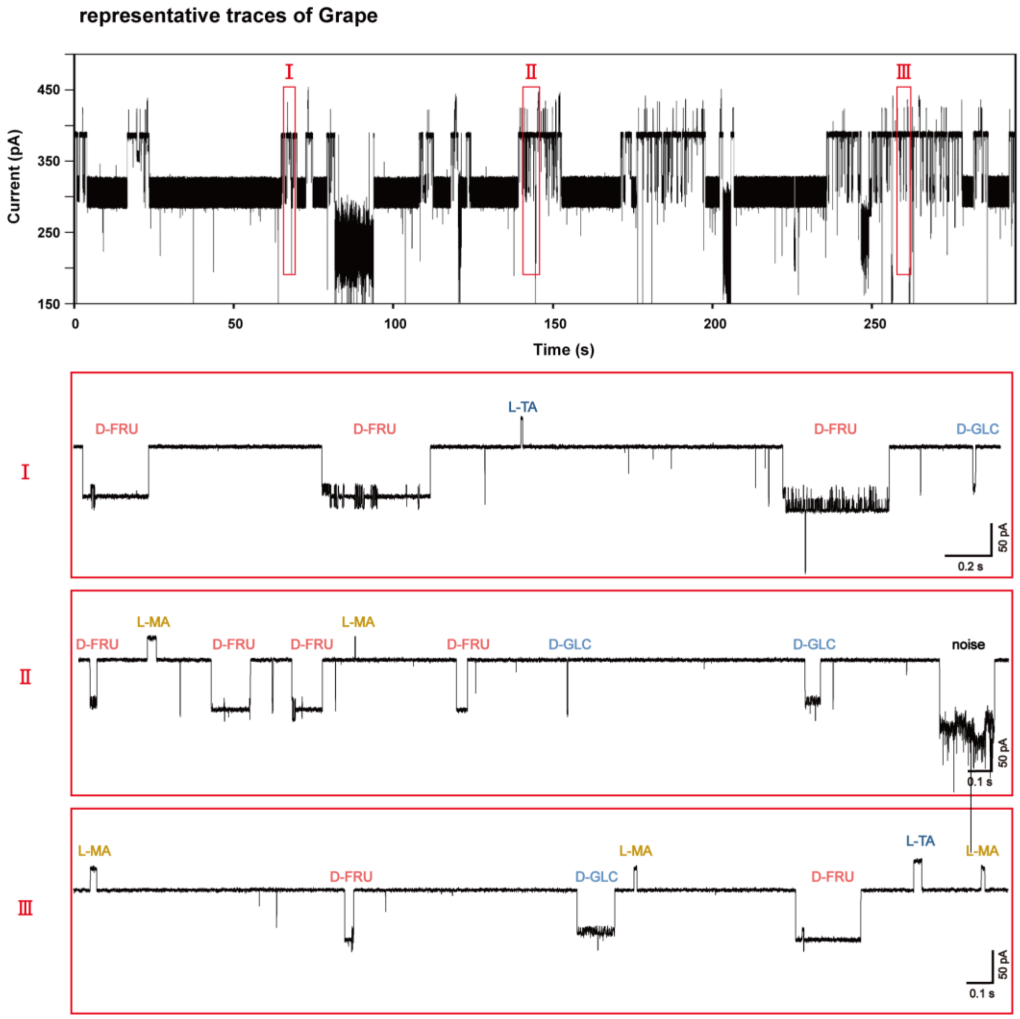

Supplementary Figure 31. Nanopore analysis of grape juice. The nanopore measurements were performed using MspA-PBA in a 1.5 M KCl buffer. A bias of +160 mV was continually applied. 5 μL grape juice was respectively added to both measurement chambers. The top figure demonstrates a 300 s segment of representative trace acquired at this condition. For a better demonstration of event details, different sections of the trace, as marked with red boxes in the top figure, were respectively demonstrated in the bottom. By machine learning prediction using the previously trained Bagging Trees model (**Fig. 3**), events of L-MA, L-TA, D-GLC and D-FRU were clearly identified. Randomly appearing noise events, which don't show a consistent event feature, were also detected and labeled on the trace.

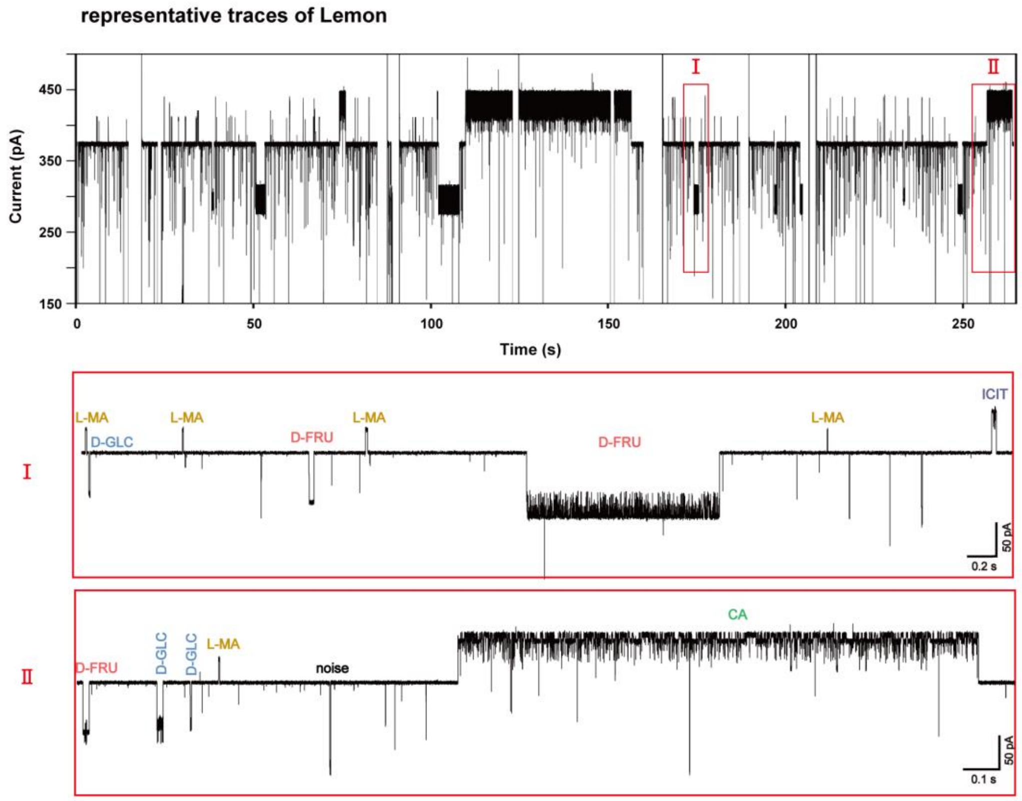

Supplementary Figure 32. Nanopore analysis of lemon juice. The nanopore measurements were performed using MspA-PBA in a 1.5 M KCl buffer. A bias of +160 mV was continually applied. 15 μL lemon juice was respectively added to both measurement chambers. The top figure demonstrates a 260 s segment of representative trace acquired at this condition. For a better demonstration of event details, different sections of the trace, as marked with red boxes in the top figure, were respectively demonstrated in the bottom. By machine learning prediction using the previously trained Bagging Trees model (**Fig. 3**), events of L-MA, CA, ICIT, D-GLC and D-FRU were clearly identified from the lemon juice. Randomly appearing noise events, which don't show a consistent event feature, were also detected and labeled on the trace.

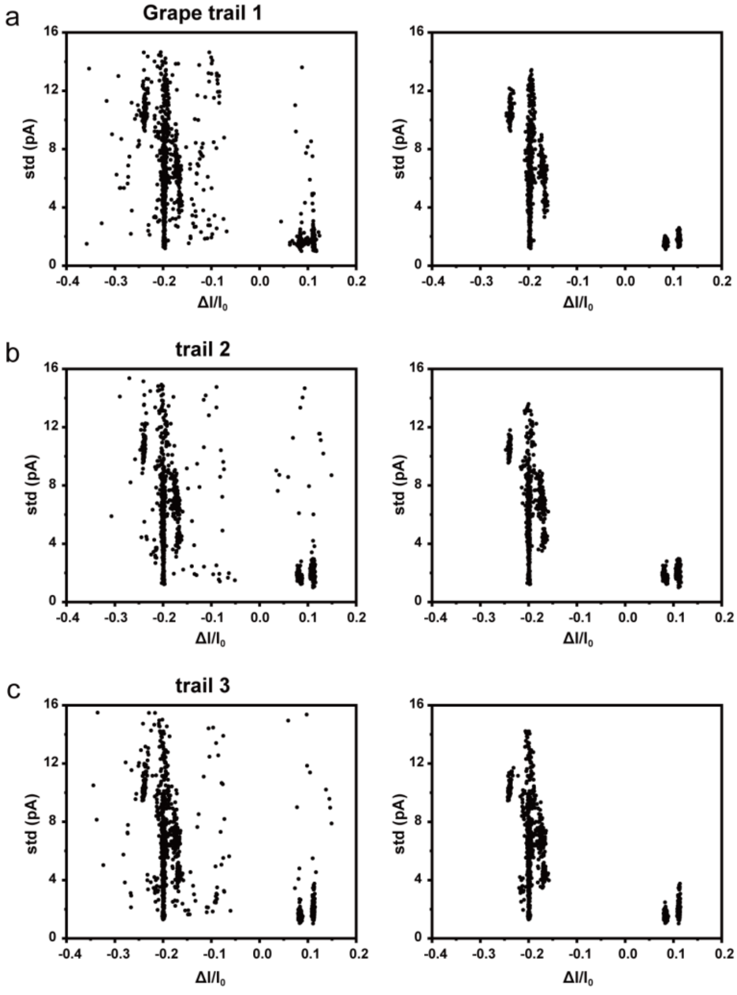

Supplementary Figure 33. Cluster analysis of grape juice. (Left) The scatter plot of *$\Delta I/I_0$* versus std for nanopore events acquired with grape juice. Three independent trails **(a-c)** under the same conditions were performed. The nanopore measurements were performed using MspA-PBA in a 1.5 M KCl buffer. A bias of +160 mV was continually applied. To both the *cis* and *trans* chambers, 5 µL grape juice was respectively added and thoroughly stirred. The data presented in each scatter plot was from a continuous measurement of 60 min. a) 1981 events, b) 1508 events and c) 2134 events were respectively included in the scatter plots. Besides the data that form clear clusters in the event distribution, some randomly distributed events were also detected. (**Right)** Events after cluster analysis treatment. The cluster analysis, which removes non-clustered data points from the scatter plot, was performed by DBSCAN. The epsilon was set to 0.17 and the min_samples was set to 18. After the treatment, a) 1732 events, b) 1396 events and c) 2017 events were respectively maintained.

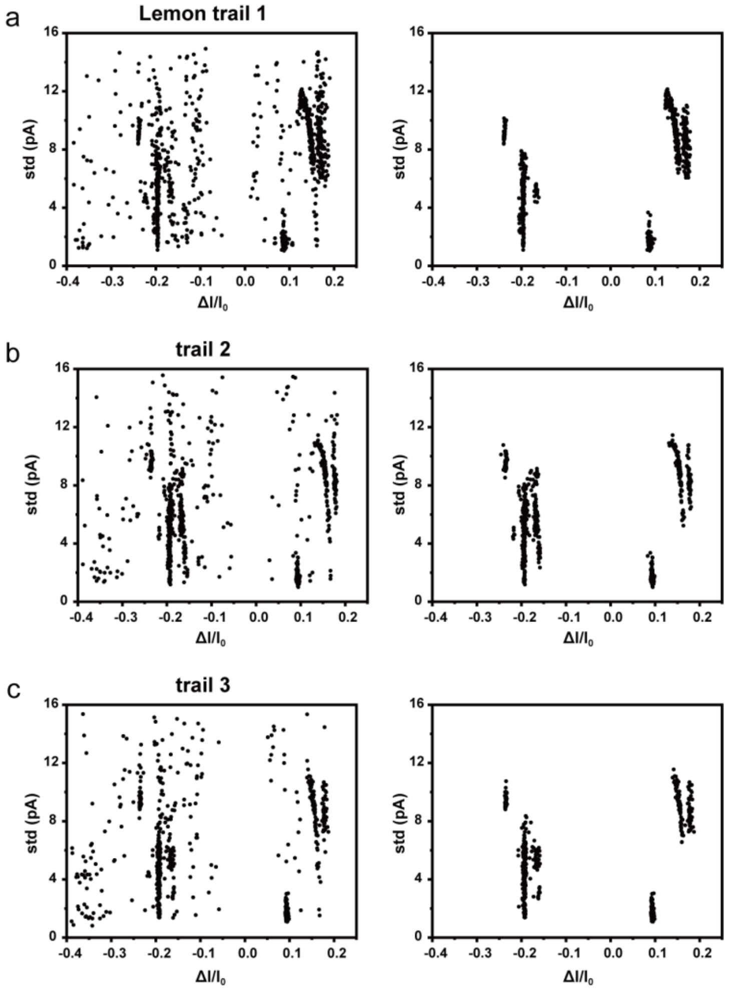

Supplementary Figure 34. Cluster analysis of lemon juice. (Left) The scatter plot of *$\Delta I/I_0$* versus std for nanopore events acquired with lemon juice. Three independent trails **(a-c)** under the same conditions were performed. The nanopore measurements were performed using MspA-PBA in a 1.5 M KCl buffer. A bias of +160 mV was continually applied. To both the *cis* and *trans* chambers, 15 µL lemon juice was respectively added and thoroughly stirred. The data presented in each scatter plot was from a continuous measurement of 60 min. a) 1328 events, b) 1191 events and c) 971 events were respectively included in the scatter plots. Besides the data that form clear clusters in the event distribution, some randomly distributed events were also detected. (**Right)** Events after cluster analysis treatment. The cluster analysis, which removes non-clustered data points from the scatter plot, was performed by DBSCAN. The epsilon was set to 0.17 and the min_samples was set to 18. After the treatment, a) 957 events, b) 997 events and c) 769 events were respectively maintained.

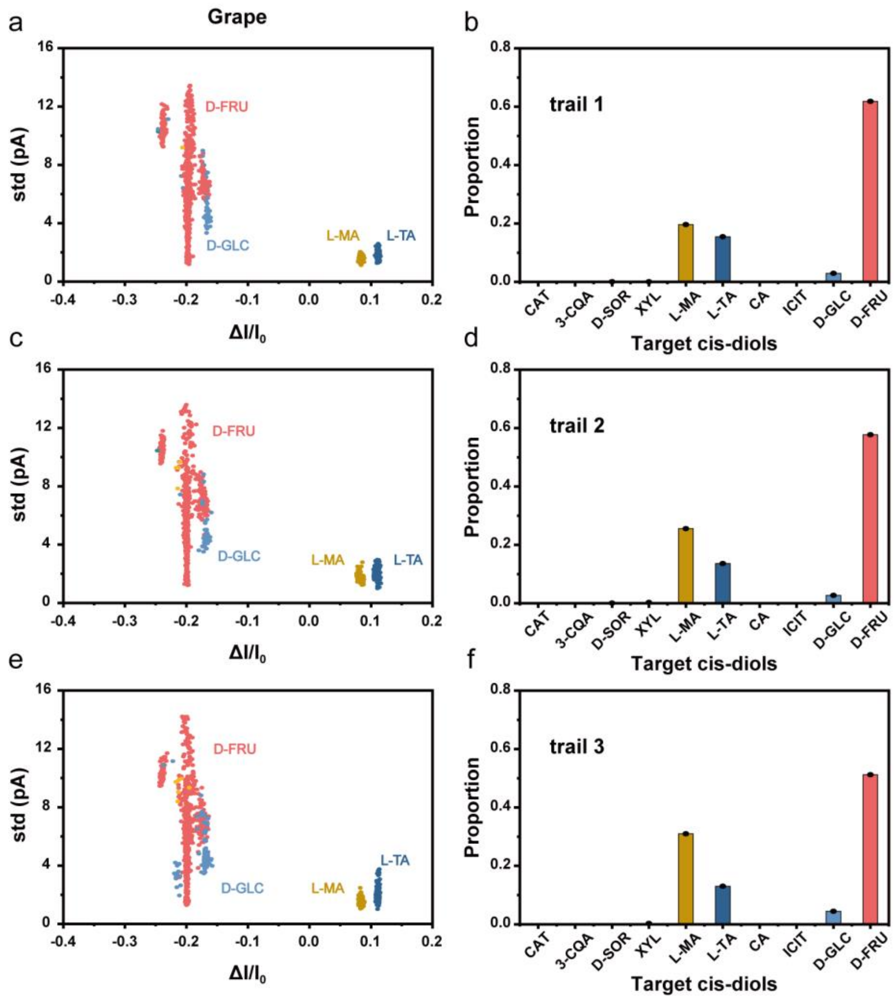

Supplementary Figure 35. Results of grape juice sensing. (a, c, e) The scatter plot of $\Delta I/I_0$ versus std for nanopore events acquired with grape juice. All background events were removed by DBSCAN cluster analysis (**Supplementary Fig. 33**). All events were predicted and labeled by the previously trained Bagging Trees model (**Fig. 3**). The data presented in each scatter plot was from a continuous measurement of 60 min. **a)** 1732 events, **c)** 1369 events and **e)** 2017 events were respectively included in each scatter plot. **(b, d, f)** The proportion of target *cis*-diols events in each corresponding set of scatter plot data. Results of all three independent trails showed the consistency of the measurements. The nanopore measurements were performed using MspA-PBA in a 1.5 M KCl buffer. A bias of +160 mV was continually applied. To both the *cis* and *trans* chambers, 5 μL grape juice was respectively added and thoroughly stirred. Data presented in each bar plot were from one measurements (N=1).

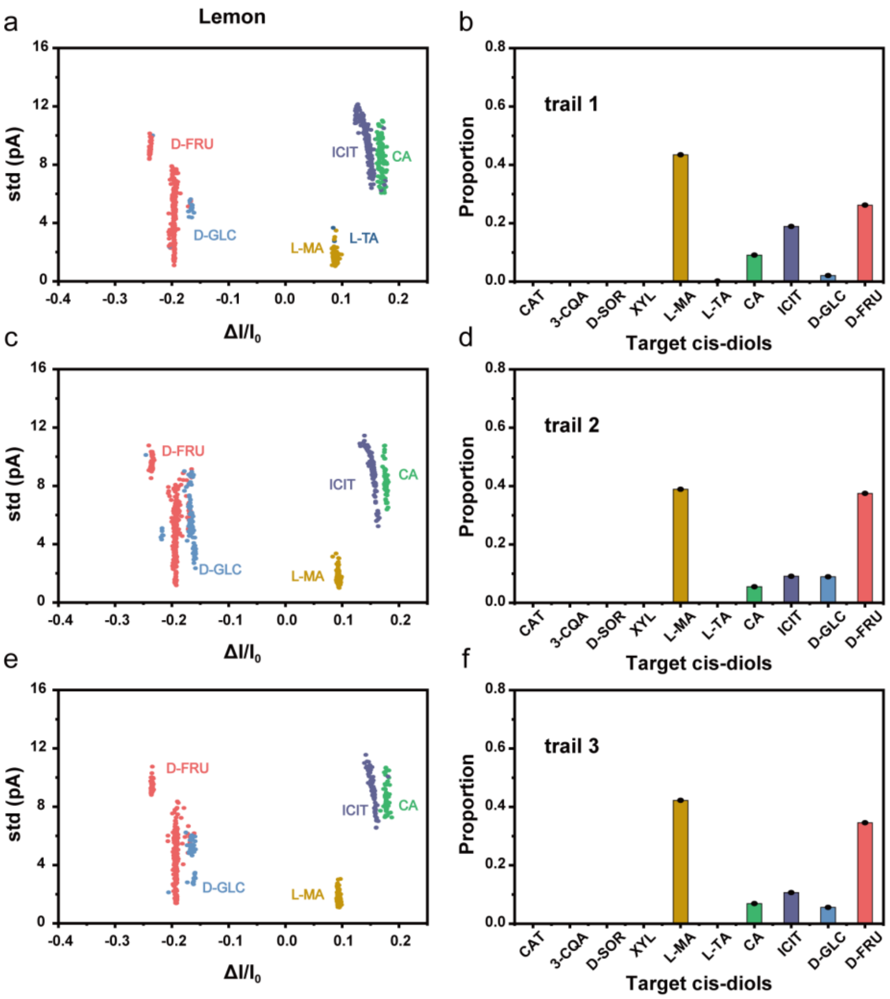

Supplementary Figure 36. Results of lemon juice sensing. (a, c, e) The scatter plot of *ΔI/I₀* versus std for nanopore events acquired with lemon juice. All background events were removed by DBSCAN cluster analysis (**Supplementary Fig. 34**). All events were predicted and labeled by the previously trained Bagging Trees model (**Fig. 3**). The data presented in each scatter plot was from a continuous measurement of 60 min. **a)** 957 events, **c)** 997 events and **e)** 769 events were respectively included in each scatter plot. **(b, d, f)** The proportion of target *cis*-diols events in each corresponding set of scatter plot data. Results of all three independent trails showed the consistency of the measurements. The nanopore measurements were performed using MspA-PBA in a 1.5 M KCl buffer. A bias of +160 mV was continually applied. To both the *cis* and *trans* chambers, 15 µL lemon juice was respectively added and thoroughly stirred. Data presented in each bar plot were from one measurements (N=1).

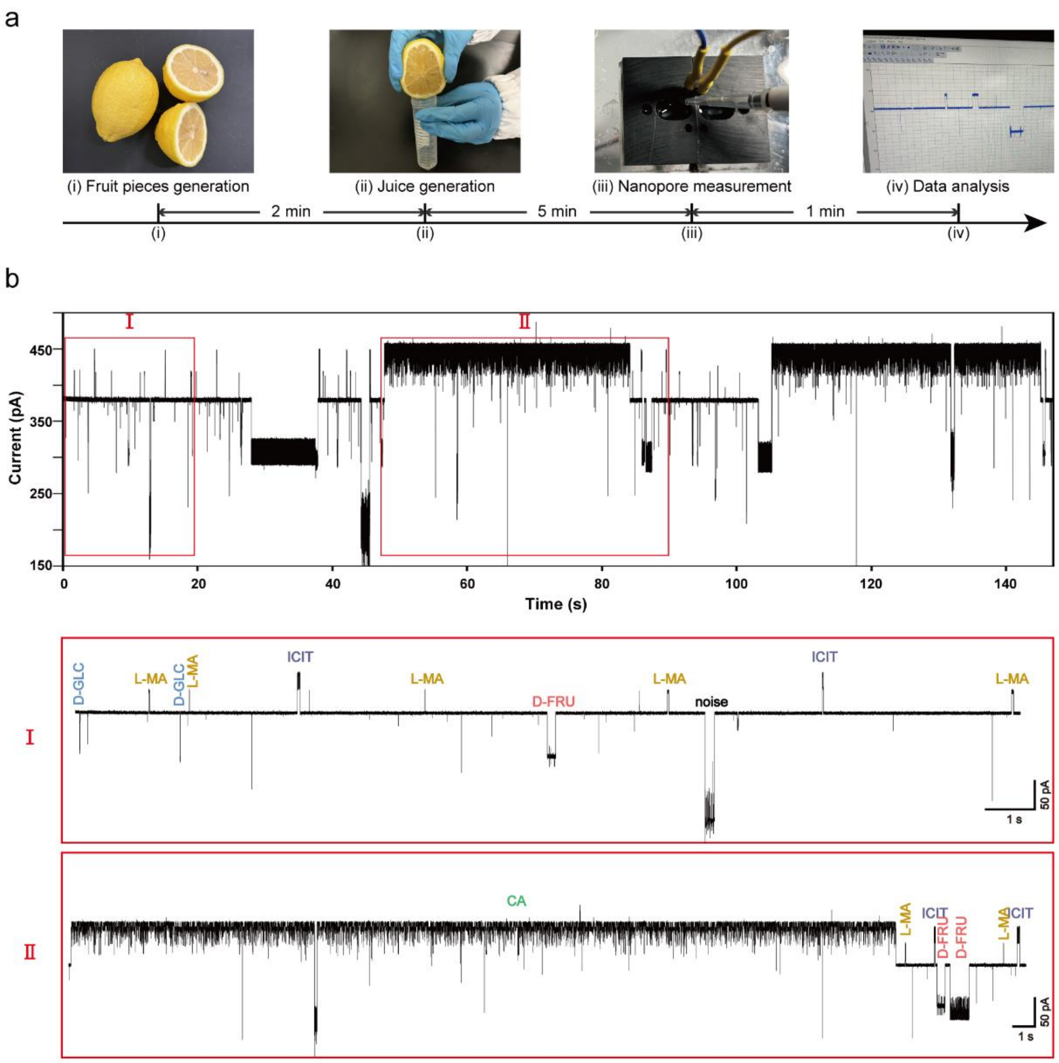

Supplementary Figure 37. Rapid analysis of natural lemon juice. (a) The workflow and the timeline of operations. (i) Fruit pieces generation. Lemons were first cut into pieces. (ii) Juice generation. The fruit pieces were hand squeezed to generate fruit juice. The juice was further treated by ultrafiltration to collect the filtrate. (iii) Nanopore measurement. The filtrate was loaded to both *cis* and *trans* chambers to initiate the measurement. (iv) Data analysis. The corresponding nanopore events were acquired and further analyzed. The nanopore measurements were performed in a 1.5 M KCl buffer with a continually applied bias of +160 mV. 15 µL lemon juice was added to both measurement chambers and thoroughly stirred. **(b)** Representative traces acquired with lemon juice. Events of L-MA, CA, ICIT, D-GLC and D-FRU can be identified by machine learning.

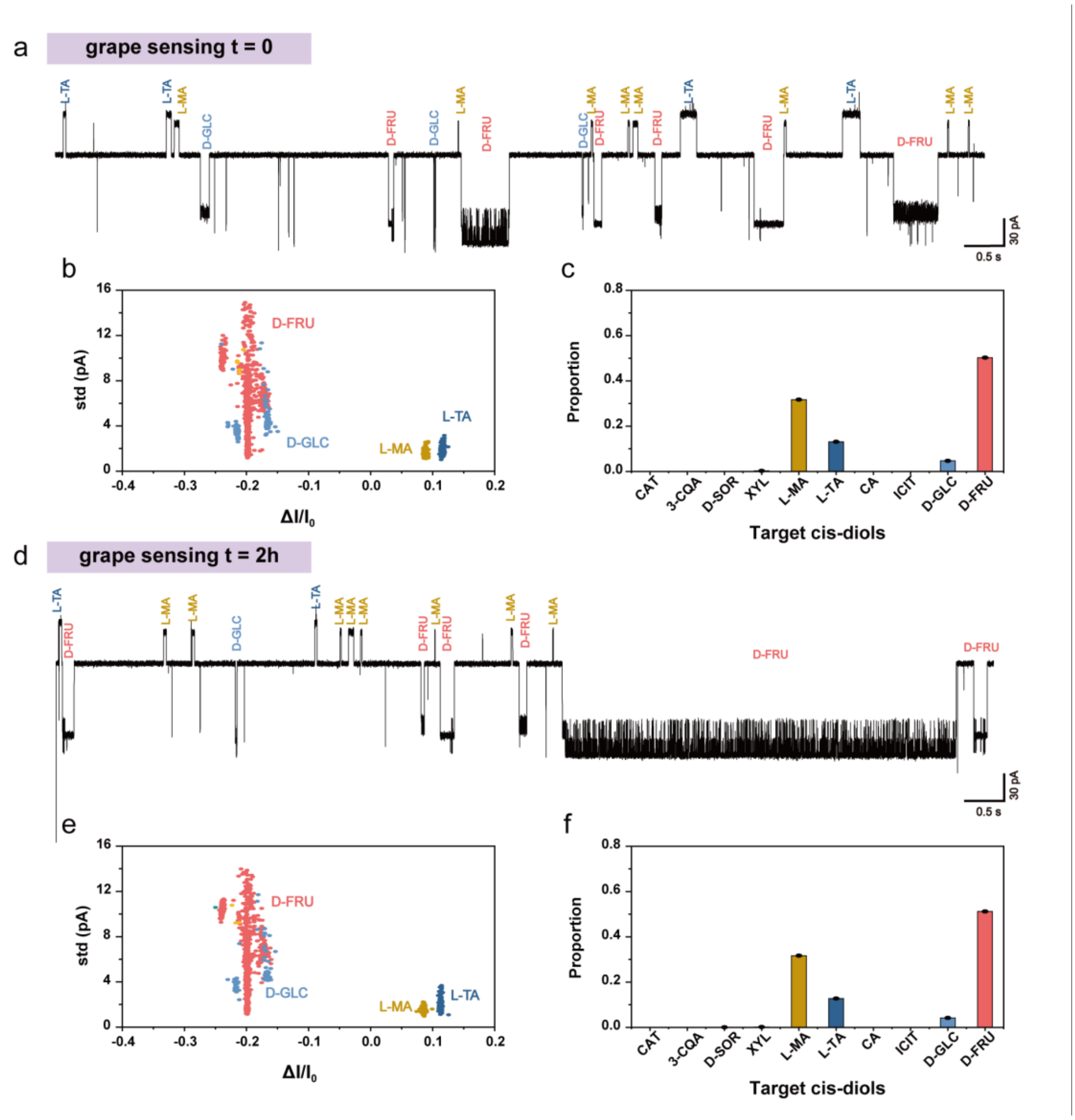

Supplementary Figure 38. Time-extended nanopore measurements. (a, d) Representative traces acquired at the **(a)** beginning and **(d)** two hours after the addition of natural grape juice. The nanopore measurements were performed using MspA-PBA in a 1.5 M KCl buffer. A bias of +160 mV was continually applied. To both the *cis* and *trans* chambers, 5 µL grape juice was respectively added and thoroughly stirred. By machine learning prediction using the previously trained model (**Fig. 3**), events of L-MA, L-TA, D-GLC and D-FRU were all clearly identified from grape juice for both conditions. Representative events and the current baseline did not change during time-extended measurements, demonstrating the stability and consistency of this technique. **(b, e)** The scatter plot of $\Delta I/I_0$ versus std for nanopore events acquired with grape juice. All background events were removed by DBSCAN cluster analysis (**Supplementary Fig. 39**). All events were predicted by the previously trained Bagging Trees model (**Fig. 3**). The data presented in each scatter plot was from a continuous measurement of 60 min. **b)** 2053 events and **e)** 1822 events were respectively included in each plot. **(c, f)** The proportion of target *cis*-diols events in each corresponding set of scatter plot data. The events proportions measured in both conditions also showed a high consistency. Data presented in each bar plot were from one measurements (N=1).

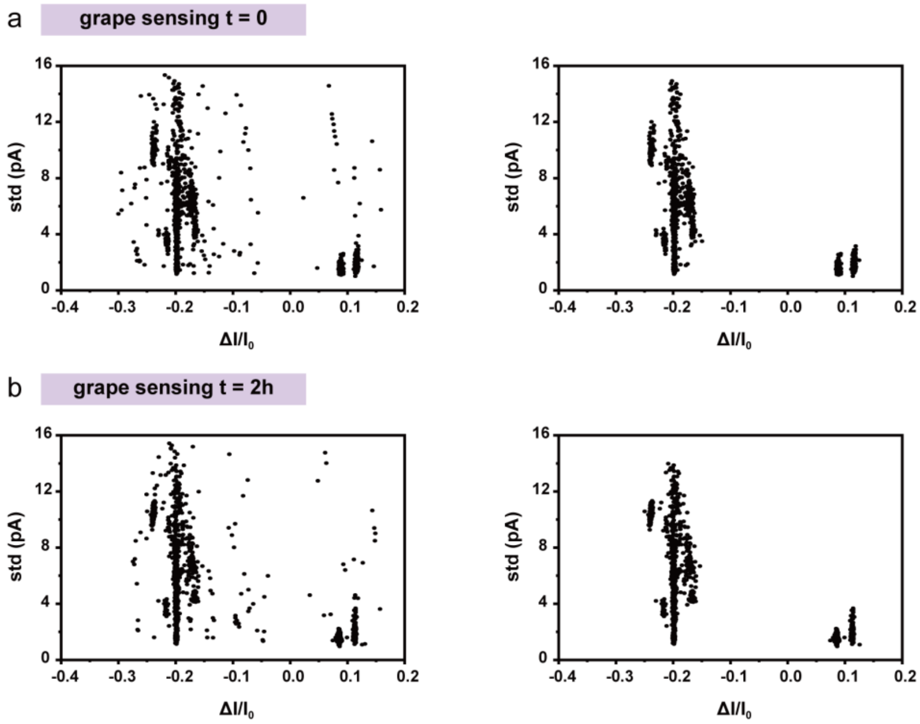

Supplementary Figure 39. Cluster analysis of grape juice with long-time nanopore sensing. (Left) The scatter plot of $\Delta I/I_0$ versus std for nanopore events acquired at the **(a)** beginning and (**b**) two hours after the grape juice addition. The nanopore measurements were performed using MspA-PBA in a 1.5 M KCl buffer. A bias of +160 mV was continually applied. To both the *cis* and *trans* chambers, 5 µL grape juice was respectively added and thoroughly stirred. The data presented in each scatter plot was from a continuous measurement of 60 min. a) 2142 events and b) 1911 events were respectively included in the scatter plots. Besides the data that form clear clusters in the event distribution, some randomly distributed events were also detected. (**Right**) Events after cluster analysis treatment. The cluster analysis, which removes non-clustered data points from the scatter plot, was performed by DBSCAN. The epsilon was set to 0.17 and the min_samples was set to 18. After the treatment, a) 2053 events and b) 1822 events were respectively retained.

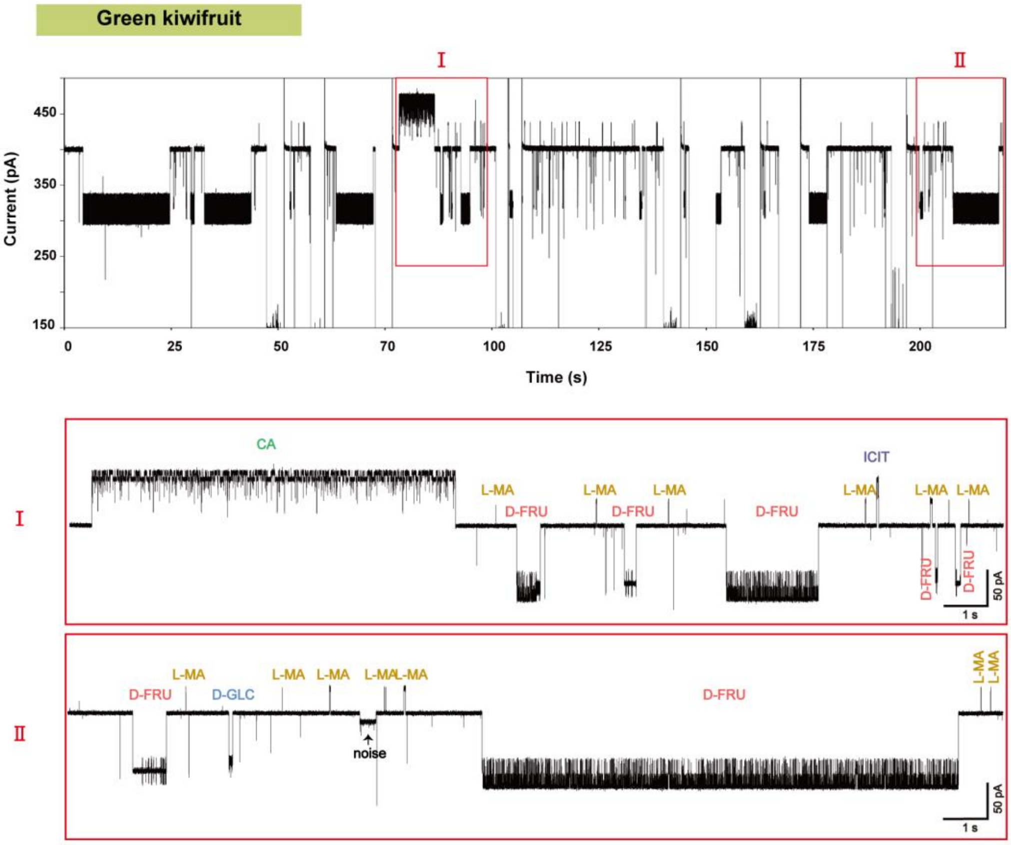

Supplementary Figure 40. Nanopore analysis of Green kiwifruit juice. The nanopore measurements were performed using MspA-PBA in a 1.5 M KCl buffer. A bias of +160 mV was continually applied. 2 μL Green kiwifruit juice was respectively added to both measurement chambers. A representative trace segment of 220 s acquired with Green kiwifruit juice is presented in the top. For a better demonstration of event details, different sections of the trace, as marked with red boxes, were respectively demonstrated in the bottom. By machine learning prediction using the previously trained Bagging Trees model (**Fig. 3**), events of L-MA, CA, ICIT, D-GLC and D-FRU were clearly identified. Randomly appearing noise events, which don't show a consistent event feature, were also detected and labeled on the trace.

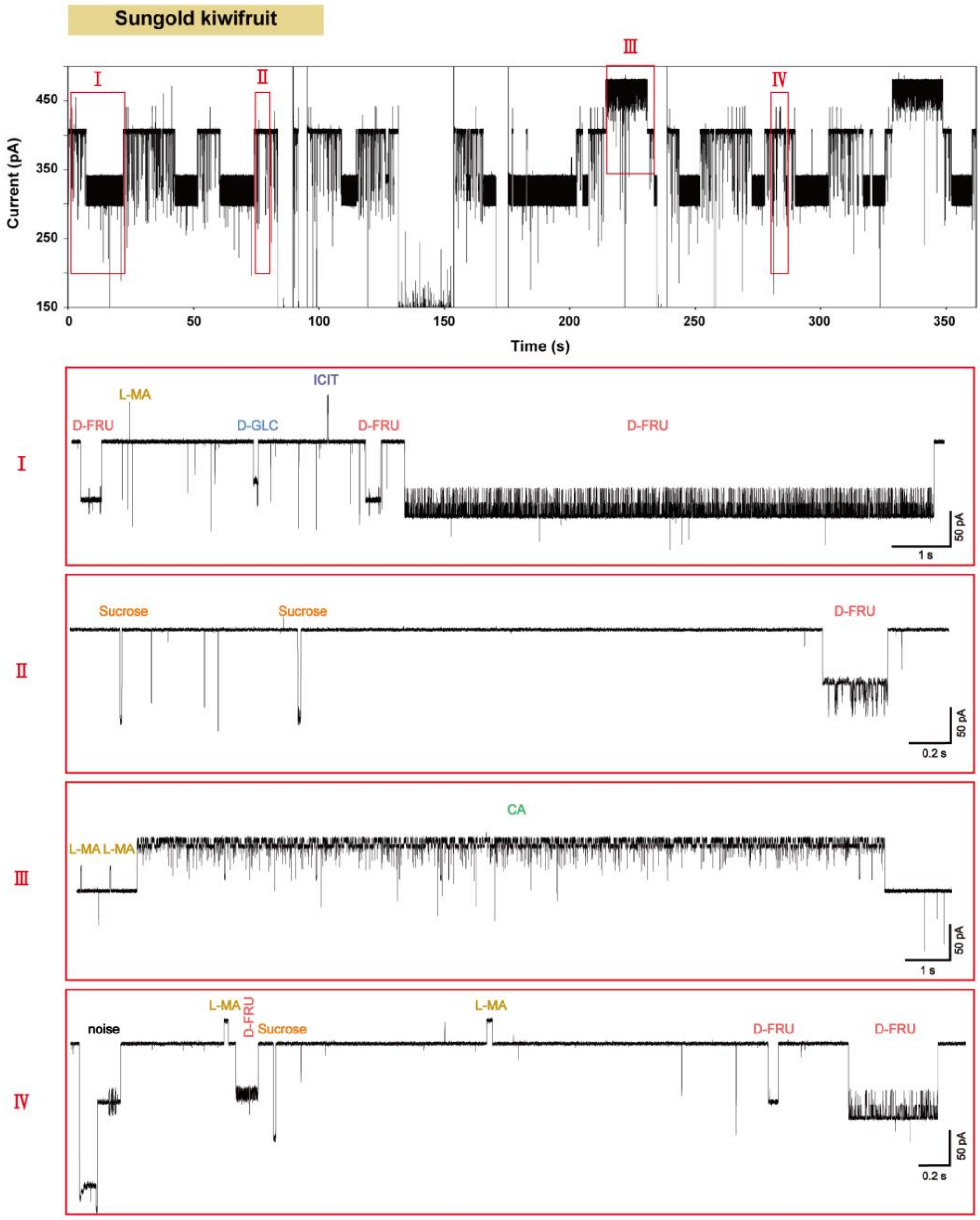

Supplementary Figure 41. Nanopore analysis of Sungold kiwifruit juice. The nanopore measurements were performed using MspA-PBA in a 1.5 M KCl buffer. A bias of +160 mV was continually applied. 2 μL Sungold kiwifruit juice was respectively added to both measurement chambers. A representative trace segment of 360 s acquired with Sungold kiwifruit juice is presented in the top. For a better demonstration of event details, different sections of the trace, as marked with red boxes, were respectively demonstrated in the bottom. By machine learning prediction using the previously trained Bagging Trees model (**Fig. 3**), events of L-MA, CA, ICIT, D-GLC, D-FRU and sucrose were clearly identified. Randomly appearing noise events, which don't show a consistent event feature, were also detected and labeled on the trace.

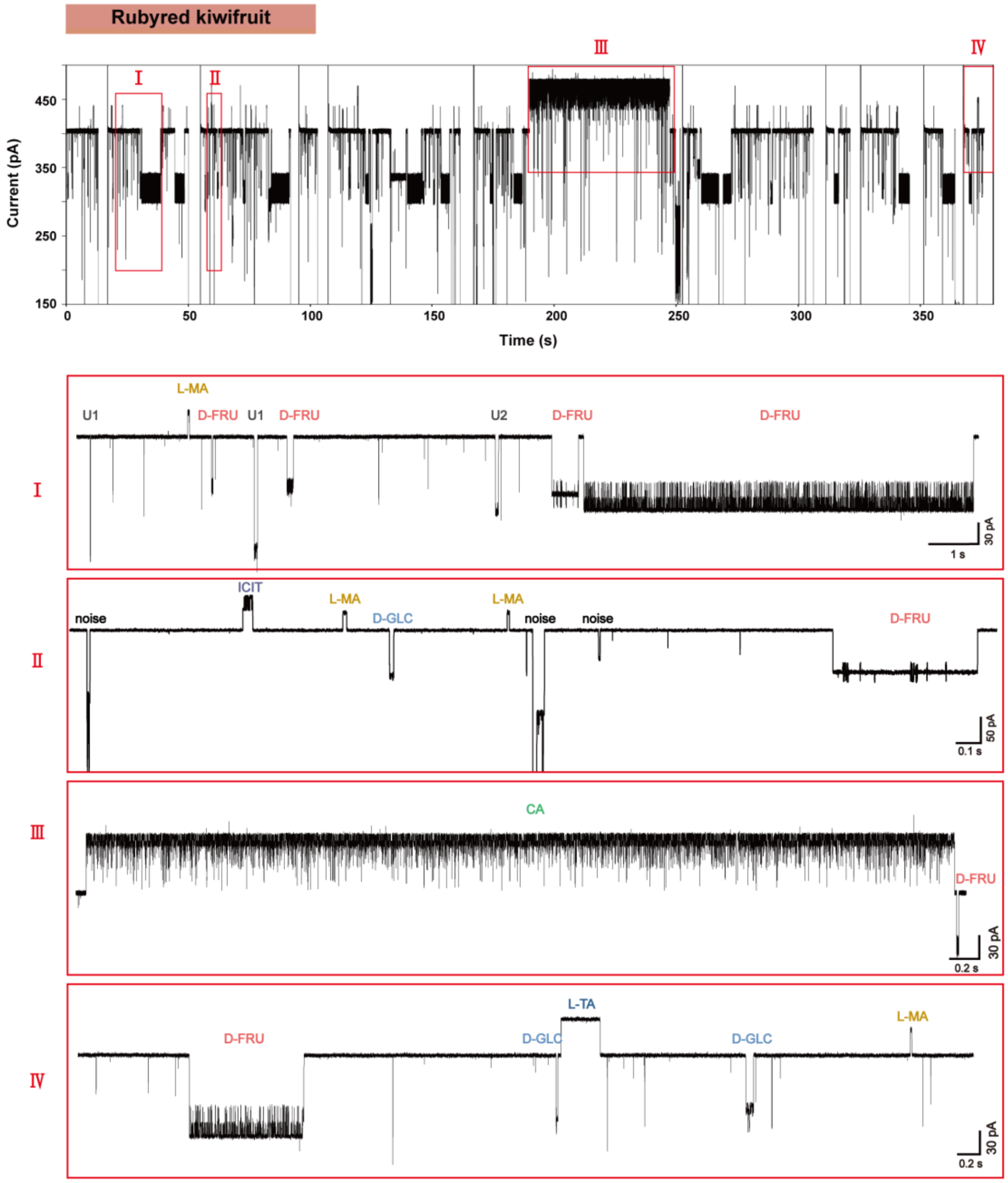

Supplementary Figure 42. Nanopore analysis of Rubyred kiwifruit juice. The nanopore measurements were performed using MspA-PBA in a 1.5 M KCl buffer. A bias of +160 mV was continually applied. 2 µL Rubyred kiwifruit juice was respectively added to both measurement chambers. A representative trace segment of 380 s acquired with Rubyred kiwifruit juice is presented in the top. For a better demonstration of event details, different sections of the trace, as marked with red boxes, were respectively demonstrated in the bottom. By machine learning prediction using the previously trained Bagging Trees model (**Fig. 3**), events of L-MA, L-TA, CA, ICIT, D-GLC, D-FRU, U1 (unidentified *cis*-diol 1) and U2 (unidentified *cis*-diol 2) were clearly identified. Randomly appearing noise events, which don't show a consistent event feature, were also detected and labeled on the trace.

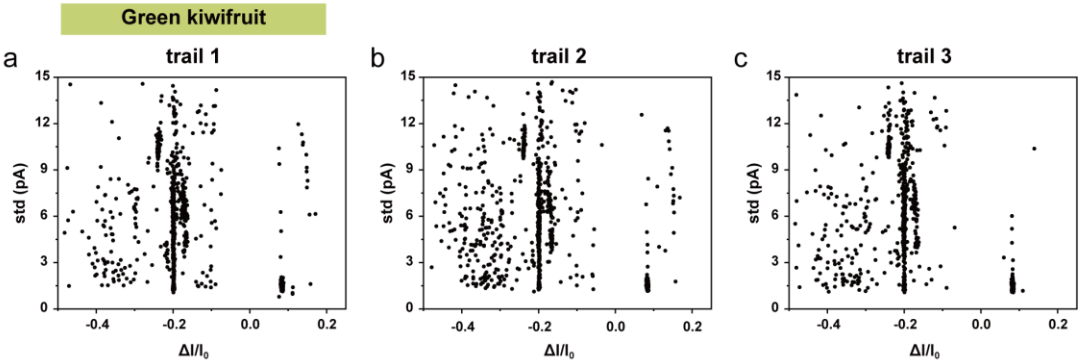

Supplementary Figure 43. Events of Green kiwifruit juice. (a-c) The scatter plots of *$\Delta I/I_0$* versus std for nanopore events acquired with Green kiwifruit juice. Results in different scatter plots were respectively acquired from three independent measurements. The nanopore measurements were performed using MspA-PBA in a 1.5 M KCl buffer. 2 μL Green kiwifruit juice was respectively added to both measurement chambers. A +160 mV bias was continually applied. Results in each scatter plot were from a 60 min continually recorded trace. a) 1175 events, b) 1124 events and c) 918 events were respectively included in each scatter plot.

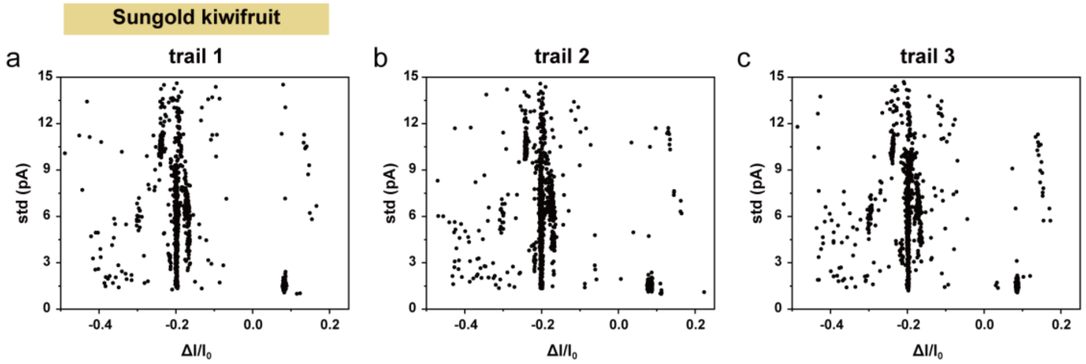

Supplementary Figure 44. Events of Sungold kiwifruit juice. (a-c) The scatter plots of *ΔI/I₀* versus std for nanopore events acquired with Sungold kiwifruit juice. Results in different scatter plots were respectively acquired from three independent measurements. The nanopore measurements were performed using MspA-PBA in a 1.5 M KCl buffer. 2 μL Sungold kiwifruit juice was respectively added to both measurement chambers. A +160 mV bias was continually applied. Results in each scatter plot were from a 60 min continually recorded trace. a) 1167 events, b) 1696 events and c) 1508 events were respectively included in each scatter plot.

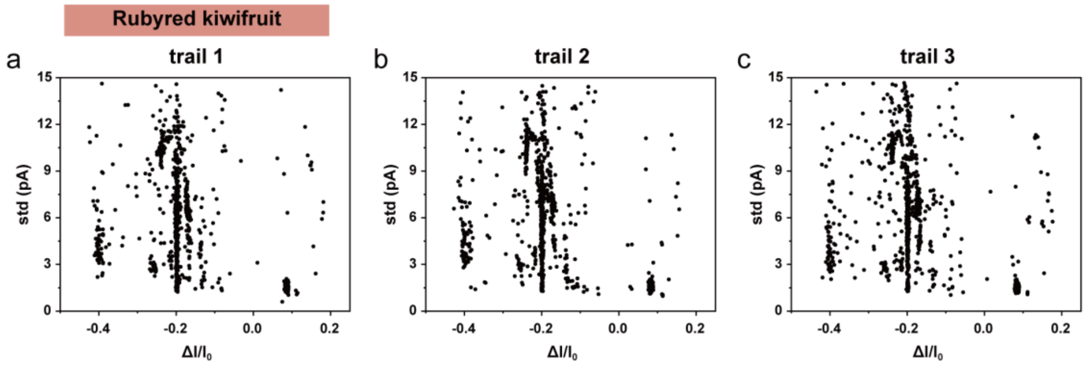

Supplementary Figure 45. Events of Rubyred kiwifruit juice. (a-c) The scatter plots of *$\Delta I/I_0$* versus std for nanopore events acquired with Rubyred kiwifruit juice. Results in different scatter plots were respectively acquired from three independent measurements. The nanopore measurements were performed using MspA-PBA in a 1.5 M KCl buffer. 2 μL Rubyred kiwifruit juice was respectively added to both measurement chambers. A +160 mV bias was continually applied. Results in each scatter plot were from a 60 min continually recorded trace. a) 1209 events, b) 1211 events and c) 1223 events were respectively included in each scatter plot.

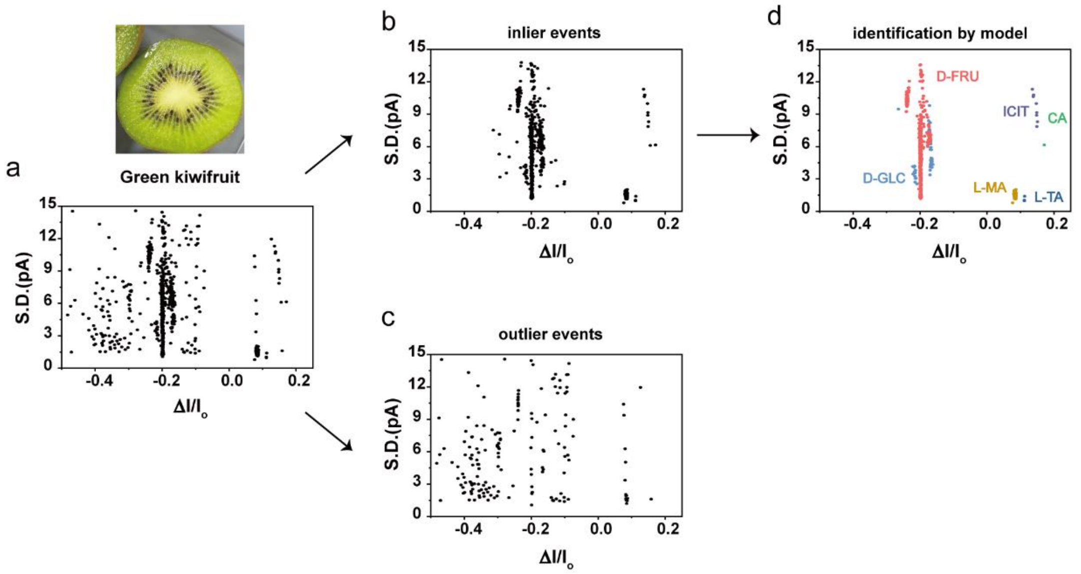

Supplementary Figure 46. The workflow of nanopore analysis of Green kiwifruit juice. (a) The scatter plot of $\Delta I/I_0$ versus std for nanopore events acquired with Green kiwifruit juice **(Supplementary Fig. 43)**. The events were first divided into **(b)** inlier events and **(c)** outlier events using the One-Class SVM algorithm, an unsupervised machine learning algorithm applied for outlier event detection. **(b)** The scatter plot of $\Delta I/I_0$ versus std for inlier events acquired with Green kiwifruit juice. **(c)** The scatter plot of $\Delta I/I_0$ versus std for outlier events acquired with Green kiwifruit juice. **(d)** The scatter plot of $\Delta I/I_0$ versus std for inlier events predicted by the previously trained Bagging Tress model (**Fig. 3**). Events of L-MA, L-TA, CA, ICIT, D-GLC and D-FRU were identified from the Green kiwifruit juice.

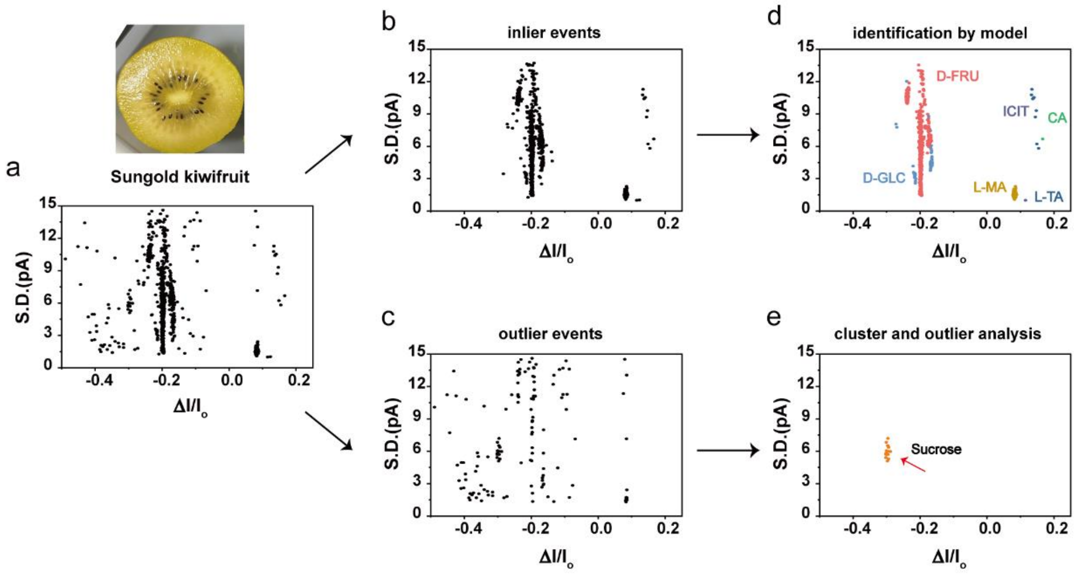

Supplementary Figure 47. The workflow of nanopore analysis of Sungold kiwifruit juice. (a) The scatter plot of $\Delta I/I_0$ versus std for nanopore events acquired with Sungold kiwifruit juice **(Supplementary Fig. 44)**. The events were first divided into **(b)** inlier events and **(c)** outlier events using the One-Class SVM algorithm, an unsupervised machine learning algorithm applied for outlier event detection. **(b)** The scatter plot of $\Delta I/I_0$ versus std for inlier events acquired with Sungold kiwifruit juice. **(c)** The scatter plot of $\Delta I/I_0$ versus std for outlier events acquired with Sungold kiwifruit juice. **(d)** The scatter plot of $\Delta I/I_0$ versus std for inlier events predicted by the previously trained Bagging Tress model (**Fig. 3**). Events of L-MA, L-TA, CA, ICIT, D-GLC and D-FRU were identified from the Sungold kiwifruit juice. **(e)** Scatter plot of $\Delta I/I_0$ versus std for cluster analysis of outlier events using DBSCAN, an unsupervised learning algorithm to identify clustered events in the outliers. The epsilon was set to 0.1 and the min_samples was set to 10. After the treatment, one cluster of events was detected. According to the comparison with the event features of sucrose (**Supplementary Fig. 49**), this cluster of events was confirmed to be sucrose (red arrow). According to previously published literatures[1,2], the presence of sucrose in Sungold kiwifruit was previously reported. We thus confirm that sucrose is successfully identified from Sungold kiwifruit solely using nanopore readout.

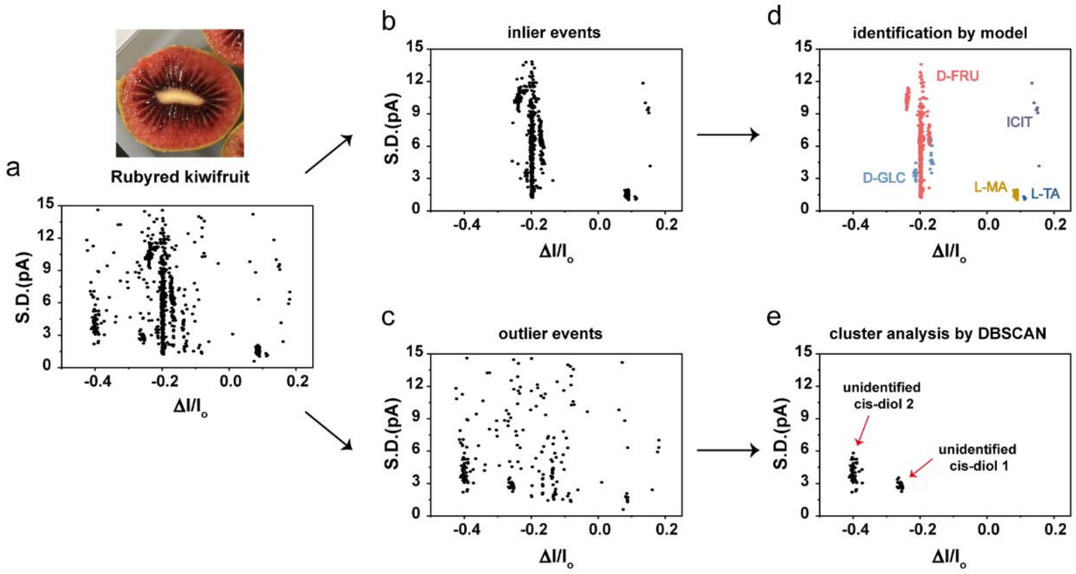

Supplementary Figure 48. The workflow of nanopore analysis of Rubyred kiwifruit juice. (a) The scatter plot of *$\Delta I/I_0$* versus std for nanopore events acquired with Rubyred kiwifruit juice **(Supplementary Fig. 45)**. The events were first divided into **(b)** inlier events and **(c)** outlier events using the One-Class SVM algorithm, an unsupervised machine learning algorithm applied for outlier event detection. **(b)** The scatter plot of *$\Delta I/I_0$* versus std for inlier events acquired with Rubyred kiwifruit juice. **(c)** The scatter plot of *$\Delta I/I_0$* versus std for outlier events acquired with Rubyred kiwifruit juice. **(d)** The scatter plot of *$\Delta I/I_0$* versus std for inlier events predicted by the previously trained Bagging Tress model (**Fig. 3**). Events of L-MA, L-TA, ICIT, D-GLC and D-FRU were identified from the Rubyred kiwifruit juice. **(e)** The scatter plot of *$\Delta I/I_0$* versus std for cluster analysis of outlier events using DBSCAN, an unsupervised learning algorithm to identify clustering events in the outliers. The epsilon was set to 0.1 and the min_samples was set to 10. After the treatment, two extra clusters of events were detected and were respectively marked as U1 (unidentified *cis*-diol 1, red arrow) and U2 (unidentified *cis*-diol 2, red arrow). Though their event features are different from any previously studied *cis*-diols using this nanopore and their identities are not known at present. We can conclude that they are however not detected in Green and Sungold kiwifruits at all. These two events, including U1 and U2, are thus uniquely identified only in the Rubyred kiwifruit, among all kiwifruits we have investigated using MspA-PBA.

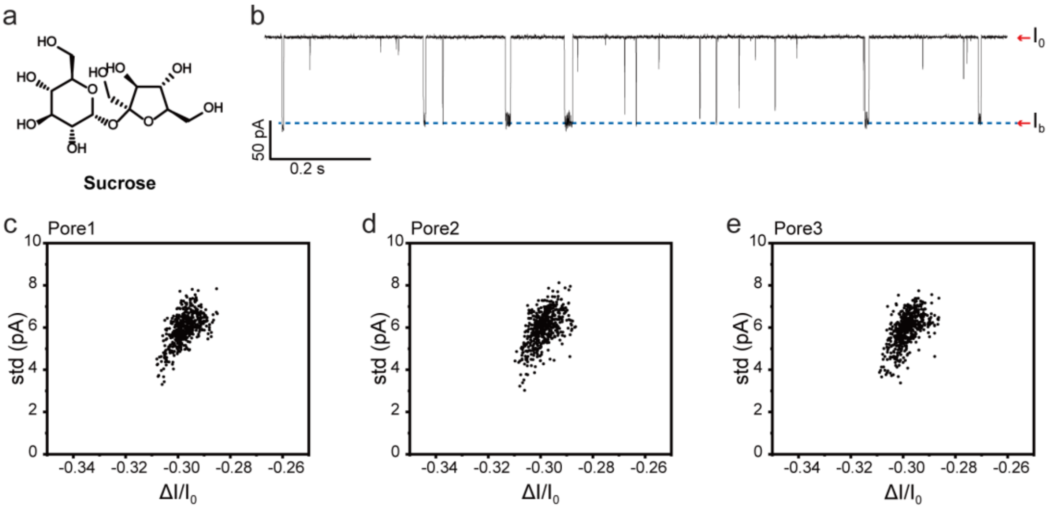

Supplementary Figure 49. Events of sucrose. (a) The chemical structure of sucrose. **(b)** Representative traces acquired with sucrose as the sole analyte. **(c-e)** Scatter plots of $\Delta I/I_0$ versus std for sucrose events. Results in different scatter plots were respectively acquired from three independent measurements. Each scatter plot contains 500 events. The nanopore measurements were performed using MspA-PBA in a 1.5 M KCl buffer. Sucrose was added to *cis* and *trans* with a final concentration of 20 mM. A +160 mV bias was continually applied.

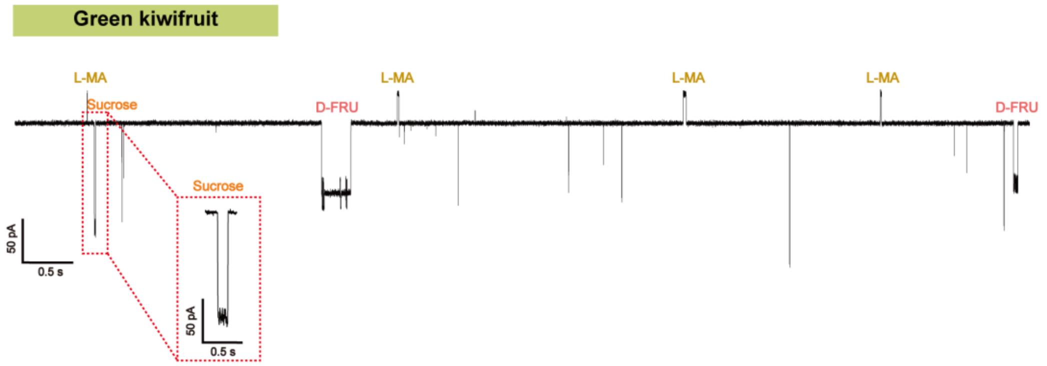

Supplementary Figure 50. Sucrose events detected in Green kiwifruit juice. The nanopore measurements were performed using MspA-PBA in a 1.5 M KCl buffer. A bias of +160 mV was continually applied. 2 μL Green kiwifruit juice was respectively added to both measurement chambers. A representative trace acquired with Green kiwifruit juice is presented. By One-Class SVM trained on the sucrose dataset (**Supplementary Fig. 49**), events of sucrose in Green kiwifruit juice were clearly identified from the Green kiwifruit juice. For the ease of demonstration, a zoom-in view of the red box marked area is demonstrated in the bottom.

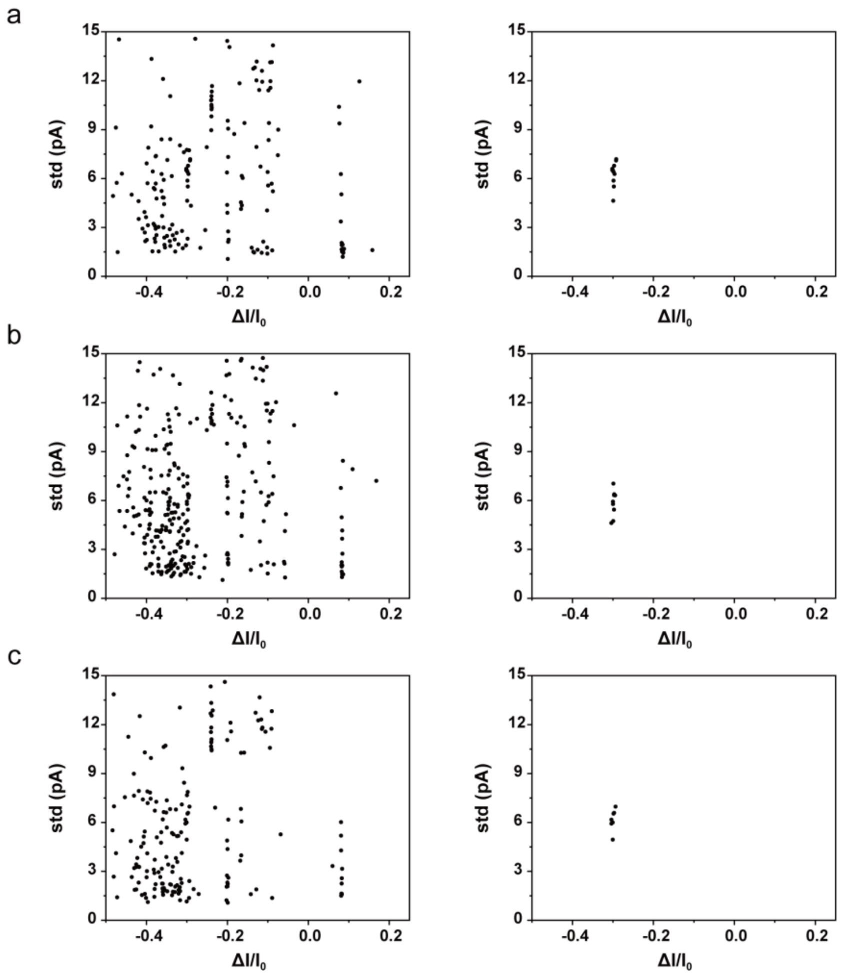

Supplementary Figure 51. Identification of sucrose in Green kiwifruit juice using One-Class SVM. (Left) The scatter plot of $\Delta I/I_0$ versus std for outlier events acquired with Green kiwifruit juice (**Supplementary Fig. 46**). Three independent measurements **(a-c)** under the same condition were performed. The nanopore measurements were performed using MspA-PBA in a 1.5 M KCl buffer. A bias of +160 mV was continually applied. To both the *cis* and *trans* chambers, 2 µL Green kiwifruit juice was respectively added and thoroughly stirred. The data presented in each scatter plot was from a continuous measurement of 60 min. a) 173 events, b) 290 events and c) 177 events were respectively included in the scatter plots. (**Right)** Events after outlier analysis treatment based on the standard sucrose data (**Supplementary Fig. 49**). The outlier analysis, which removes data clearly inconsistent to the training data from the scatter plot, was performed by One-Class SVM. After the treatment, a) 11 events, b) 9 events and c) 7 events were respectively retained in the scatter plot.

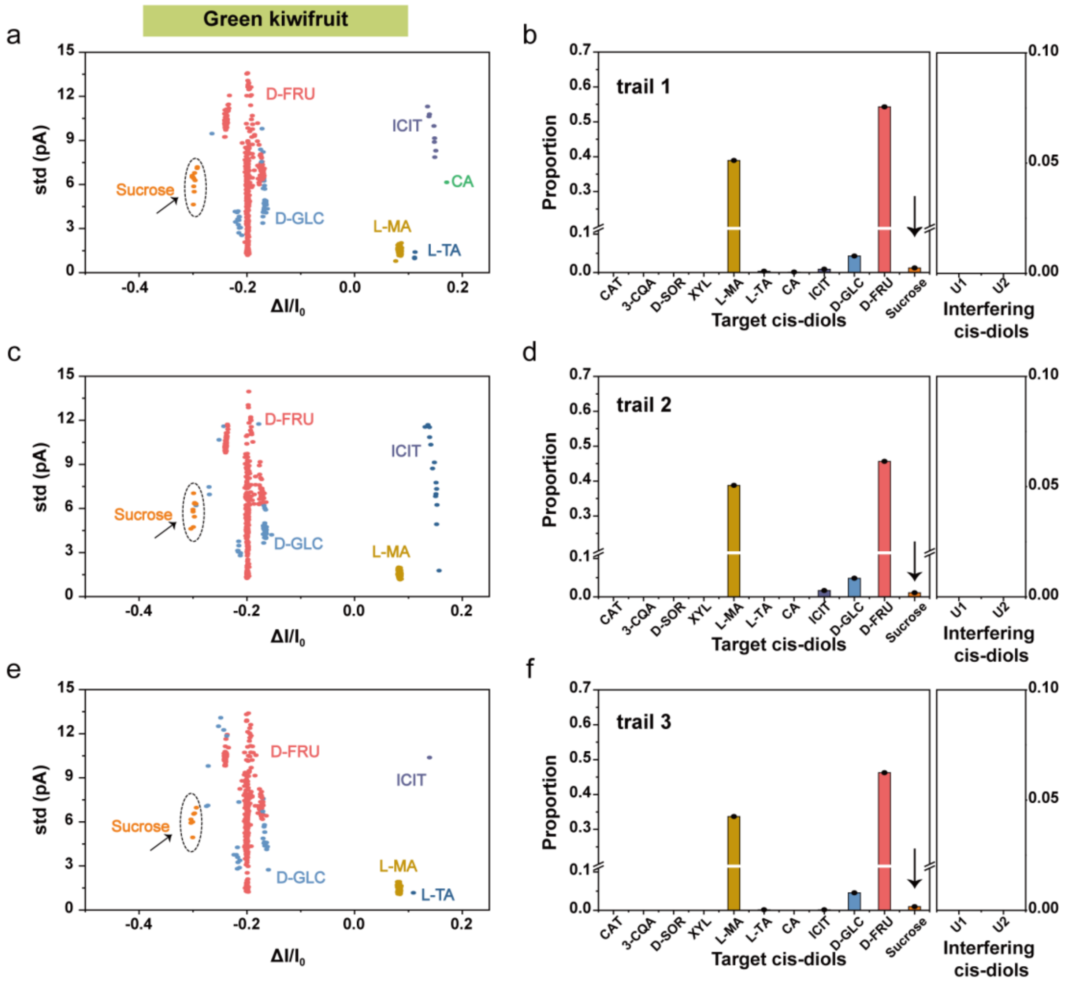

Supplementary Figure 52. Results of Green kiwifruit juice analysis. (a, c, e) The scatter plot of *ΔI/I₀* versus std for nanopore events acquired with Green kiwifruit juice. All background events were removed by cluster analysis (**Supplementary Fig. 46**) and outlier analysis (**Supplementary Fig. 51**). All events except sucrose were predicted and labeled by the previously trained Bagging Trees model (**Fig. 3**). The data presented in each scatter plot was from a continuous measurement of 60 min. **a)** 930 events, **c)** 776 events and **e)** 641 events were respectively included in each scatter plot. **(b, d, f)** The proportion of target *cis*-diol events in each corresponding set of scatter plot data. Results of all three independent measurements also demonstrate a high consistency. Data presented in each bar plot were from result acquired from one measurement (N=1). Results of three measurements were simultaneously presented to show data consistency. Results generated by sucrose events are marked with arrows. The nanopore measurements were performed using MspA-PBA in a 1.5 M KCl buffer. A bias of +160 mV was continually applied. To both the *cis* and *trans* chambers, 2 µL Green kiwifruit juice was respectively added and thoroughly stirred.

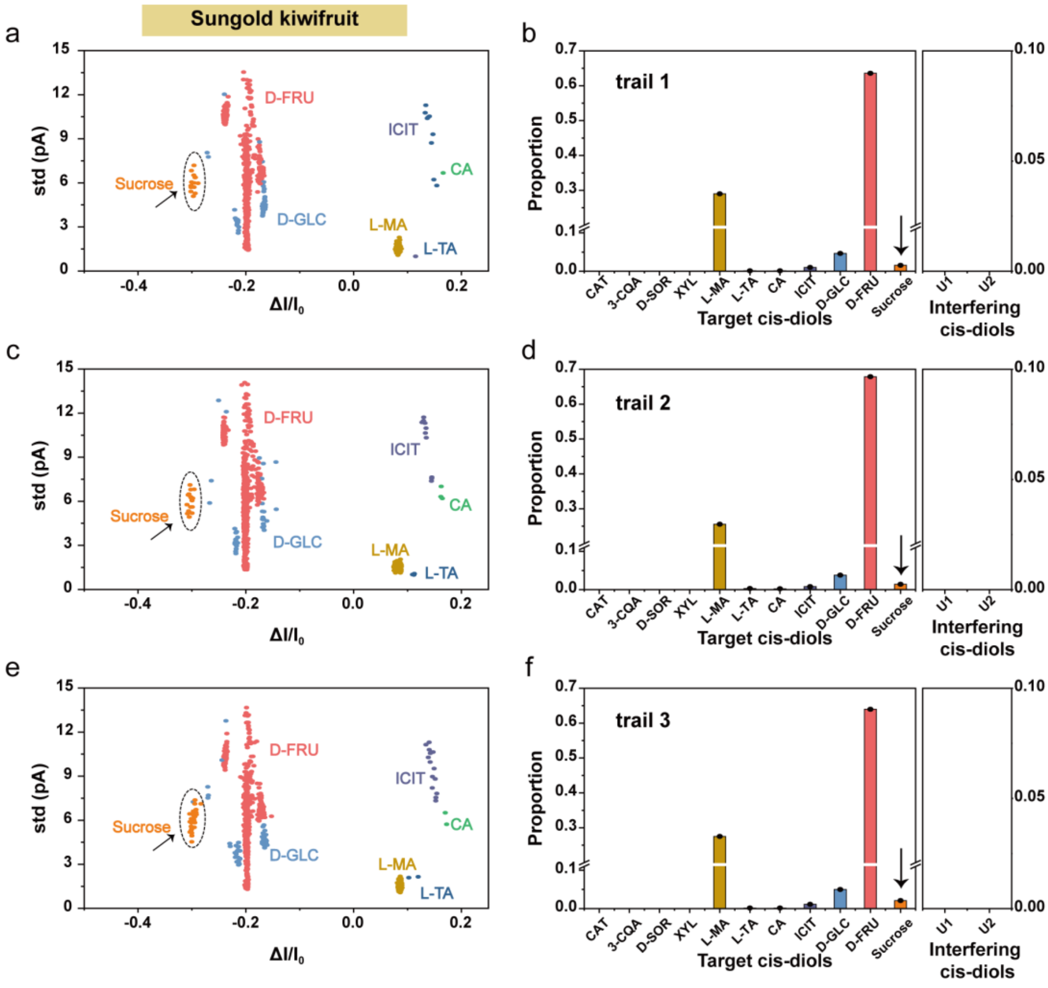

Supplementary Figure 53. Results of Sungold kiwifruit juice analysis. (a, c, e) The scatter plot of $\Delta I/I_0$ versus std for nanopore events acquired with Sungold kiwifruit juice. All background events were removed by cluster analysis (**Supplementary Fig. 47**) and outlier analysis (**Supplementary Fig. 47**). All events except sucrose were predicted and labeled by the previously trained Bagging Trees model (**Fig. 3**). The data presented in each scatter plot was from a continuous measurement of 60 min. **a)** 903 events, **c)** 1267 events and **e)** 1243 events were respectively included in each scatter plot. **(b, d, f)** The proportion of target *cis*-diol events in each corresponding set of scatter plot data. Results of all three independent measurements also demonstrate a high consistency. Data presented in each bar plot were from result acquired from one measurement (N=1). Results of three measurements were simultaneously presented to show data consistency. Results generated by sucrose events are marked with arrows. The nanopore measurements were performed using MspA-PBA in a 1.5 M KCl buffer. A bias of +160 mV was continually applied. To both the *cis* and *trans* chambers, 2 µL Sungold kiwifruit juice was respectively added and thoroughly stirred.

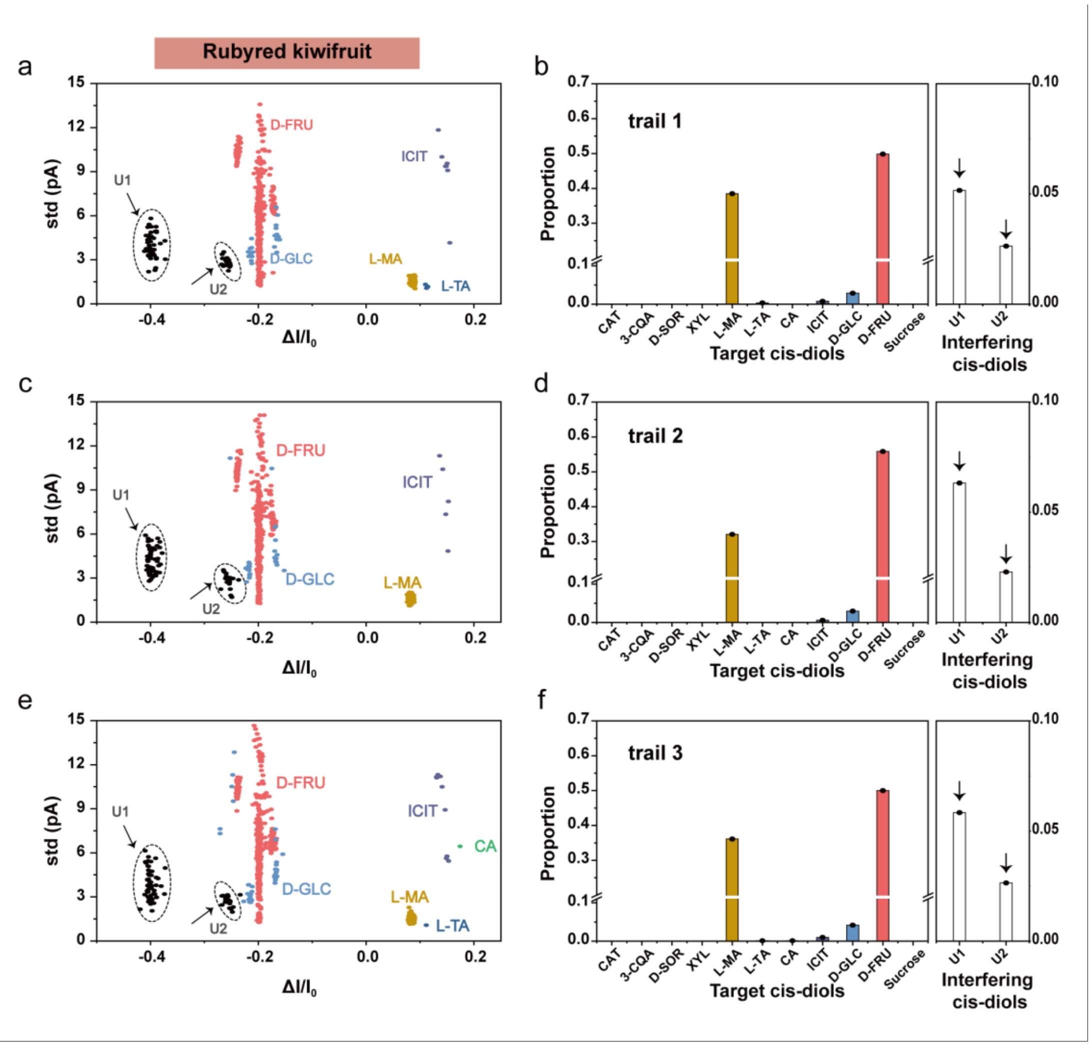

Supplementary Figure 54. Results of Rubyred kiwifruit juice analysis. (a, c, e) The scatter plot of $\Delta I/I_0$ versus std for nanopore events acquired with Rubyred kiwifruit juice. All background events were removed by cluster analysis (**Supplementary Fig. 48**) and outlier analysis (**Supplementary Fig. 48**). All events except U1 and U2 were predicted and labeled by the previously trained Bagging Trees model (**Fig. 3**). The data presented in each scatter plot was from a continuous measurement of 60 min. **a)** 950 events, **c)** 917 events and **e)** 908 events were respectively included in each scatter plot. **(b, d, f)** The proportion of target *cis*-diol events in each corresponding set of scatter plot data. Results of all three independent measurements also demonstrate a high consistency. Data presented in each bar plot were from results acquired from one measurement (N=1). Results of three measurements were simultaneously presented to show data consistency. Results of U1 and U2 are marked with arrows. The nanopore measurements were performed using MspA-PBA in a 1.5 M KCl buffer. A bias of +160 mV was continually applied. To both the *cis* and *trans* chambers, 2 µL Rubyred kiwifruit juice was respectively added and thoroughly stirred.

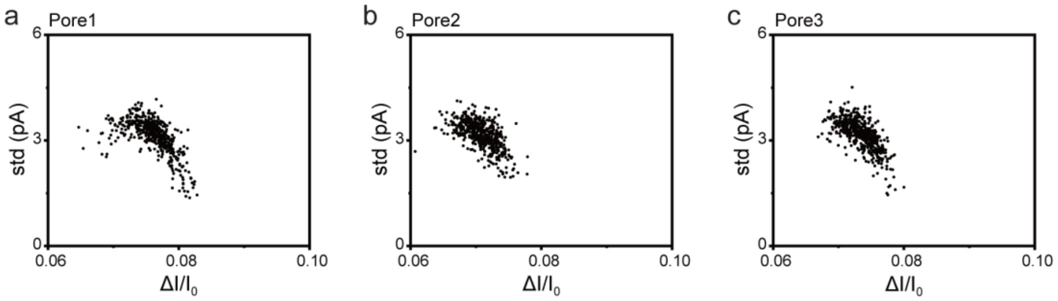

Supplementary Figure 55. Events of D-MA. (a-c) The scatter plots of $\Delta I/I_0$ versus std for nanopore events acquired with D-MA. Results in different scatter plots were respectively acquired from three independent measurements under the same measurement conditions. Each scatter plot contains 500 events. The nanopore measurements were performed using MspA-PBA in a 1.5 M KCl buffer. D-MA was added to *cis* and *trans* with a final concentration of 0.6 mM. A +160 mV bias was continually applied.

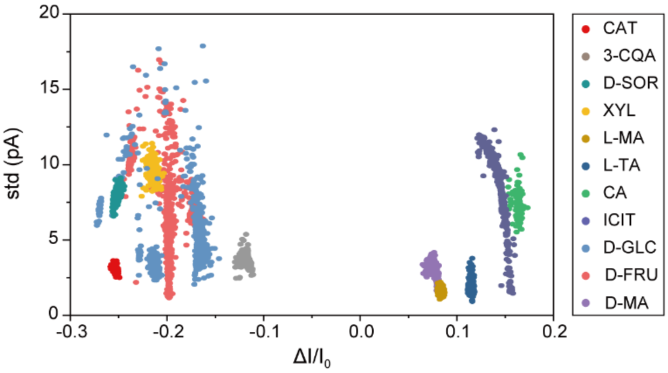

Supplementary Figure 56. The scatter plot of *ΔI/I₀* versus std for eleven target *cis*-diols. Events of eleven types of *cis*-diols were respectively acquired from eleven independent measurements (n = 1000 for D-FRU and D-GLC, n = 500 for CAT, 3-CQA, D-SOR, XYL, L-MA, D-MA, L-TA, ICIT, and n = 100 for CA). All *cis*-diols were measured using MspA-PBA in a 1.5 M KCl buffer. A bias of +160 mV was continually applied. The final concentration of each *cis*-diol was set as described in **Methods**.

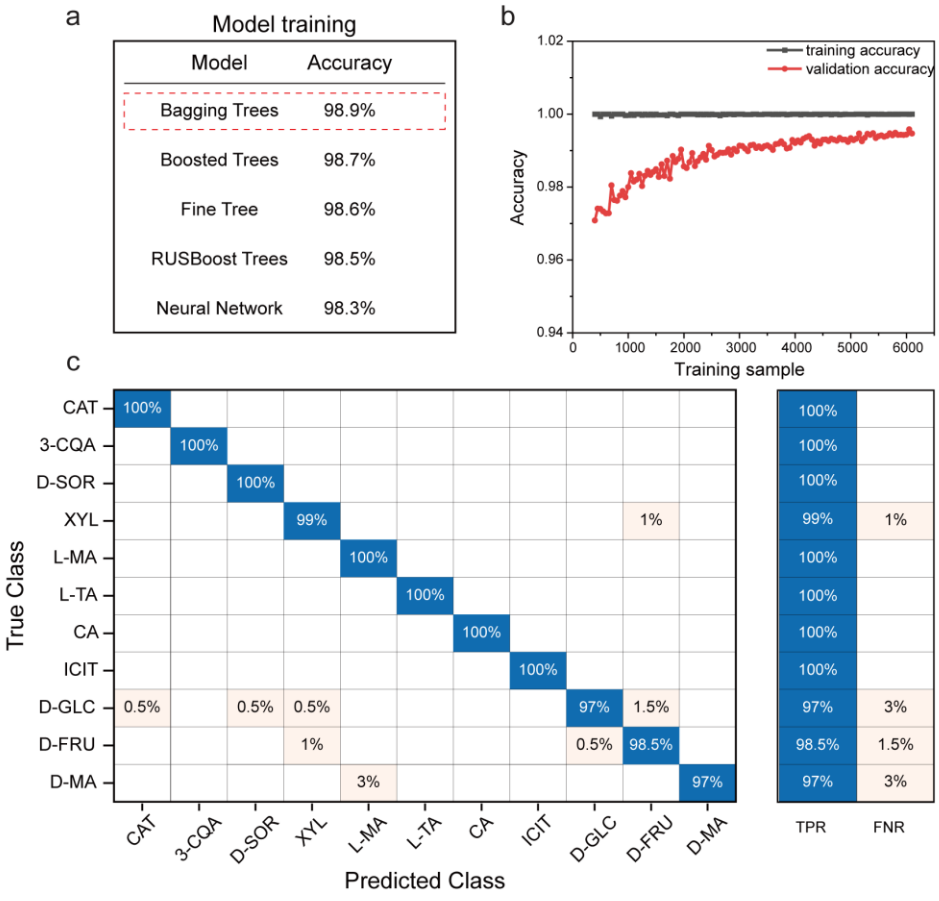

Supplementary Figure 57. Event identification by machine learning. (a) The validation accuracies of different models. The validation accuracies were evaluated by 10-fold cross-validation. After training with different models however with the same training set (**Supplementary Fig. 56**), the model of Bagging Trees still showed the highest accuracy of 98.9% and it was selected for subsequently prediction of results acquired with commercial juice and juice drink. **(b)** The learning curves of the Bagging Trees model generated by varying the size of the input training samples. The validation accuracy increases with an increasing size of the input training samples. Eventually, the validation accuracy gradually approached the training accuracy. Data in learning curves were presented as mean values derived from three independent measurements. **(c)** The confusion matrix plot of the testing set generated by the trained Bagging Trees model. The true positive rate (TPR) and the false negative rate (FNR) were demonstrated to the right.

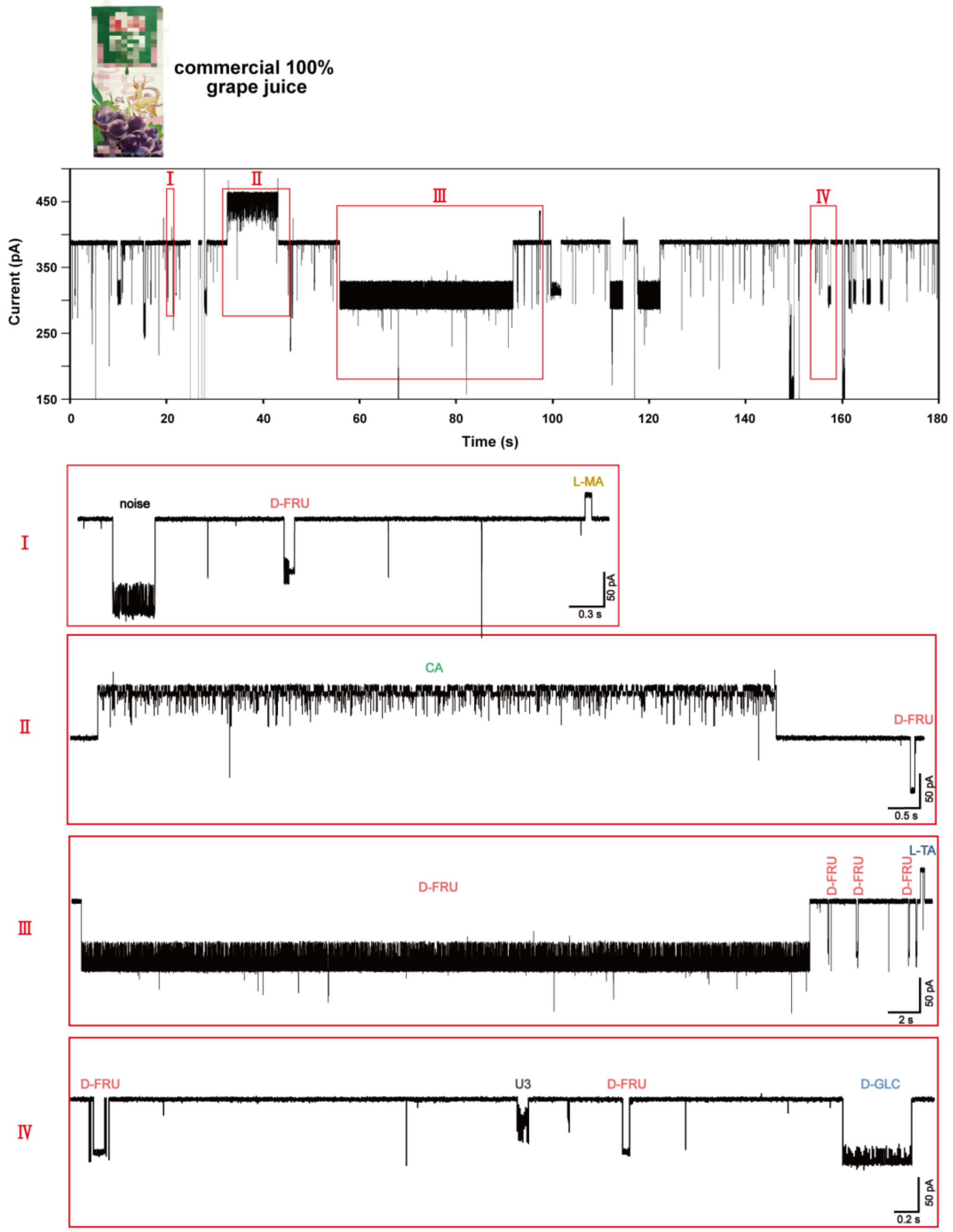

Supplementary Figure 58. Nanopore analysis of 100% grape juice. The nanopore measurements were performed using MspA-PBA in a 1.5 M KCl buffer. A bias of +160 mV was continually applied. 2 μL commercial 100% grape juice was added to both measurement chambers. A representative trace segment of 180 s acquired with commercial 100% grape juice was presented in the top. For a better demonstration of event details, trace sections marked with red boxes were zoomed in and demonstrated in the bottom. By machine learning prediction using the previously trained Bagging Trees model (**Supplementary Fig. 57**), events of L-MA, L-TA, CA, D-GLC, D-FRU and U3 (unidentified *cis*-diol 3) were clearly identified from the commercial 100% grape juice. Randomly appearing noise events, which don't show a consistent event feature, were also detected and labeled on the trace.

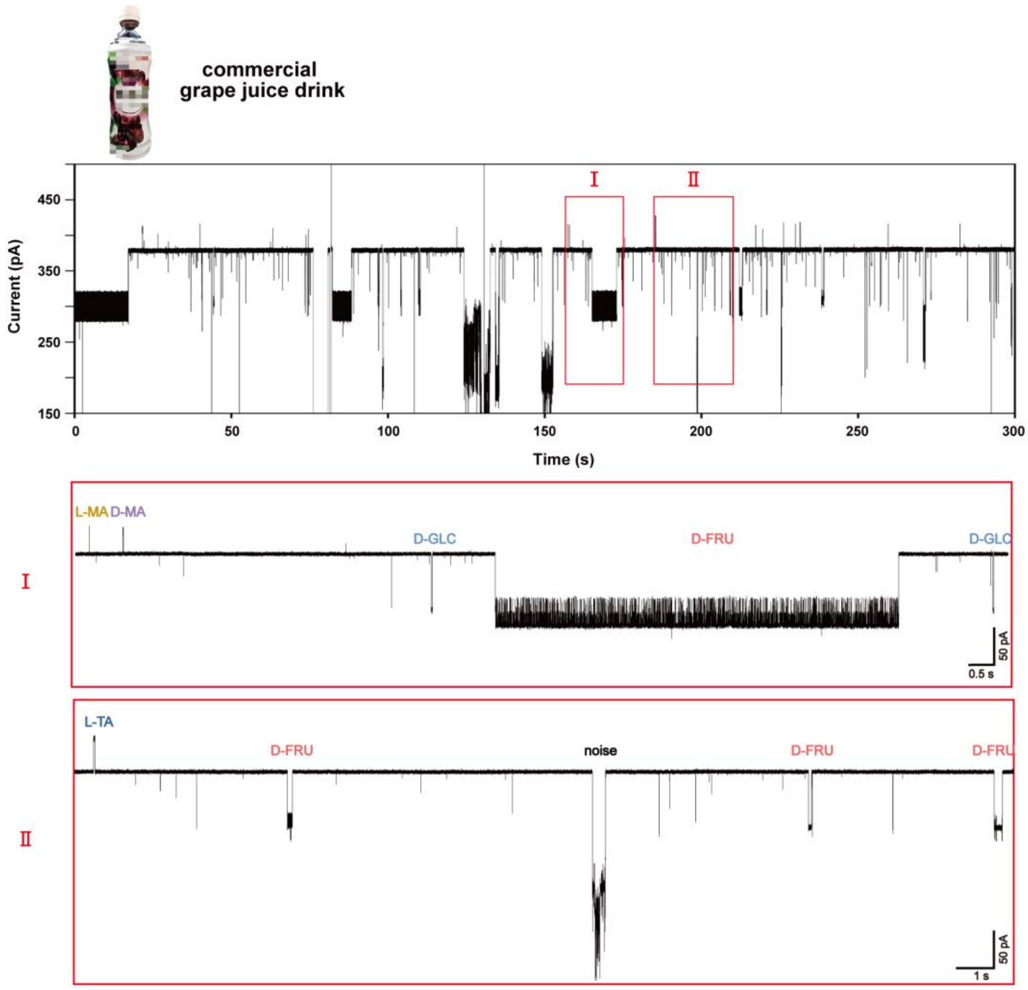

Supplementary Figure 59. Nanopore analysis of grape juice drink. The nanopore measurements were performed using MspA-PBA in a 1.5 M KCl buffer. A bias of +160 mV was continually applied. 2 μL commercial grape juice drink was added to both measurement chambers. A representative trace segment of 300 s acquired with commercial grape juice drink was presented in the top. For a better demonstration of event details, trace sections marked with red boxes were zoomed in and demonstrated in the bottom. By machine learning prediction using the previously trained Bagging Trees model (**Supplementary Fig. 57**), events of L-MA, D-MA, L-TA, D-GLC and D-FRU were clearly identified from the commercial grape juice drink. Randomly appearing noise events, which don't show a consistent event feature, were also detected and labeled on the trace.

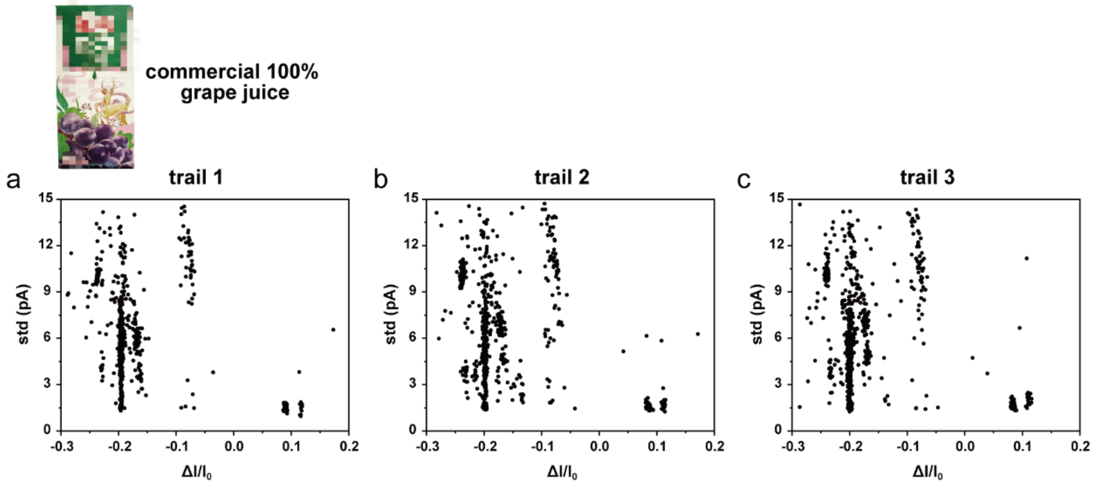

Supplementary Figure 60. Events of commercial 100% grape juice. (a-c) The scatter plots of $\Delta I/I_0$ versus std for nanopore events acquired with commercial 100% grape juice. Results in different scatter plots were respectively acquired from three independent measurements under the same measurement conditions. The nanopore measurements were performed using MspA-PBA in a 1.5 M KCl buffer. 2 μL commercial 100% grape juice was respectively added to both measurement chambers. A +160 mV bias was continually applied. Results in each scatter plot were from a 60 min continually recorded trace. **a)** 630 events, **b)** 755 events and **c)** 761 events were respectively included in each scatter plot.

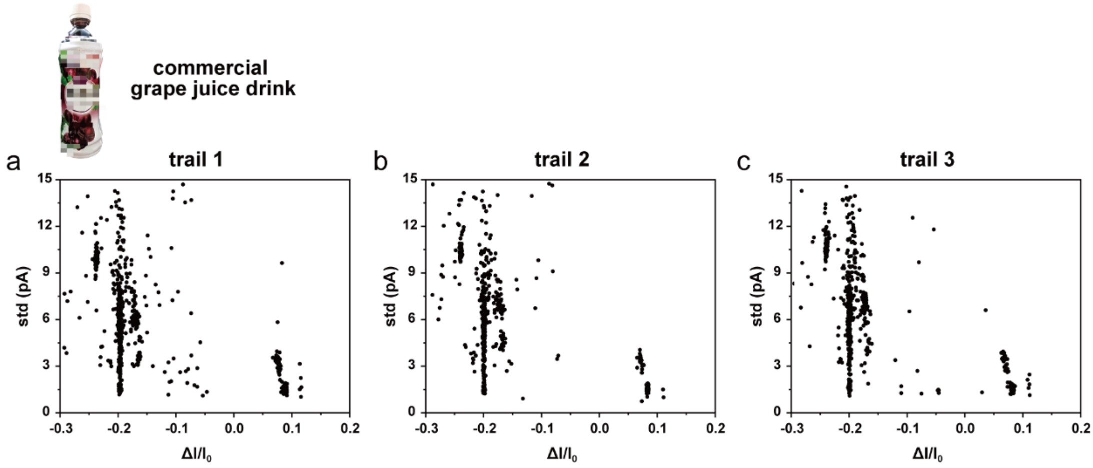

Supplementary Figure 61. Events of commercial grape juice drink. (a-c) The scatter plots of $\Delta I/I_0$ versus std for nanopore events acquired with commercial grape juice drink. Results in different scatter plots were respectively acquired from three independent measurements under the same measurement conditions. The nanopore measurements were performed using MspA-PBA in a 1.5 M KCl buffer. 2 μL commercial grape juice drink was respectively added to both measurement chambers. A +160 mV bias was continually applied. Results in each scatter plot were from a 60 min continually recorded trace. **a)** 762 events, **b)** 560 events and **c)** 622 events were respectively included in each scatter plot.

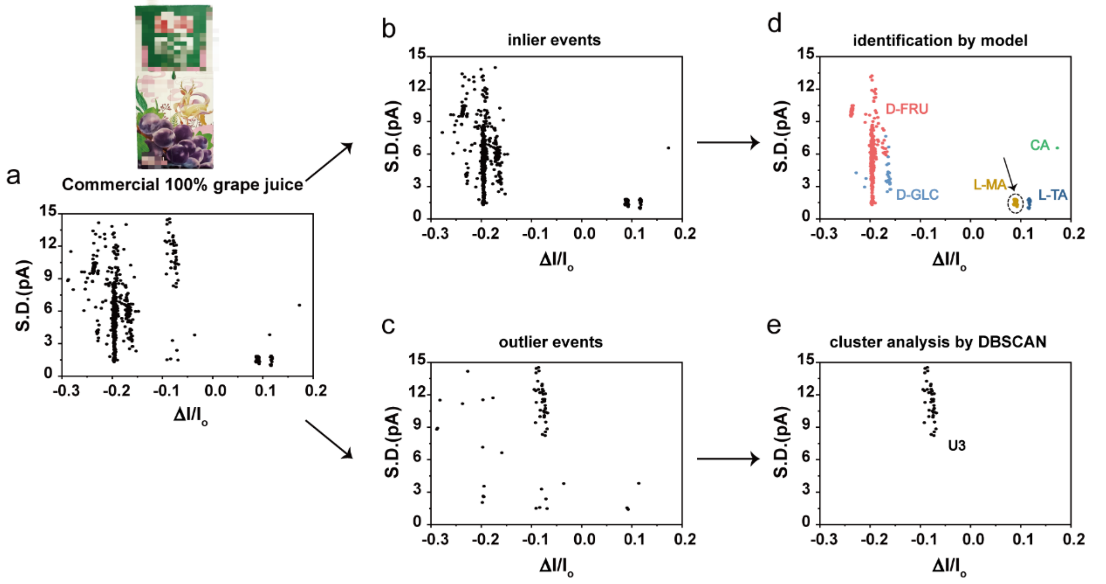

Supplementary Figure 62. The workflow of nanopore analysis of commercial 100% grape juice. (a) The scatter plot of *ΔI/I*$_0$ versus std for nanopore events acquired with commercial 100% grape juice **(Supplementary Fig. 60)**. The events were first divided into **(b)** inlier events and **(c)** outlier events using the One-Class SVM algorithm, an unsupervised machine learning algorithm applied for the detection of outlier events. **(b)** The scatter plot of *ΔI/I*$_0$ versus std for inlier events acquired with commercial 100% grape juice. **(c)** The scatter plot of *ΔI/I*$_0$ versus std for outlier events acquired with commercial 100% grape juice. **(d)** The scatter plot of *ΔI/I*$_0$ versus std for inlier events predicted by the previously trained Bagging Tress model **(Supplementary Fig. 57)**. Events of L-MA, L-TA, CA, D-GLC and D-FRU were identified from the commercial 100% grape juice. **(e)** The scatter plot of *ΔI/I*$_0$ versus std for cluster analysis of outlier events using DBSCAN, an unsupervised learning algorithm to identify clustering events in the outliers. The epsilon was set to 0.3 and the min_samples was set to 30. After the treatment, one cluster of events was detected and was marked as U3 (unidentified *cis*-diol 3). This event features of this cluster of events are completely different from any other previously investigated cis-diols using MspA-PBA. We thus conclude that this analyte, which produces U3 events, was only detected in commercial 100% grape juice, among all samples we have investigated in this paper.

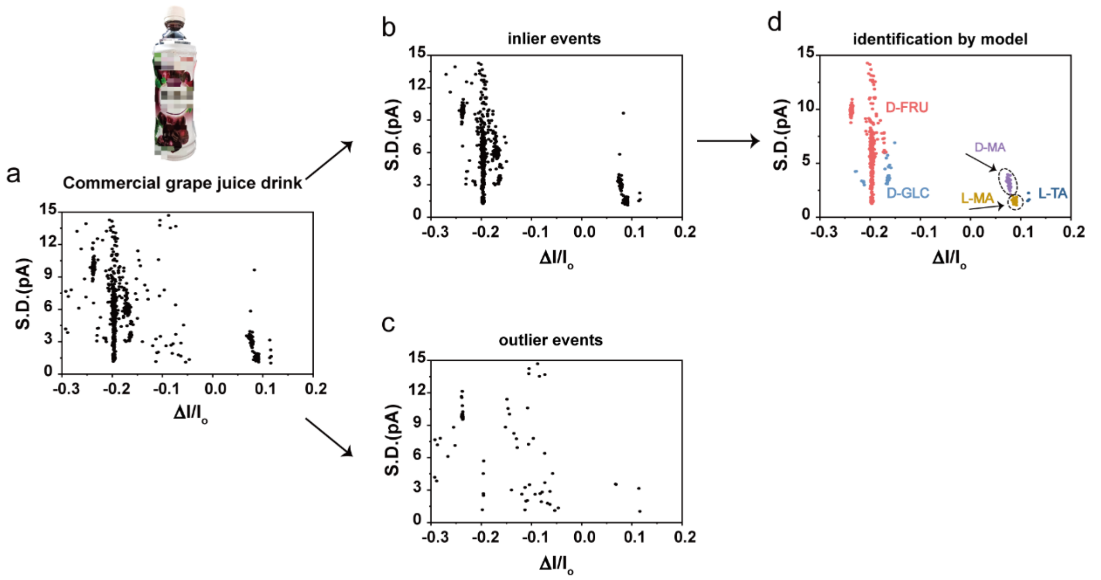

Supplementary Figure 63. The workflow of nanopore analysis of commercial grape juice drink. (a) The scatter plot of $\Delta I/I_0$ versus std for nanopore events acquired with commercial grape juice drink **(Supplementary Fig. 61)**. The events were first divided into **(b)** inlier events and **(c)** outlier events using the One-Class SVM algorithm, an unsupervised machine learning algorithm applied for the detection of outlier events. **(b)** The scatter plot of $\Delta I/I_0$ versus std for inlier events acquired with commercial grape juice drink. **(c)** The scatter plot of $\Delta I/I_0$ versus std for outlier events acquired with commercial grape juice drink. **(d)** The scatter plot of $\Delta I/I_0$ versus std for inlier events predicted by the previously trained Bagging Tress model **(Supplementary Fig. 57)**. Events of L-MA, D-MA, L-TA, D-GLC and D-FRU were identified from the commercial grape juice drink.

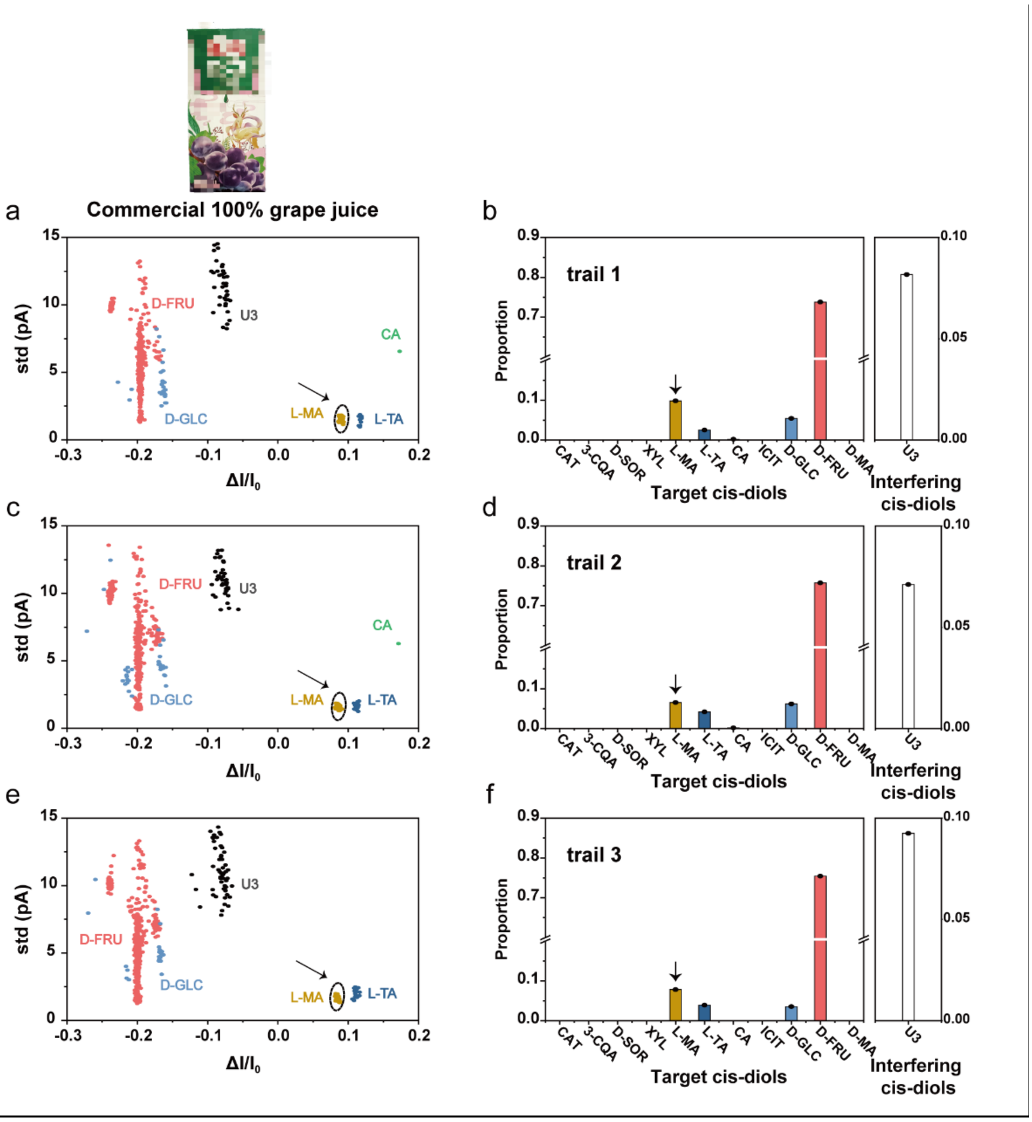

Supplementary Figure 64. Results of commercial 100% grape juice analysis. (a, c, e) The scatter plot of *$\Delta I/I_0$* versus std for nanopore events acquired with commercial 100% grape juice. All background events were removed by outlier analysis and cluster analysis **(Supplementary Fig. 62)**. All target *cis*-diol events were predicted and labeled by the previously trained Bagging Trees model **(Supplementary Fig. 57)**. The data presented in each scatter plot was from a continuous measurement of 60 min. **a)** 477 events, **c)** 548 events and **e)** 562 events were respectively included in each scatter plot. **(b, d, f)** The proportion of target *cis*-diol events in each corresponding set of scatter plot data. A high consistency of results is shown. Data presented in each bar plot were from results acquired from one measurement (N=1). Results of three measurements were simultaneously presented to show the consistency. Results generated by L-MA are annotated with arrows. The nanopore measurements were performed using MspA-PBA in a 1.5 M KCl buffer. A bias of +160 mV was continually applied. To both the *cis* and *trans* chambers, 2 µL 100% grape juice was respectively added and thoroughly stirred.

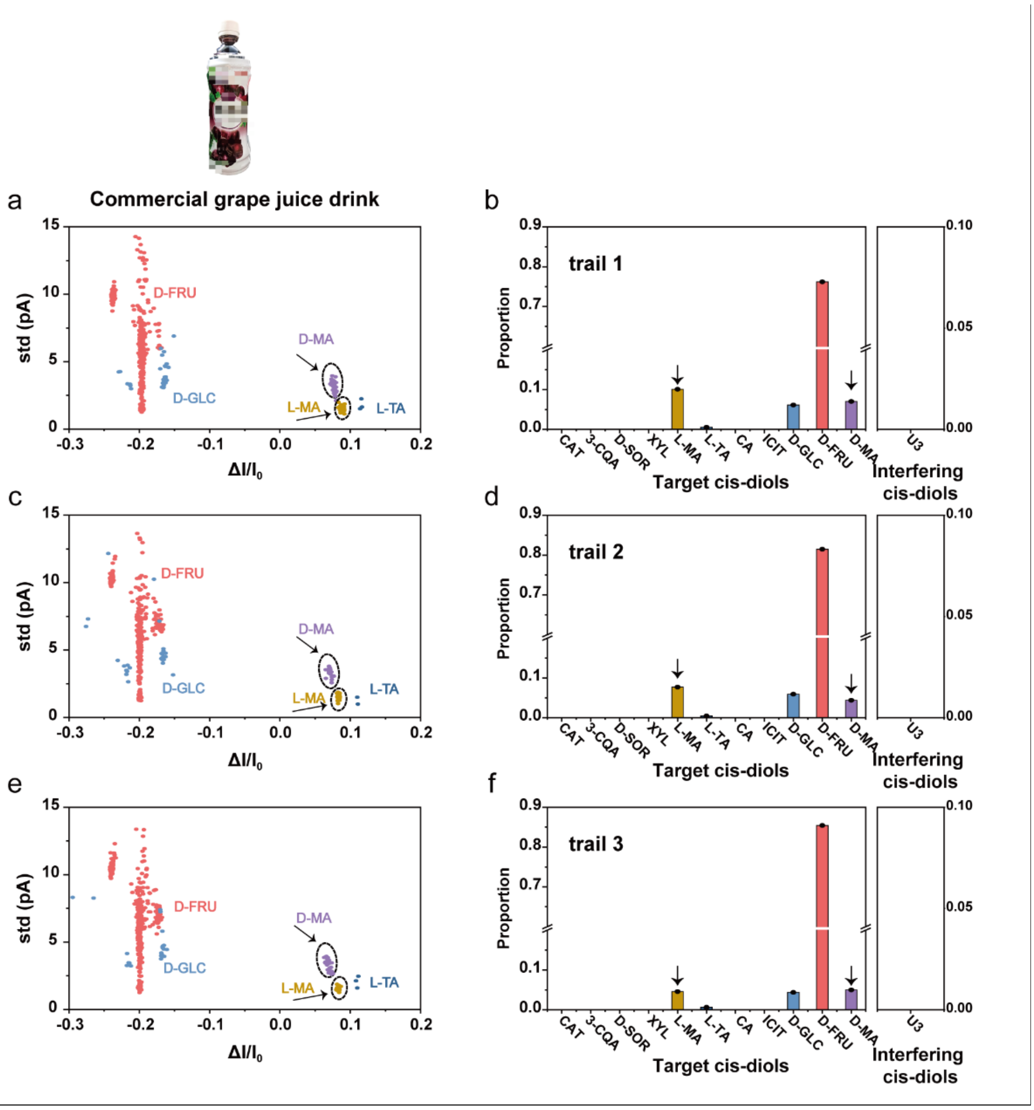

Supplementary Figure 65. Results of commercial grape juice drink analysis. (a, c, e) The scatter plot of $\Delta I/I_0$ versus std for nanopore events acquired with commercial grape juice drink. All background events were removed by cluster analysis **(Supplementary Fig. 63)**. All target *cis*-diol events were predicted and labeled by the previously trained Bagging Trees model **(Supplementary Fig. 57)**. The data presented in each scatter plot was from a continuous measurement of 60 min. **a)** 555 events, **c)** 454 events and **e)** 481 events were respectively included in each scatter plot. **(b, d, f)** The proportion of target *cis*-diol events in each corresponding set of scatter plot data. A high consistency of results is shown. Data presented in each bar plot were from results acquired from one measurement (N=1). Results of three measurements were simultaneously presented to show the consistency. Results generated by DL-MA are annotated with arrows. The nanopore measurements were performed using MspA-PBA in a 1.5 M KCl buffer. A bias of +160 mV was continually applied. To both the *cis* and *trans* chambers, 2 µL commercial grape juice drink was respectively added and thoroughly stirred.

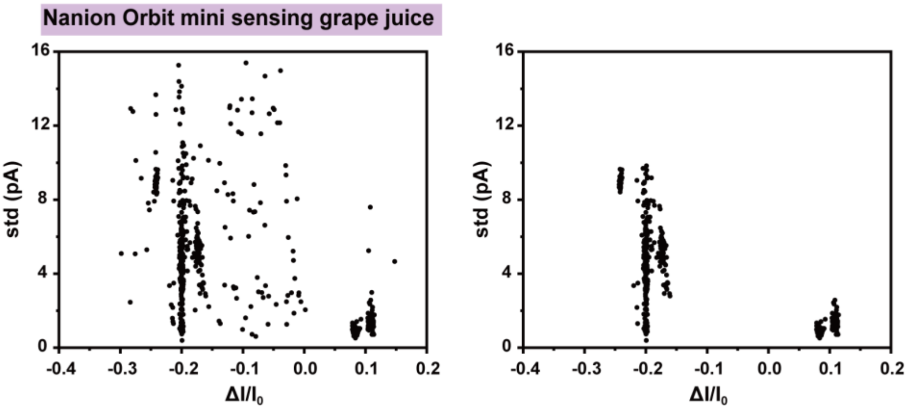

Supplementary Figure 66. Cluster analysis of grape juice acquired using Nanion Orbit mini. (Left) The scatter plot of $\Delta I/I_0$ versus std for nanopore events acquired with natural grape juice using Nanion Orbit mini (n=614). The nanopore measurements were performed using MspA-PBA in a 1.5 M KCl buffer. A bias of +160 mV was continually applied. 1 µL grape juice was added to the measurement device and thoroughly stirred. The data presented in scatter plot was from a continuous measurement of 40 min. Besides the data that form clear clusters in the event distribution, some randomly distributed events were also detected. **(Right)** Events after cluster analysis treatment (n=492). The cluster analysis, which removes non-clustered data points from the scatter plot, was performed by DBSCAN. The epsilon was set to 0.17 and the min_samples was set to 18.

Fruits morphology	Name of cultivar	Species	Origin	Brand	weight (g)
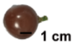	Kyoho grape	*Vitis Labrusca* × *Vinifera*	Liaoning Province, China	N.A.	15.26
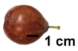	Chilean prunes	*Prunus domestica* L.	Chile	N.A.	23.03
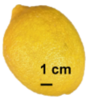	Eureke lemon	*Citrus limon* (L.) Burm. f.	Anyue County, China	Anyue Lemon	140.12
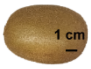	Green kiwifruit	*Actinidia deliciosa*	New Zealand	Zespri	125.22
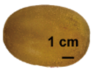	Sungold kiwifruit	*Actinidia chinensis*	New Zealand	Zespri	128.25
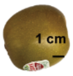	Rubyred kiwifruit	*Actinidia chinensis*	New Zealand	Zespri	110.12

N.A. stands for that the brand of this fruit is no avaliable

Supplementary Figure 67. Detailed parameters of fruit samples. The fruit samples used in this work include grape, prune, lemon and kiwifruits of different varieties. The corresponding morphology, the name of cultivar, the species, the origin, the brand and the average weight of each fruit were demonstrated in the table.

Supplementary References

1 Sivakumaran, S., Huffman, L., Sivakumaran, S. & Drummond, L. The nutritional composition of Zespri® SunGold Kiwifruit and Zespri® Sweet Green Kiwifruit. *Food Chemistry* **238**, 195-202, (2018).

2 Yuan, X. *et al.* Comparative Study on Physicochemical and Nutritional Qualities of Kiwifruit Varieties. *Foods* **12**, 108 (2023).
