# Supplementary material for: Nanopore analysis of cis-diols in fruits
Source: Nat Commun. 2024 Mar 5;15:1969. doi: 10.1038/s41467-024-46303-x (PMC10915164; doi:10.1038/s41467-024-46303-x)
Supplement: Supplementary file 3 — Description of additional supplementary files [file 41467_2024_46303_MOESM3_ESM.pdf]

DESCRIPTION OF ADDITIONAL SUPPLEMENTARY FILES DOCUMENT

Supplementary Movie 1. Nanopore analysis of prune juice. The nanopore measurements were performed using MspA-PBA in a 1.5 M KCl buffer. A bias of +160 mV was continually applied. 5 μL prune juice was respectively added to both measurement chambers. By machine learning prediction using the previously trained Bagging Trees model, events of 3-CQA, D-SOR, L-MA, DGLC and D-FRU can be identified and they were accordingly labelled on the trace. The movie was played back at 2.0× speed of the actual data acquisition.

Supplementary Movie 2. Nanopore analysis of grape juice. The nanopore measurements were performed using MspA-PBA in a 1.5 M KCl buffer. A bias of +160 mV was continually applied. 5 μL grape juice was respectively added to both measurement chambers. By machine learning prediction using the previously trained Bagging Trees model, events of L-MA, L-TA, D-GLC and D-FRU can be identified and they were accordingly labelled on the trace. The movie was played back at 2.0× speed of the actual data acquisition.

Supplementary Movie 3. Nanopore analysis of lemon juice. The nanopore measurements were performed using MspA-PBA in a 1.5 M KCl buffer. A bias of +160 mV was continually applied. 15 μL lemon juice was respectively added to both measurement chambers. By machine learning prediction using the previously trained Bagging Trees model, events of L-MA, CA, ICIT, D-GLC and D-FRU can be identified and they were accordingly labelled on the trace. The movie was played back at 2.0× speed of the actual data acquisition.
